# Bi-Polar Bioenergetic Intervention via a Pathology Self-Adaptive Single-Atom Nanocatalyst for Diabetic Tumor Postoperative Management

**DOI:** 10.1007/s40820-026-02169-w

**Published:** 2026-04-28

**Authors:** Jiajie Chen, Jimin Huang, Zhibo Yang, Kai Tang, Chengtie Wu, Huamao Ye, Jianlin Shi, Yufang Zhu

**Affiliations:** 1 State Key Laboratory of High Performance Ceramics, Shanghai Institute of Ceramics, Chinese Academy of Sciences, Shanghai, 200050 People’s Republic of China; 2 Center of Materials Science and Optoelectronics Engineering, University of Chinese Academy of Sciences, Beijing, 100049 People’s Republic of China; 3 Chair of Inorganic Chemistry I, Technische Universität Dresden, Bergstrasse 66, 01069 Dresden, Germany; 4 Shanghai Frontiers Science Center of Nanocatalytic Medicine, School of Medicine, Shanghai Tenth People’s Hospital, Tongji University, Shanghai, 200331 People’s Republic of China; 5 Department of Urology, Changhai Hospital, Second Military Medical University, Shanghai, 200433 People’s Republic of China; 6 School of Pharmacy, Xuzhou Medical University, Xuzhou, 221004 People’s Republic of China

**Keywords:** Single-atom nanocatalyst, Metabolic bifurcation, Bioenergetic intervention, Catalytic therapy, Diabetic tumor postoperative management

## Abstract

Diabetes aggravates postoperative tumor recurrence and impairs wound healing due to divergent metabolic adaptations to hyperglycemia in tumor versus normal cells. Current therapeutics fail due to the incapabilities in adaptively modulating the metabolic bifurcation across the contradictory pathological contexts. Here, we address this challenge by developing a platinum (Pt) single-atom nanocatalyst (PtSNC) with microenvironments-selective multienzyme-mimicking activities to pioneer a bi-polar bioenergetic intervention strategy. In acidic and reduced nicotinamide adenine dinucleotide (NADH)-overexpressed tumor niches, it exhibits NADH oxidase (NOX)-, oxidase-, and peroxidase-like activities, depleting NADH reserves and generating highly reactive Pt = O species to disrupt energy metabolism and induce both apoptosis/ferroptosis. While it shows NOX-, catalase-, and superoxide dismutase-like activities in neutral diabetic wounds, rectifying hyperglycemia-induced cellular NAD^+^/NADH abnormity and bioenergetic disorder to revitalize the cells and tissues, with promoting angiogenesis and mitigating local inflammation to accelerate regeneration. Murine diabetic melanoma resection models demonstrate its extensive capacity in effectively eradicating residual tumor tissues and suppressing the recurrence, and promoting diabetic wound healing concurrently without systemic toxicity, making PtSNC a safe and potent nanotherapeutic for diabetic tumor postoperative therapy. This study holds promise for the application of single-atom catalytic medicines in precision therapy for intractable diseases featuring pathological metabolic bifurcation.

**Supplementary Information:**

The online version contains supplementary material available at 10.1007/s40820-026-02169-w.

## Highlights

- A high-density and well-structured platinum single-atom nanocatalyst (PtSNC) with pathological contexts-selective multienzyme-mimicking activities is developed to pioneer a bi-polar bioenergetic intervention strategy and break the hyperglycemia-induced metabolic paradox.
- The PtSNC disrupts energy metabolism and triggers ferroptosis/apoptosis via depleting nicotinamide adenine dinucleotide (NADH) reserves and producing highly reactive Pt = O species in tumor niches, but rectifies cellular NAD^+^/NADH abnormity and bioenergetic disorder in diabetic wounds, offering a promising solution to diabetic tumor postoperative therapy.

**Supplementary Information:**

The online version contains supplementary material available at 10.1007/s40820-026-02169-w.

## Graphical abstract

**Figure:**
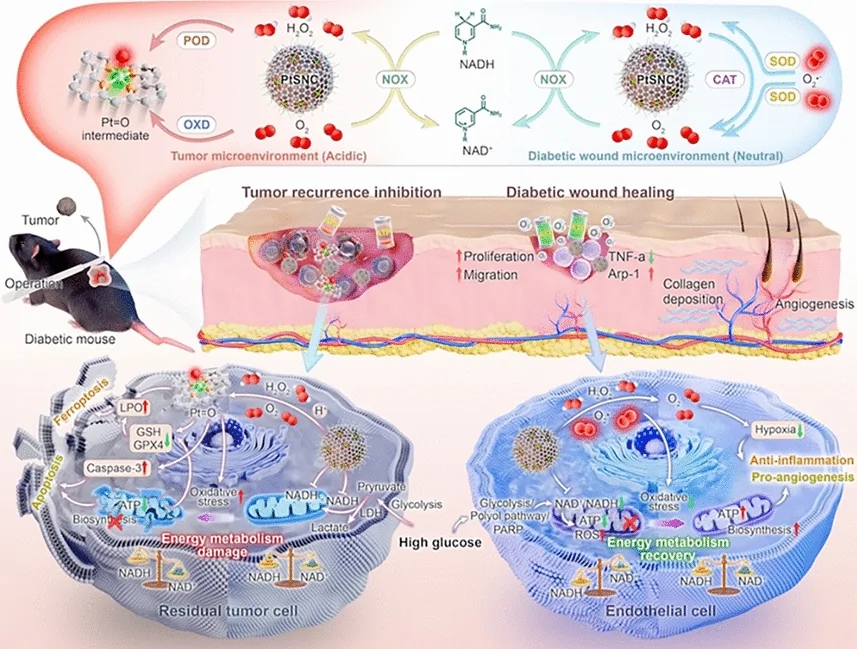

## Introduction

The global prevalence of diabetes has surged dramatically, affecting over 800 million adults and presenting a critical public health crisis [1]. As a multifaceted metabolic syndrome characterized by chronic hyperglycemia, diabetes frequently precipitates life-threatening complications [2]. In particular, diabetes elevates the risk of cancer incidence (ca. 20% ~ 25% higher than non-diabetic individuals [3]) and worsens the prognosis of various cancers such as breast, liver, colorectal, and melanoma malignancies [4–7]. For solid tumors like melanoma, surgery remains the primary treatment, yet it faces exacerbated challenges in diabetics, who undergoing tumor resection will struggle with chronic wound healing, susceptibility to infections, and rapid tumor recurrence, ultimately leading to therapeutic failure [4, 8–10]. Although current clinical treatments integrating glycemic control (e.g., gastric bypass surgery [11], hypoglycemic drugs) can partially optimize postoperative recovery, these approaches exhibit limited applicability, entail complex nutritional management, adverse side effects, depend heavily on tumor types, and critically, fail to effectively correct the local pathological microenvironment at the surgical site and suppress tumor recurrence/metastasis. Central to this unmet clinical need is the pathophysiological concept of “metabolic bifurcation” under hyperglycemia. Thus, it is imperative to establish an innovative strategy for postoperative management in diabetic cancer patients to accelerate chronic wound healing while restraining tumor recurrence.

This metabolic bifurcation describes the divergent metabolic adaptations of tumor versus normal cells to the same high glucose environment, leading to the paradoxical “pro-tumor, anti-healing” outcome. In diabetic wounds, elevated glucose levels disrupt the metabolic homeostasis of nicotinamide adenine dinucleotide (NAD, an essential cofactor) redox pairs (NAD^+^/NADH) through multiple pathways (e.g., glycolysis, polyol pathway, poly adenosine diphosphate (ADP) ribose polymerase (PARP) activation) in normal cells, typically characterized by NADH overload and NAD^+^ deficiency [12–14]. This imbalance dysregulates mitochondrial dynamics, not only impairing the tricarboxylic acid (TCA) cycle and oxidative phosphorylation (OXPHOS) to reduce adenosine triphosphate (ATP) synthesis but also generating deleterious reactive oxygen species (ROS) [14, 15]. The resultant bioenergetic chaos, oxidative stress, and inflammation severely impede wound healing [16, 17]. Conversely, in the tumor microenvironment (TME), tumor cells exploit glucose-rich environments to fuel aerobic glycolysis (the “Warburg effect”) and OXPHOS, meeting their high energy demands for promoting proliferation, invasion, migration, and meanwhile deploying robust antioxidant defense systems (e.g., overexpression of glutathione (GSH) [18]) to maintain redox homeostasis during fast metabolic processes [19–23]. Dealing with this bifurcated metabolic landscape requires a precisive approach capable of adaptively intervening in energy metabolism in both pathological contexts—a challenge unmet by current therapeutics.

Cellular metabolic pathways are usually regulated by enzymes such as oxidoreductases, hydrolases, lyase and so on, which can efficiently and specifically catalyze biochemical reactions [24]. However, the application of natural enzymes in disease treatments is hindered by instability, storage difficulties, and high costs. Recent advances in nanotechnology have spurred the development of nanocatalysts (or nanozymes) as enzyme mimics for metabolic modulation [25–28]. These synthetic nanocatalysts offer attractive advantages including low cost, high stability, scalable preparation, and tunable enzyme-like catalytic activities. Notably, single-atom nanocatalysts with maximized atomic utilization and enhanced catalytic performance have emerged as more promising substitutes for biomedical applications [29]. For example, the metal-nitrogen-carbon (M–N-C) nanomaterials with well-defined M-N_x_ coordination structure possess similar active sites to different natural enzymes, displaying high catalytic sensitivity and multienzyme-mimicking activities [30–34]. These advancements underscore the promise of tailored nanocatalysts for personalized metabolic modulation in precision medicine.

**Fig. 1:**
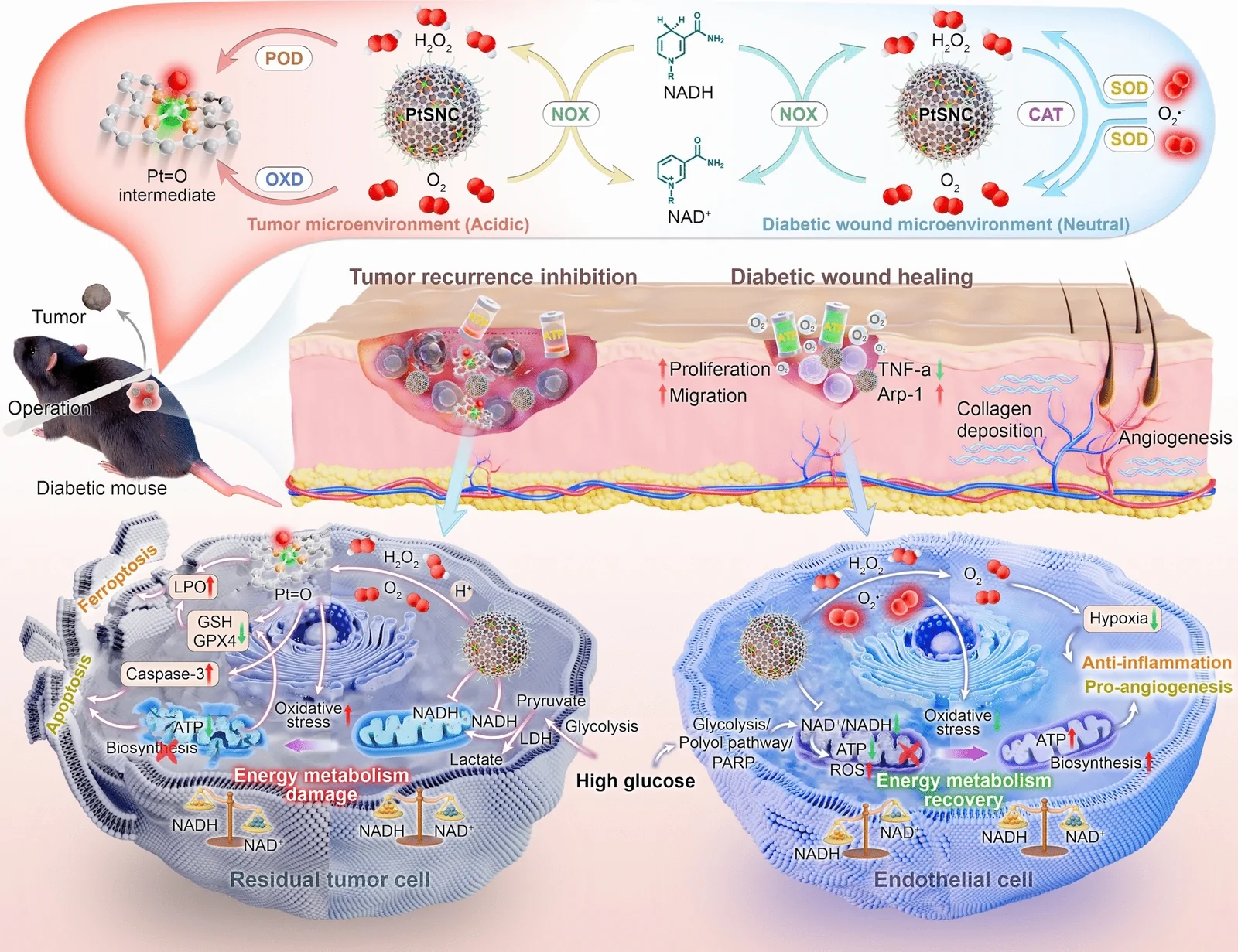
Schematic illustrations of the pathological contexts, self-selective multienzyme-mimicking catalysis of PtSNC, and the biological mechanisms for the diabetic tumor postoperative therapy. Under acidic conditions, PtSNC activates OXD-/POD-/NOX-like catalytic functions, depleting NADH and generating abundant highly reactive Pt = O species, while under neutral conditions, PtSNC exerts cascade SOD-/CAT-/NOX-like catalysis, scavenging hydrogen peroxide (H_2_O_2_) and superoxide anion (O_2_·^−^), replenishing O_2_, and continuously supplementing NAD^+^. In a diabetic melanoma postoperative model, on the one hand, PtSNC depletes NADH overaccumulation and leverages endogenous H_2_O_2_ and O_2_ to amplify Pt = O generation in residual tumor cells, synergistically perturbing redox homeostasis and inducing bioenergetic collapse, thereby triggering both lipid peroxidation (LPO)-mediated ferroptosis and apoptosis for tumor recurrence suppression; on the other hand, PtSNC restores high glucose-induced NAD^+^/NADH abnormity in endothelial cells while eliminating detrimental ROS and alleviating hypoxia, collectively rejuvenating cellular energy metabolism and biofunctions, suppressing inflammation, and promoting angiogenesis, ultimately accelerating diabetic surgical wound healing

Here, we formulate a biocompatible and multienzyme-mimicking platinum (Pt) single-atom nanocatalyst (PtSNC) with high-density Pt-N_4_ sites via a metal–organic framework (MOF)-based mixed-ligand strategy, exploiting its pathological contexts-selective multi-catalytic cooperativity to pioneer a bi-polar bioenergetic intervention strategy for diabetic melanoma postoperative therapy (Fig. 1). 1) In the TME featuring acidity and NADH overexpression [35, 36], PtSNC exerts NADH oxidase (NOX)-, oxidase (OXD)-, and peroxidase (POD)-like activities to efficiently deplete NADH reserves and generate highly reactive Pt = O intermediates, thereby disrupting mitochondrial electron transport chain (ETC), suppressing glycolysis/OXPHOS-driven ATP production, and destabilizing redox homeostasis. This induces residual tumor cells lack energy even under glucose-rich conditions and activates both apoptosis/ferroptosis, eventually suppressing melanoma relapse. 2) In the neutral diabetic wound milieu where normal cells suffer from the downregulation of NAD^+^/NADH ratio, PtSNC displays NOX-, catalase (CAT)-, and superoxide dismutase (SOD)-like activities, cascade restoring NAD^+^/NADH equilibrium, scavenging excess ROS, and alleviating hypoxia. This revitalizes normal cellular energy metabolism, promotes angiogenesis, mitigates local inflammation, and ultimately accelerates surgical skin wound healing. By adaptively targeting the hyperglycemia-induced metabolic bifurcation, this proposed strategy offers a highly specific, safe, and efficacious approach to simultaneously inhibit tumor recurrence and promote diabetic wound healing, presenting an unprecedented and inspiring therapeutic paradigm for clinical diabetic tumor postoperative management.

## Experimental Section

### Materials

Meso-tetra(4-carboxyphenyl) porphine (TCPP, ≥ 97%), zirconyl chloride octahydrate (ZrOCl_2_·8H_2_O, ≥ 98%), benzoic acid (C_7_H_6_O_2_, ≥ 99.5%), potassium persulfate (K_2_S_2_O_8_, ≥ 99%), 3,3',5,5'-tetramethylbenzidine (TMB, ≥ 98%), 1,2-diphenylisobenzofuran (DPBF, ≥ 97%), salicylic acid (C_7_H_6_O_3_, ≥ 99%), ascorbic acid (Vc, ≥ 99%), *β*-nicotinamide mononucleotide (NMN, ≥ 95%), and doxorubicin hydrochloride (DOX, ≥ 98%) were purchased from Shanghai Aladdin Biochemical Technology Co., Ltd. (China). *N*,*N*-Dimethylformamide (DMF, ≥ 99.5%), absolute ethanol (C_2_H_6_O, ≥ 99.7%), hydrofluoric acid (HF, ≥ 40%), and 30% (w/w) H_2_O_2_ aqueous solution were obtained from Sinopharm Group Chemical Reagent Co., Ltd. (China). DSPE-PEG_3400_-COOH was acquired from Shanghai Ponsure Biological Technology Co., Ltd. (China). Pt(II) meso-tetra(4-carboxyphenyl) porphine (TCPP(Pt), 95%) was provided by J&K Scientific Ltd. (Beijing, China). H_2_O_2_ assay kit, NAD^+^/NADH assay kit with WST-8, total SOD assay kit with WST-8, ATP assay kit, Z-VAD-FMK, 2',7'-dichlorofluorescin diacetate (DCFH-DA), GreenNuc™ Caspase-3 assay kit, and dihydroethidium (DHE) were purchased from Beyotime Institute of Biotechnology (China). JC-1 and necrostatin-1 were obtained from Solarbio Science & Technology Co., Ltd. (Beijing, China). NADH, 2,2'-azino-bis(3-ethylbenzothiazoline-6-sulfonic acid) diammonium salt (ABTS, ≥ 98%), ferrostatin-1, *N*-acetyl-L-cysteine (NAC), reduced glutathione (GSH), and 5,5'-dithiobis(2-nitrobenzoic acid) (DTNB) were obtained from Sigma-Aldrich (USA). FK866 was provided by Selleck Chemicals (USA). BODIPY™ 581/591 C11 was purchased from Thermo Fisher Scientific Inc. (USA). All the antibodies used in this study were listed in Table S3. Water purified with a Smart-Q15 laboratory water purification system was used throughout the experiments.

### Synthesis of PtSNC

The PCN-224-Pt_20_ precursor was synthesized by dissolving TCPP (80 mg), TCPP(Pt) (20 mg), ZrOCl_2_·8H_2_O (300 mg), and benzoic acid (3.0 g) in 100 mL *N*,*N*-Dimethylformamide (DMF). The mixture was homogenized by ultrasonication and stirred at 90 °C for 5 h. The resulting purple precipitate was collected by centrifugation (11,000 rpm, 30 min), sequentially washed twice with DMF and ethanol, and vacuum dried overnight. The dried purple powder was ground uniformly and subjected to pyrolysis in a tubular furnace under N_2_ at 800 °C (a heating rate of 5 °C min^−1^, holding for 2 h). The black residue was ultrasonically dispersed in 20 wt% hydrofluoric acid (HF) solution and incubated at 60 °C for 5 h to remove ZrO_2_ crystals, washed twice with ethanol and deionized water, and dried to obtain raw PtSNC. The PCN-224-Pt_0_ and PCN-224-Pt_40_ were prepared analogously by adjusting the TCPP/TCPP(Pt) ratios to 100/0 mg and 60/40 mg, respectively, followed by identical pyrolysis and acid etching procedures. For PEGylation, 5 mg of raw PtSNC was dispersed in 1 mL chloroform, mixed with 2.5 mg of DSPE-PEG_3400_-COOH, and sonicated for 30 min. The PEGylated PtSNC was isolated by centrifugation, air-dried to remove residual chloroform, washed with deionized water, and finally stored in 1 mL deionized water for subsequent experiments. The used characterizations are described in the Supplementary Methods.

### Multienzyme-Mimicking Activities of PtSNC

The NOX-like catalytic ability was first assessed by monitoring NADH oxidation at its 340 nm characteristic absorption. The test solutions containing NADH (0.2 mM) and PtSNC (0, 50, 100, and 200 μg mL^−1^) were incubated in a table concentrator at 37 °C for 8 h. Aliquots were centrifuged at scheduled intervals, and NADH concentrations in the supernatants were quantified via a pre-calibrated standard curve. To explore the pH effect, PtSNC (200 μg mL^−1^) and NADH (0.2 mM) were mixed in the sodium acetate buffers (pH 4.0, 5.2, 6.0, 7.4, 8.5, and 9.0) at 37 °C for 8 h and analyzed identically. To evaluate O_2_ involvement, the NADH solution were purged with Ar (10 min) prior to adding PtSNC. The reaction was conducted in a vacuum oven at 37 °C, with the non-purged sample incubated under an ambient air condition as a control. For NAD^+^ product verification, alcohol dehydrogenase (ADH), which can specifically reduce NAD^+^ to NADH, was introduced. Post-reaction supernatants (PtSNC + NADH, 4 h incubation) were treated with ADH solution (from a NAD^+^/NADH assay kit) or deionized water for 30 min at 37 °C. The NADH levels were determined using kit-specific chromogenic reagent. Finally, H_2_O_2_ generation during this catalysis was measured colorimetrically using a H_2_O_2_ assay kit following the manufacturer’s instruction.

The CAT-mimicking activity of PtSNC was tested by monitoring O_2_ production during H_2_O_2_ decomposition. The reaction solutions containing PtSNC (25 μg mL^−1^) and different concentrations of H_2_O_2_ (0, 2.5, 5, and 10 mM) were incubated at 37 °C. Besides, PtSNC and H_2_O_2_ (10 mM) were treated in the sodium acetate buffers (pH 4.0–9.0) at 37 °C. O_2_ generation during catalysis was quantified in real-time using a dissolved oxygen meter.

Using a total SOD assay kit (WST-8 method) to evaluate the SOD-mimicking activity of PtSNC according to the manufacturer’s instruction. The PtSNC samples (0–200 μg mL^−1^) were treated with WST-8/enzyme working solution and reaction initiator in sequence, incubated at 37 °C for 30 min, and analyzed at 450 nm.

Both OXD and POD-like catalytic abilities were assessed using TMB as a colorimetric indicator. For OXD-like activity assay, PtSNC (25 μg mL^−1^) was dispersed in the sodium acetate buffers (20 mM, pH 5.2) containing TMB at varying concentrations (50, 100, 200, and 400 μM). O_2_ dependency was evaluated by comparing Ar-purged (10 min) and ambient air conditions. For POD-like activity assay, PtSNC (25 μg mL^−1^) was dispersed in the sodium acetate buffers (pH 5.2) containing TMB (50, 100, 200, and 400 μM) and H_2_O_2_ (10 mM). Additional experiments were conducted with fixed TMB (200 μM) and varying H_2_O_2_ concentrations (5, 10, 20, and 40 mM). The pH sensitivity of both activities was tested in the sodium acetate buffers (pH 4.0–9.0). All reactions were initiated by adding PtSNC, and real-time oxidation kinetics were monitored by measuring the absorbance of blue oxTMB at 654 nm. Michaelis–Menten parameters (*K*_m_, *V*_max_, and *k*_cat_) were derived from Lineweaver–Burk double-reciprocal plots generated in Origin 2018 (OriginLab, USA). The catalytic rate constant *k*_cat_ was calculated using the following equation:

$$ k_{cat} = V_{\max } /\left[ {E_{0} } \right] $$

where [E_0_] represents the molar concentration of catalytically active metal sites, determined by ICP-MS.

### Cells and Animals

HDFs, HUVECs, HaCaTs, and B16F10 cells were obtained from the Cell Bank of the Chinese Academy of Sciences (Shanghai, China). B16F10 cells, HDFs, and HaCaTs were cultured in Dulbecco’s modified Eagle medium (DMEM, Gibco) supplemented with 10% (v/v) fetal bovine serum (FBS) and 1% (v/v) penicillin/streptomycin (P/S). HUVECs were maintained in endothelial cell medium (ECM, ScienCell, USA) containing 5% (v/v) FBS, 1% (v/v) P/S, and 1% (v/v) endothelial cell growth factor. All cells were incubated at 37 °C in a humidified atmosphere with 5% CO_2_.

Specific pathogen-free (SPF) male BALB/c nude mice (4–6 weeks old) and C57BL/6 mice (6–8 weeks old) were provided by Shanghai Legen Biotechnology Co., Ltd. (China). All animal experiments were conducted in accordance with the guidelines approved by the Animal Care and Laboratory Committee of the Sixth People’s Hospital Affiliated to Shanghai Jiao Tong University School of Medicine (approval no. DWSY2022-0018).

### Biocompatibility of PtSNC

HUVECs, HDFs, and HaCaTs were seeded in 96-well plates at 1 × 10^4^ cells per well and cultured for 24 h. The medium was replaced with 100 μL fresh medium containing varying concentrations of PtSNC, followed by 24 h incubation. Cell viability was quantified by typical cell counting kit-8 (CCK-8) assay, with absorbance measured at 450 nm.

### Cell Viabilities of Different Cells Under Varying Glucose Contents

To investigate the effects of glucose concentration on the cell viabilities of HUVECs and B16F10 cells, these cell lines were cultured in the ECM and low-glucose DMEM, respectively. Basal glucose levels were adjusted from about 5.5 mM (NG condition) to 10, 15, 20, 25 (HG condition), and 30 mM by supplementation. After 48 h incubation, viabilities were assessed by CCK-8 assay. Unless otherwise specified, B16F10 cell line was routinely cultured in HG DMEM to meet high nutritional requirements for experimental studies.

### Detection of Cellular NAD^+^/NADH Ratio and ATP Level

To investigate the NOX-like activity of PtSNC in cells, HUVECs, HDFs, and HaCaTs were seeded in 6-well plates and cultured for 24 h. The medium was replaced with fresh medium containing varying concentrations of PtSNC for 24 h. Intracellular NAD^+^ content, NADH content, and NAD^+^/NADH ratios were quantified using a NAD^+^/NADH assay kit with WST-8, following the manufacturer’s protocol.

To explore the effects of glucose concentration on the cellular NAD^+^/NADH ratios and ATP levels, HUVECs or B16F10 cells were cultured for 48 h in the ECM or low-glucose DMEM with graded glucose levels (5.5 mM (NG), 10, 15, 20, 25 (HG), and 30 mM). The relative NAD^+^/NADH ratios and ATP levels were measured using NAD^+^/NADH and ATP assay kits, respectively.

Furthermore, the bioenergetic modulation by PtSNC in normal and tumor cells was analyzed. In details, HUVECs were preconditioned in the HG medium for 48 h (the NG medium as a control), followed by 5-day treatments: 1) NG; 2) HG; 3) HG + 0.1 mM Vc; 4) HG + 8 μg mL^−1^ PtSNC. In addition, the 8 μg mL^−1^ PtSNC replenishment was conducted on day 4. The relative NAD^+^/NADH ratios were monitored daily, while the ATP levels were quantified on day 3. Differently, B16F10 cells in the HG medium were treated with varying concentrations of PtSNC for 24 h. The relative NAD^+^/NADH ratios and ATP levels were tested as above.

### Analysis of Cellular OCR

Cellular OCR was measured using Seahorse XFe96 Analyzer (Agilent Technologies, USA) with Seahorse XF Cell Mito Stress Test Kit (#103015-100). The HUVECs and B16F10 cells post-different treatments were seeded in XF96-well plates at 1 × 10^4^ cells per well and incubated overnight. Sensor cartridge was hydrated in 37 °C ultrapure water. On the day of assay, the cell culture plate was replaced with the detection medium (DMEM + 1 mM pyruvate + 2 mM glutamine + 10 mM glucose) and these cells were equilibrated in a CO_2_-free incubator for 60 min. According to the manufacturer’s instruction, sequential injections including 1 μM oligomycin (Complex V inhibitor), 1 μM carbonyl cyanide-4 (trifluoromethoxy) phenylhydrazone (FCCP, uncoupler), and 1 μM rotenone/antimycin A (Rot/AntA, Complex I/III inhibitors) were conducted for Seahorse program measurement. The obtained OCR profiles were analyzed using Seahorse Wave desktop software (Agilent Technologies, USA).

### Transcriptomic Sequencing Analysis

The HUVECs and B16F10 cells post-different treatments were treated with TRIzol reagent to extract RNA (*n* = 3). The subsequent RNA sequencing was performed by Majorbio Bio-Pharm Technology Co., Ltd (Shanghai, China) following their standardized Illumina NovaSeq X Plus platform protocol. The data were processed and analyzed using the online Majorbio Cloud Platform (www.majorbio.com). DEGs were identified with a threshold of *P* value < 0.05 and |log1.5 Fold Change|> 1. Functional enrichment analysis of DEGs was conducted via KEGG and GO databases.

### In Vivo Diabetic Wound Healing Evaluation

For diabetic model building, C57BL/6 mice received intraperitoneal injections of STZ (50 mg kg^−1^ day^−1^) for 5 consecutive days. After 30 days, full-thickness circular dorsal skin wounds (10 mm diameter) were surgically created on non-diabetic and as-established diabetic mice. Mice were randomized into four groups (*n* = 5): 1) NG (non-diabetic mice + PBS); 2) HG (diabetic mice + PBS); 3) HG + Vc (diabetic mice + 0.1 mM Vc); 4) HG + PtSNC (diabetic mice + 50 μg mL^−1^ PtSNC). The treatments were administered intradermally to the wounds by spray followed by occlusive dressing on days 0, 3, and 6. Wound areas were photographed and blood glucose levels and body weights were measured at days 0, 3, 6, 9, and 15. In the parallel experiments, the newly formed skin tissues in the wounds were harvested at days 3, 6, 9, and 15, and homogenized for detection of the NAD^+^/NADH ratios and ATP levels using corresponding test kits. In addition, on day 1, the wound skin tissues were harvested, frozen and sliced for DHE-based O_2_•^−^ detection. On days 6 and 15, the wound skin tissues were harvested for histological analyses, including H&E staining (days 6 and 15), Masson staining (days 6 and 15), inflammatory factors Arg-1/TNF-α immunohistochemistry (day 6), HIF-1α (day 6) and CD31 (day 15) immunofluorescence. After 15 days of treatment, blood from each group (*n* = 3) was collected for hematological analysis and liver/kidney function tests, and major organs (heart, liver, spleen, lung, and kidney) were harvested for H&E staining analysis.

### In Vivo Tumor Recurrence Inhibition/Wound Healing Assessments on a Diabetic Tumor Postoperative Model

B16F10 cells were intradermally inoculated into the dorsal skin of STZ-induced diabetic C57BL/6 mice three days prior to surgery. On day 0, ~ 95% of the tumor tissue was surgically resected, and a full-thickness circular skin wound (10 mm diameter) was created adjacent to the residual tumor for diabetic melanoma postoperative model building. Mice were randomized into five groups (*n* = 5): 1) NG (non-diabetic mice + PBS); 2) HG (diabetic mice + PBS); 3) HG + Vc (diabetic mice + 0.1 mM Vc); 4) HG + DOX (diabetic mice + 0.1 mM DOX); 5) HG + PtSNC (diabetic mice + 50 μg mL^−1^ PtSNC). The treatments were administered as same as the in vivo wound healing assay. Wound areas were photographed and tumor volumes (calculated as V = 0.5 × width^2^ × length)/blood glucose levels/body weights were measured at days 0, 3, 6, 9, and 12. On day 12, mice were euthanized and their recurrent tumors were harvested, weighed, and finally sectioned for H&E/Ki67/TUNEL staining analyses. Besides, major organs were harvested for H&E staining analysis. In the parallel experiments, the treated mice were monitored for 30 days, with survival defined as recurrent tumor volume ≤ 1000 mm^3^.

### In Vivo Biosafety and Biodistribution Analyses

To comprehensively assess the in vivo long-term biosafety of PtSNC, an extended study was conducted in the STZ-induced diabetic C57BL/6 mice. These mice were randomized into three groups (*n* = 3) and intravenously injected with PtSNC at doses of 0 (as the control group), 100, or 500 μg mL^−1^ via the tail vein. A repeat-dose regimen was employed, with injections administered on day 0 and day 14, and a total observation period of one month. Throughout the experiment, body weight and blood glucose levels were measured at days 0, 7, 14, 21, 26, and 31. At the experimental endpoint (day 31), blood was collected for hematological analysis and liver/kidney function tests. In addition, major organs were harvested for H&E staining analysis.

Biodistribution and clearance profiling was performed using the Cy5 fluorescence dye-labeled PtSNC. Diabetic mice were intravenously injected with Cy5-PtSNC (500 μg mL^−1^). In vivo fluorescence imaging was performed at 2, 12, and 24 h post-systemic injection using an In Vivo Imaging System (IVIS, PerkinElmer). At each time point, mice were euthanized for ex vivo imaging. Major organs and feces were collected and imaged immediately to quantify the fluorescence signal distribution.

### Statistical Analysis

Statistical analyses were performed using Origin 2018 and Microsoft Excel 2016. ImageJ (version 1.8.0) was employed to quantify mean fluorescence intensity (MFI), mitochondrial continuity, regional area, and cell count from microscopy images. The results obtained from independent biological replicates (*n* ≥ 3) were expressed as means ± standard error of the mean (s.e.m.). The significance of the difference was determined as follows: two-group comparisons—two-tailed Student’s *t*-test; multi-group comparisons—one-way analysis of variances (ANOVA) with Tukey’s *post-hoc* test (single variable) or two-way ANOVA with Bonferroni’s *post-hoc* test (multiple variables). The *P* values less than 0.05 were considered statistically significant.

## Results and Discussion

### Synthesis and Characterization of Pt Single-Atom Nanocatalyst PtSNC

**Fig. 2:**
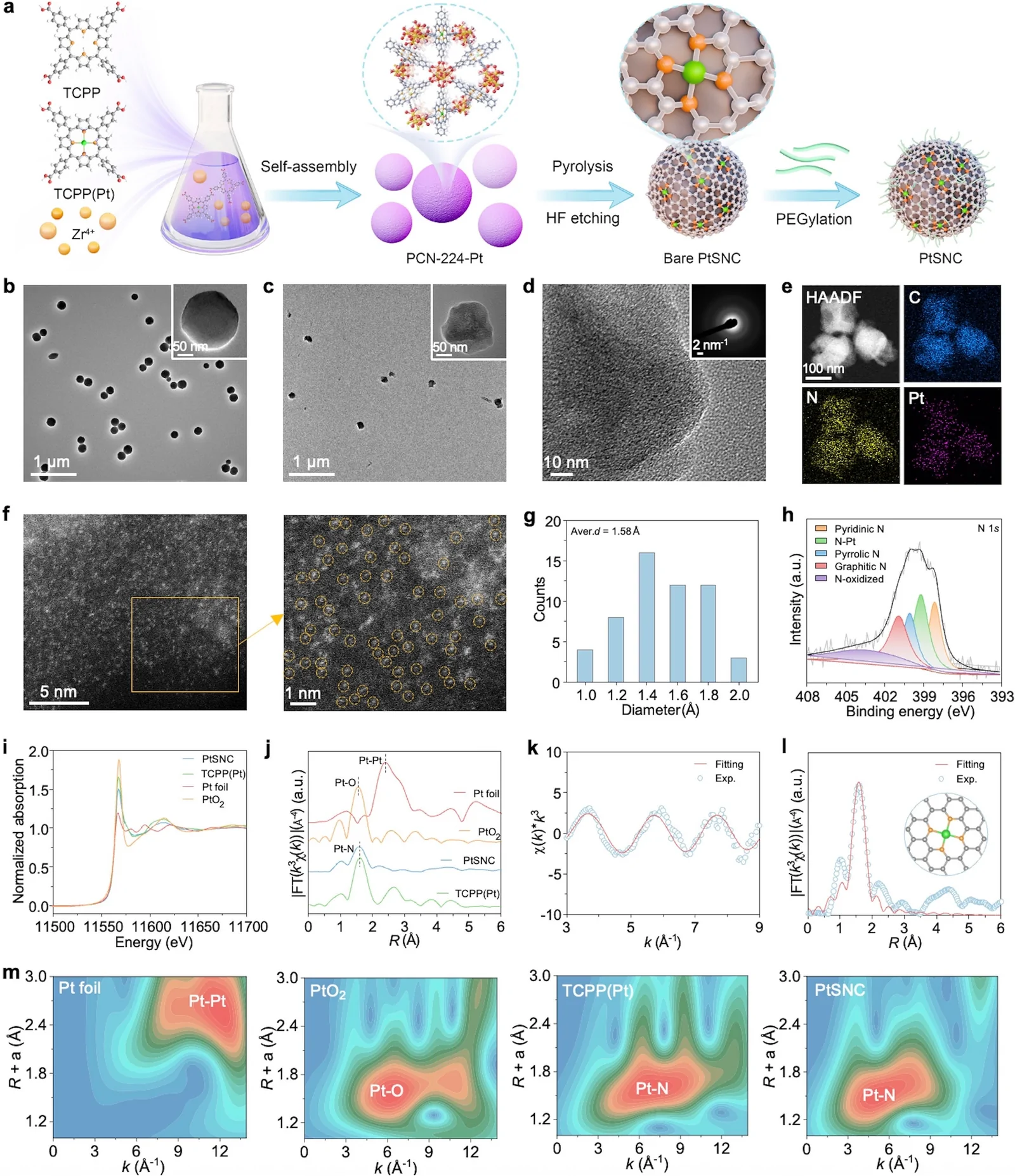
Synthesis and structural characterizations of PtSNC. **a** Schematic illustration of the synthesis process of PtSNC. TEM images of **b** PCN-224-Pt_20_ and **c** PtSNC. **d** HRTEM image and corresponding SAED pattern of PtSNC. **e** HAADF-STEM image and elemental mapping of PtSNC. **f** Atomic-resolution aberration-corrected HAADF-STEM image of PtSNC. The right image is an enlarged view of the yellow boxed area in the left image, with single Pt atoms marked by yellow circles. **g** Histogram diameter distribution of atomically dispersed Pt species. **h** N 1*s* XPS spectrum of PtSNC. **i** Normalized Pt L3-edge XANES spectra and **j** *k*^*3*^-weighed Fourier-transformed EXAFS spectra (in the* R*-spac**e** of Pt foil, PtO_2_, TCPP(P**t**, and PtSNC. **k** *k*-space and **l** *R*-space fitting results of PtSNC, with the simulated Pt-N_4_ coordination structure included as an inset (Pt: green; N: yellow; C: grey). **m** EXAFS wavelet transformation analysis for Pt foil, PtO_2_, TCPP(Pt), and PtSNC

Pt is recognized as a potential alternative to natural enzymes due to its high stability, low cytotoxicity, and multienzyme-like activities [33]. To achieve the controllable fabrication of PtSNC with high-density metal sites, a porphyrinic MOF (PCN-224, built by the coordination between zirconium (Zr) node and meso-tetra(4-carboxyphenyl) porphine (TCPP) ligand [37]) featuring adjustable nanoparticle size was selected as the N-rich precursor, and a mixed-ligand strategy involving TCPP and its metalized derivative TCPP(Pt) was employed to spatially immobilize Pt atoms within the 3D networks of MOF (denoted as PCN-224-Pt_x_, x%: weigh percentage of TCPP(Pt) in both ligands) (Fig. S1). Upon subsequent pyrolysis (800 °C) and acid etching, the optimized TCPP/TCPP(Pt)-assembled PCN-224-Pt_20_ was ultimately transformed into Pt-anchored N-doped carbon (i.e., PtSNC) (Fig. 2a).

Scanning electron microscopy (SEM) and transmission electron microscopy (TEM) images reveal that the PCN-224-Pt_x_ shows uniform spherical morphology (particle diameter ~ 190 nm), with no observable shape distortion upon increasing TCPP(Pt) incorporation (Figs. 2b and S2). X-ray diffraction (XRD) patterns confirm the preserved crystallinity of MOF variants (Fig. S3), corresponding to the typical PCN-224 structure [38]. During pyrolysis, the original crystalline structure collapsed, *in-situ* yielding ZrO_2_ phases due to the Zr_6_ cluster (Zr_6_O_4_(OH)_4_(H_2_O)_6_(OH)_6_(COO)_6_) of MOF structure (Fig. S4), while acid etching process efficaciously removed ZrO_2_ crystals (Fig. S5). Compared to PCN-224-Pt_20_ nanoparticle, PtSNC displays a relatively smaller size (~ 150 nm) after pyrolysis (Figs. 2c and S6b). High-resolution TEM (HRTEM) image and selected area electron diffraction (SAED) demonstrate the absence of detectable crystalline Pt particles on the amorphous carbon matrix (Fig. 2d), which is further validated by two broad shoulder peaks ((002) and (101)) characteristic of graphitic carbon with no metallic crystal signals in XRD pattern (Fig. S5) [39]. Aberration-corrected high-angle annular dark-field scanning TEM (HAADF-STEM) directly visualizes atomically dispersed single Pt atoms, with isolated bright spots (~ 1.58 Å in average diameter) that are smaller than the Pt atom covalent diameter (*d*_Pt_ = 2.6 Å) (Fig. 2f, g). Moreover, elemental mapping indicates homogeneous distributions of Pt and N elements across the whole carbon substrate (Figs. 2e and S5a, b). The high Pt loading (up to 4.15 wt%) in the PtSNC has been determined by inductively coupled plasma mass spectrometry (ICP-MS) (Table S1). The Raman spectra point out an intensity ratio of 0.89 between D-band (~ 1350 cm^−1^, defective carbon) and G-band (~ 1590 cm^−1^, graphitic carbon) for PtSNC, comparable to the Pt-free N-doped carbon derived from PCN-224 nanoparticle [40] (Fig. S7). Further, the high-resolution Pt 4*f* and N 1*s* spectra obtained from X-ray photoelectron spectroscopy (XPS) prove the existence of Pt^2+^ state and Pt-N_x_ coordination in the PtSNC, where the pyridinic N species (398.2 eV) could benefit the stabilization of single metal atoms conducive to catalytic activity [41] (Figs. 2h and S8).

Notably, treatment of PCN-224-Pt_40_ as the precursor led to Pt particle formation, evidenced by HRTEM and SAED (Fig. S9). This suggests that adjacent Pt atoms aggregated when the ligand-induced interatomic distance (d) fell below a critical threshold. By modulating the TCPP/TCPP(Pt) ratio in the MOF precursor, the spatial separation of the Pt-N_4_ sites of TCPP(Pt) within the 3D skeleton was precisely controlled (Fig. S1). Optimal ligand ratio (e.g., PCN-224-Pt_20_) prevented Pt aggregation during pyrolysis process, whereas higher TCPP(Pt) integration narrowed interatomic d, inevitably causing Pt particle formation. This MOF-based mixed-ligand strategy could efficiently minimize molecular stacking while maximizing metal atom dispersion, enabling effective high-density single-atom synthesis [39].

To elucidate the detailed atomic coordination structure of PtSNC, synchrotron radiation-based X-ray absorption near-edge structure (XANES) and extended X-ray absorption fine structure (EXAFS) analyses were performed. The Pt L3-edge XANES data verify the energy absorption threshold of PtSNC follows a trend of > Pt foil and < PtO_2_, and is closer to that of TCPP(Pt) (Fig. 2i), indicative of a Pt valence state between 0 and + 4 with a good inheritance from the TCPP(Pt) ligand. The corresponding Fourier-transformed EXAFS spectra exhibit a dominant peak at ~ 1.6 Å for PtSNC, attributed to the Pt–N bond, with no identifiable Pt–Pt signals in the 2–3 Å range (Fig. 2j), proving the existence of only single Pt atoms in the PtSNC [42]. Similar results can be found in the wavelet transformation analysis (Fig. 2m). Subsequently, the quantitative structural parameters collected by least-square fitting analysis unveil a coordination number of about 4 and a bond length of 2.01 Å, consistent with a Pt-N_4_ configuration matching the TCPP(Pt) ligand (Figs. 2k, l and S10, and Table S2). Collectively, these results validate the successful synthesis of PtSNC with abundant atomically dispersed Pt-N_4_ sites.

To enhance dispersion stability and biocompatibility, the as-pyrolyzed PtSNC was further PEGylated via hydrophobic interactions with DSPE-PEG_3400_-COOH [41] (Fig. 2a). The successful PEGylation was confirmed by the Fourier transform infrared (FTIR) spectra, the narrowed hydrodynamic size distribution, and the decreased zeta potential (Fig. S11a-c). The modification obviously improved the colloidal stability of PtSNC, as evidenced by prolonged dispersion persistence (Fig. S11d), ensuring suitability for biomedical applications. The PEGylated PtSNC was utilized in the following experiments.

### Multienzyme-Mimicking Catalytic Activities and Mechanism Study

**Fig. 3:**
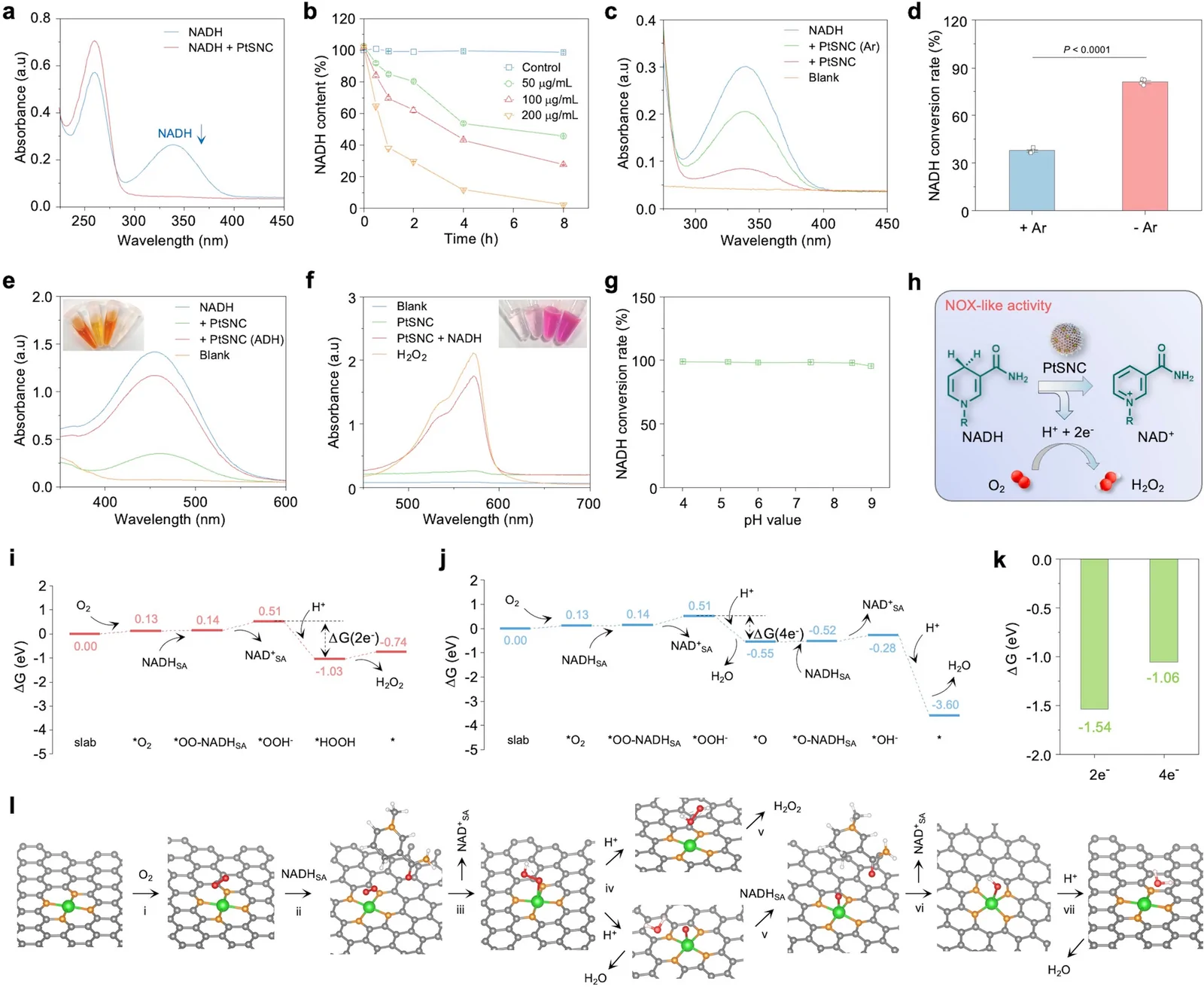
NOX-mimicking catalytic activity and mechanism of PtSNC. **a** UV–Vis absorption spectra of NADH before and after 8 h of catalytic reaction by PtSNC. **b** Time-dependent NADH depletion mediated by different concentrations of PtSNC. **c**, **d** Comparison of PtSNC-catalytic NADH depletion without and with removing dissolved oxygen using Ar purge. **e** Verification of the NAD^+^ generation during catalysis using alcohol dehydrogenase (ADH, which can selectively reduce NAD^+^ to NADH) and NADH-specific chromogenic reagent (absorbance at 450 nm). Inset: display of the test solutions after reactions. **f** Verification of the H_2_O_2_ generation during catalysis using a colorimetrical H_2_O_2_ assay kit (absorbance at 570 nm). Inset: display of the test solutions after reactions. **g** NOX-like catalytic activity of PtSNC at different pH conditions. **h** Schematic illustration of the PtSNC-mediated NOX-like catalysis process. **l** Proposed 2-electron and 4-electron pathways for NOX-like catalysis on the PtSNC model, and the free energy diagrams for **i** 2-electron and **j** 4-electron pathways determined by the DFT calculations. **k** Gibbs free energy variation of the watershed step in 2-electron or 4-electron pathway. Data are expressed as mean ± s.e.m. (*n* = 3 independent experiments in (**b**); *n* = 4 independent experiments in (**d**, **g**). Statistical significance was determined by two-tailed Student’s *t*-test (**d**)

NAD^+^/NADH redox pairs serve as a central metabolic cofactor, facilitating electron transfer in various metabolic redox reactions of cells [43]. NOX found in certain bacteria can directly convert NADH to NAD^+^, pointing out the potential for developing nanocatalysts with NOX-like activity to manipulate the NAD^+^/NADH levels [44, 45]. Noble metal catalysts (e.g., Pt, palladium (Pd)) possess excellent ability in catalyzing dehydrogenation reactions [46]. Therefore, the M–N-C nanomaterial with Pt-N_4_ coordination structure show potentiality as an effective nanocatalyst for activating the C-H bonds in NADH. To prove this, the NOX-like property and catalytic process of PtSNC were systematically investigated. Ultraviolet–visible (UV–Vis) spectroscopy reveals noticeable NADH depletion (characteristic absorbance at 340 nm) upon PtSNC treatment, suggesting its robust catalytic activity on oxidizing NADH (Fig. 3a). The NADH conversion rate increases with elevating PtSNC concentrations (Fig. 3b). This catalytic process exhibits oxygen (O_2_)-dependent and yields NAD^+^ and H_2_O_2_ as products (Fig. 3c-f). Although more acidic conditions accelerated the initial reaction rate, PtSNC achieved ~ 100% NADH conversion across a broad pH range (4–9) within 8 h (Figs. 3g and S12), highlighting its high efficiency and adaptability to diverse physiological environments. Based on the Pt-N_4_ single atom sites of PtSNC verified before, density functional theory (DFT) calculation was employed to elucidate the catalysis mechanism by using a simplified NADH analog (NADH_SA_) with a methyl (-CH_3_) group replacing the ribose-adenosine diphosphate (Ribo-ADP) group at the N site of NADH molecule (Fig. 3i, j, l). During the catalytic process, O_2_ adsorption on Pt sites forms *O_2_ intermediates, which abstracts one H^+^ and two electrons from NADH_SA_, generating NAD^+^_SA_ and *OOH^−^. Subsequent protonation enables production of H_2_O_2_ (2-electron pathway) or H_2_O (4-electron pathway). The comparable result for the Gibbs free energy variation of the watershed step of H_2_O_2_ (ΔG = − 1.54 eV) or H_2_O (ΔG = − 1.06 eV) production suggests the thermodynamical propensity for generating H_2_O_2_ via the 2-electron pathway [47], aligning with experimental observations (Fig. 3k). These results evidence PtSNC as a potent NOX mimic for catalytic conversion of NADH to NAD^+^ while generating H_2_O_2_ (Fig. 3h).

**Fig. 4:**
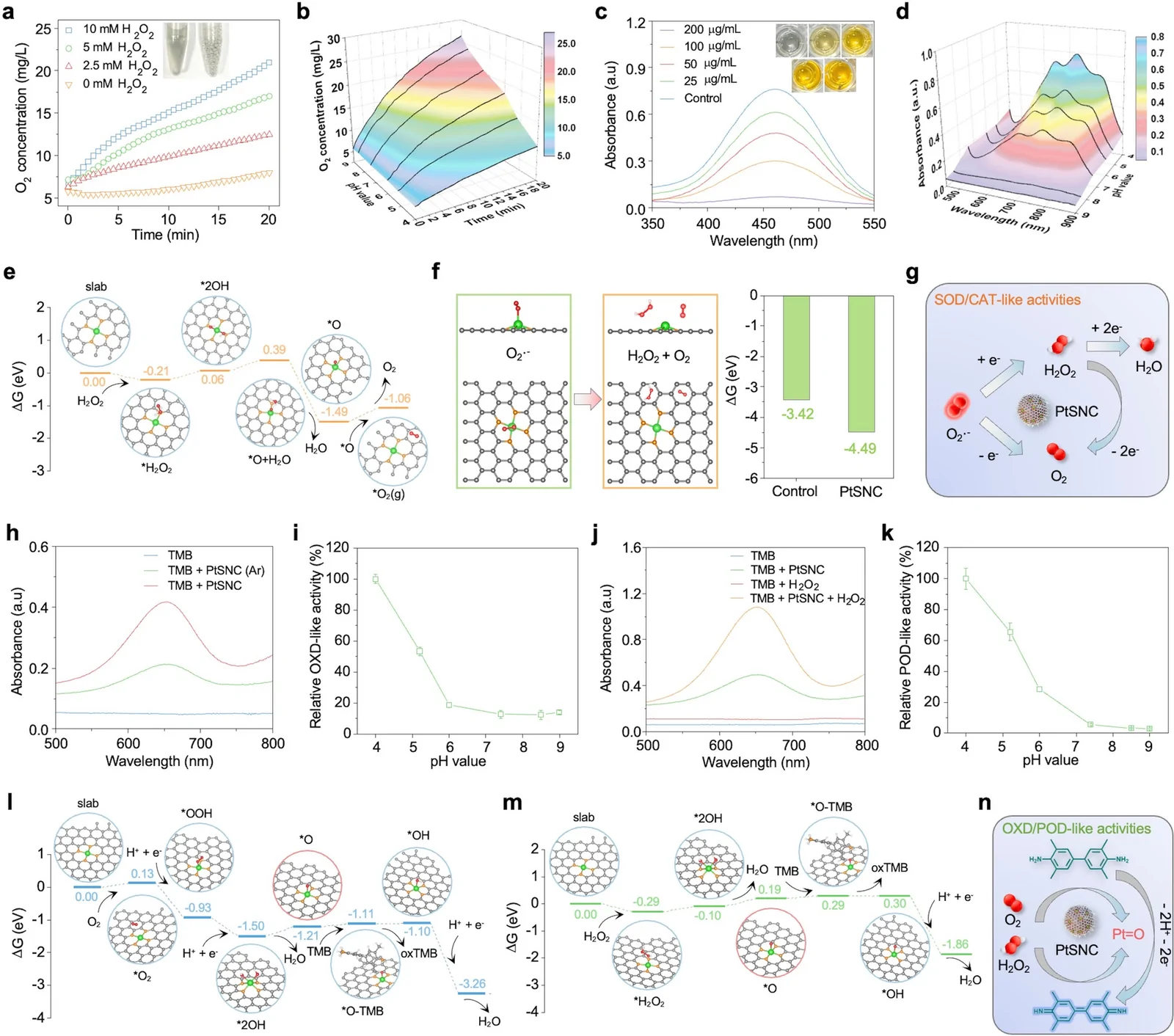
pH-responsive multienzyme-mimicking antioxidative/pro-oxidative activities and catalytic mechanisms of PtSNC. **a** H_2_O_2_ concentration-dependent and **b** pH-dependent CAT-like catalytic activity of PtSNC. Inset: display of the PtSNC-catalytic O_2_ generation. **c** Verification of SOD-like activity based on WST-8 method. Inset: display of the test solutions after reactions. **d** The total antioxidant capacity of PtSNC at different pH conditions tested by ABTS radical scavenging assay. **e** Proposed homolytic path for CAT-like catalysis and the DFT-determined free energy diagram. **f** Proposed O_2_·^−^ disproportionation process on the PtSNC model, and the Gibbs free energy variation of the O_2_·^−^ disproportionation assisted with or without PtSNC. **h** TMB oxidation by PtSNC without or with removing dissolved oxygen using Ar purge. **j** TMB oxidation by PtSNC without or with adding H_2_O_2_. **i** OXD-like or **k** POD-like catalytic activity of PtSNC at different pH conditions. Proposed catalytic pathways for **l** OXD-like and **m** POD-like catalysis, and the DFT-determined free energy diagrams. **g**, **n** Schematic illustrations of the PtSNC-mediated SOD/CAT-like cascade catalysis process and OXD/POD-like catalytic TMB oxidation process. Data are expressed as mean ± s.e.m. (*n* = 3 independent experiments in (**i**, **k**))

H_2_O_2_ and O_2_·^−^ as key ROS in organism are regulated by CAT and SOD. The Pt-N_4_ single atom sites in the synthetic PtSNC are similar to the metal catalytic centers of human erythrocyte CAT (PDB: 1DGF [48]) and mitochondrial SOD (PDB: 1N0J [49]), encouraging us to further explore the CAT/SOD-mimicking activities. PtSNC exhibits H_2_O_2_ concentration-/pH-dependent CAT-like catalytic activity, decomposing H_2_O_2_ to O_2_ with enhanced efficiency under neutral/alkaline conditions compared to acidity (Figs. 4a, b and S13a). Concurrently, its SOD-like catalytic activity was confirmed through the WST-8 assay, demonstrating O_2_·^−^ disproportionation into H_2_O_2_ and O_2_ (Figs. 4c and S13b). Then, the total antioxidant capacity test based on 2,2’-Azinobis-(3-ethylbenzthiazoline-6-sulphonate) (ABTS) reveals remarkable free radical scavenging in neutral/alkaline conditions but diminished activity under acidity (Figs. 4d and S13c). It indicates that the CAT/SOD-mimicking PtSNC can be used as an efficacious antioxidant under neutral/alkaline environments (Fig. 4 g). For CAT-like catalysis, two possible reaction pathways have been proposed by DFT calculation (Figs. 4e and S14a). Due to the far lower energy barrier for the homolytic path (*H_2_O_2_ → *2OH, ΔG = 0.28 eV) than that for the heterolytic path (*H_2_O_2_ → *OOH + H^+^  + e^−^, ΔG = 2.15 eV), the homolytic path is more kinetically favorable for H_2_O_2_ decomposition here (Fig. S14b). Moreover, the negative ΔG of the whole catalytic reaction based on the homolytic path suggests the thermodynamical feasibility (Fig. 4e). Additionally, DFT validates the Pt-N_4_ sites capable of facilitating the disproportionation of O_2_·^−^, consistent with SOD-like catalysis (Fig. 4f).

Subsequently, the OXD/POD-mimicking activities of PtSNC were assessed using 3,3′,5,5′-tetramethylbenzidine (TMB), which can be oxidized into blue oxTMB with a characteristic absorbance at 654 nm. PtSNC displays an excellent OXD-like catalytic activity on oxidation of TMB with the assistance of O_2_ (Figs. 4 h and S15). The TMB colorimetric reactions follow typical Michaelis–Menten kinetics at pH 5.2 (Michaelis–Menten constant (*K*_m_) = 1.40 mM, maximum reaction rate (*V*_max_) = 1.43 × 10^–7^ M s^−1^) (Fig. S15), hinting that PtSNC could not only easily absorb TMB substrate but also rapidly catalyze its oxidation. The OXD-like catalytic rate constant (*k*_cat_) was calculated to be 2.69 × 10^–2^ s^−1^, surpassing that of commercial Pt/C (1.01 × 10^–2^ s^−1^) and other typical OXD mimics (e.g., MnO_2_, Fe_3_O_4_, CeO_2_, etc.) [50]. Further, H_2_O_2_ addition activated the POD-like catalysis of PtSNC on oxidation of TMB (Figs. 4j and S16), following dual-substrate Michaelis–Menten kinetics yielding *K*_m_ = 6.89 mM (H_2_O_2_) and 1.22 mM (TMB) and *V*_max_ = 1.60 × 10^–7^ M s^−1^ (H_2_O_2_) and 5.92 × 10^–7^ M s^−1^ (TMB) (Fig S16). Its POD-like *k*_cat_ of 3.01 × 10^–2^ s^−1^ for H_2_O_2_ and 1.10 × 10^–1^ s^−1^ for TMB is higher than that of various POD mimics (e.g., Mn_3_O_4_, Fe_2_O_3_, CeO_2_, etc.) and even other M–N-C nanomaterials (e.g., Zn-N–C, Cu–N-C, Co–N-C, etc.) [51, 52], suggesting the prominent catalytic activity of PtSNC. Notably, both of catalytic activities can be enhanced with the increase of the reaction solution acidity, while almost no significant OXD/POD-like activities can be observed at neutral or alkaline condition (Fig. 4i, k).

Generally, nanocatalysts that mimic OXD or POD could probably catalyze the production of free ROS as active intermediates, e.g., O_2_·^−^, singlet oxygen (^1^O_2_), or hydroxyl radical (·OH) [26]. However, our targeted colorimetric experiments and electron spin resonance (ESR) measurements ruled out the involvement of O_2_·^−^, ^1^O_2_, and ·OH, yet potassium thiocyanate (KSCN) quenching attested Pt-N_4_ sites as the active centers (Figs. S17 and S18). Accordingly, we propose metal-oxo intermediate (i.e., Pt = O) as a plausible and essential active transient state in the OXD/POD-like catalysis of PtSNC [50, 53] (Fig. 4n). In situ diffuse reflectance infrared Fourier-transform spectroscopy (DRIFTS) measurements under O_2_ and H_2_O atmosphere provided supportive evidence for the formation of oxygen-related intermediates and a hydrated oxygen activation environment during catalysis (Fig. S19). The observation of a broad band in the range of 860–1210 cm^−1^, assignable to strongly bound Pt-O species associated with key Pt = O intermediates, corroborates the proposed non-radical reaction pathway. During the catalytic processes, O_2_/H_2_O_2_ activation enables formation of highly active Pt = O intermediates in PtSNC, driving TMB oxidation, also supported by DFT calculations (ΔG < 0 for the whole catalytic reactions) (Fig. 4 l, m). A protonated hydrogen atom is required to bind with *OH for simultaneous H_2_O release and Pt-N_4_ regeneration with substantially reduced Gibbs free energy, indicating that acidic environment favors such catalytic reactions, in accordance with experimental results. Thus, the combined evidence from in situ spectroscopic observations, the exclusion of conventional free ROS, active-site identification, and theoretical modeling collectively supports the Pt = O-mediated reaction pathway. GSH, an important antioxidant overexpressed in tumors [18], was further selected as another target substrate for catalytic validation. Ellman’s assay demonstrates PtSNC’s capacity to effectively catalyze the oxidation of GSH (Fig. S20). Moreover, DFT simulation reveals that the highly reactive Pt = O species are kinetically favorable for transforming GSH to its oxidized dimer (GSSG) (ΔG =  − 2.17 eV), underscoring the potential of PtSNC as a robust redox modulator (Fig. S21).

Collectively, PtSNC exhibits pH-tunable multi-catalytic capability: in neutral/alkaline conditions, concurrent CAT-, SOD-, and NOX-like catalytic cascades effectively modulate the NAD^+^/NADH levels, scavenge ROS (e.g., H_2_O_2_, O_2_·^−^), and replenish O_2_; while in acidic conditions, OXD-, POD-, and NOX-like catalytic parallel reactions generate plenty of highly reactive Pt = O species to propel oxidation reactions while changing the NAD^+^/NADH levels. Such discrepant environments-dependent catalytic duality make PtSNC a suitable nanoformulation to implement pathological contexts-specific metabolic interventions, presenting the potential for resolving the diabetic “pro-tumor, anti-healing” paradox.

### PtSNC-Enabled Bioenergetic Restoration in High Glucose (HG)-Impaired Cells

**Fig. 5:**
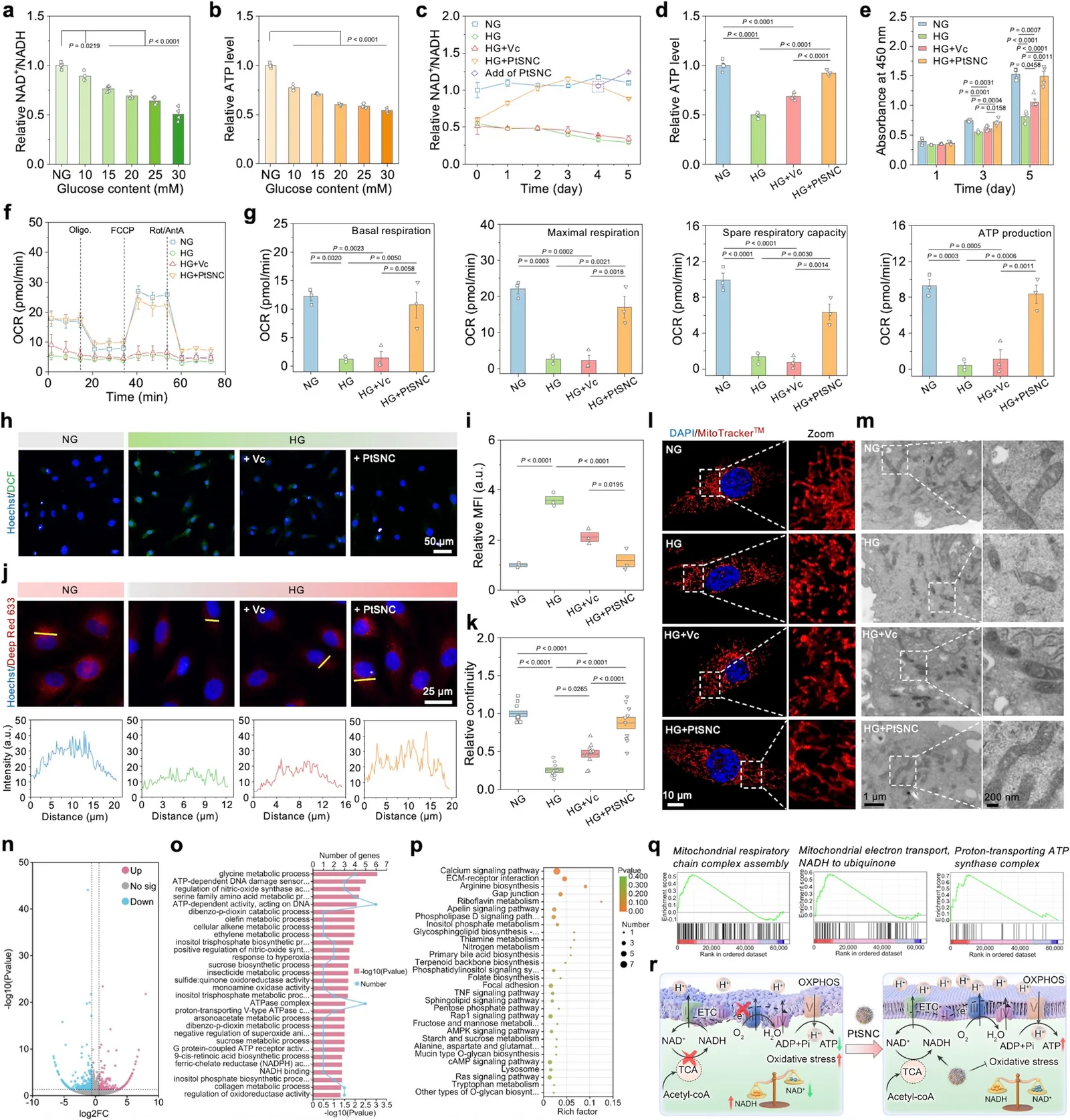
Reversing HG-induced bioenergetic disorder in endothelial cells by PtSNC for diabetic wound healing promotion. **a** The relative NAD^+^/NADH ratios and **b** ATP levels in the HUVECs incubated with different concentrations of glucose. **c** Time-dependent variation of the relative NAD^+^/NADH ratios in the HUVECs with different treatments. The purple dashed box hints the PtSNC replenishment conducted on day 4. **d** The relative ATP levels after different treatments on day 3. **e** Cell proliferation capacity analysis. **f** Cellular OCR measurement of HUVECs and **g** analysis of key respiratory capacity-related indexes. **h**, **i** DCFH-DA-based ROS detection fluorescence images and intensities. **j** Mitochondrial membrane potential analysis using Mito-Tracker Deep Red 633, and corresponding fluorescence intensities of the area marked by a yellow line. **l** Representative images of the treated HUVECs, where the mitochondria were stained with MitoTracker Red CMXRos for visualization, and **k** corresponding mitochondrial continuity statistics. **m** Representative bio-TEM images of mitochondria in the treated HUVECs. **n** Volcano map for the distribution of DEGs. **o** GO and **p** KEGG enrichment analyses of DEGs. **q** GSEA of bioenergy-related pathways between two groups. **r** Proposed PtSNC-mediated bioenergetic restoration mechanism in the HG-impaired cells. Data are expressed as mean ± s.e.m. (*n* = 3 independent experiments in (**f**, **g**, **i**; *n* = 4 independent experiments in (**a**-**e**); *n* = 10 independent experiments in (**k**). Statistical significance was determined by one-way ANOVA with Tukey’s *post-hoc* test (**a, b, d, e, g, i, k**)

To assess the capacity of PtSNC to restore bioenergetic homeostasis in normal cells under hyperglycemic stress, we first examined its effect in HG-impaired models in vitro. In healthy cells, the NAD^+^/NADH redox pairs sustain metabolic homeostasis to ensure normal energy supply, biological process, and signal transduction. However, hyperglycemia disrupts this equilibrium with triggering NADH accumulation and NAD^+^ depletion, leading to mitochondrial dysfunction and oxidative stress in specific cells—an important pathological cascade underlying chronic wound healing in diabetics [16, 17, 54]. Accordingly, restoring NAD^+^/NADH homeostasis via nanomaterial intervention holds great therapeutic promise, while such strategy remains largely unexplored. In view of the distinguished multienzyme-mimicking (i.e., cascade NOX, CAT, and SOD-like catalysis) capability of PtSNC in neutral conditions, we assessed its therapeutic potential to renovate hyperglycemia-induced bioenergetic disorder. In different cell lines including human umbilical vein endothelial cells (HUVECs), immortal keratinocytes (HaCaTs), and dermal fibroblasts (HDFs), PtSNC dose-dependently elevated intracellular NAD^+^/NADH ratios without affecting their viabilities, indicative of its potential to modulate cell metabolism (Figs. S22 and S23). Subsequently, the bioenergetic disorder model was built in HUVECs by treating high glucose (HG, 25.0 mM) to induce mitochondrial dysfunction [55, 56]. Notably, escalating concentrations of glucose treatment progressively lowered the NAD^+^/NADH ratios in cells, paralleled by diminished ATP levels and moderate reductions in cell viability (Figs. 5a, b and S24). Besides, compared to the normal glucose (NG, 5.5 mM) culture condition, the HG exposure induced pronounced oxidative stress and marked mitochondrial membrane depolarization (Fig. 5h-j). These results validate the successful establishment of a HG-induced bioenergy-dysfunctional cell model in vitro.

To distinguish the roles of catalytic convention of NADH to NAD^+^ and anti-ROS catalysis, vitamin C (Vc), a potent antioxidant lacking NOX-like capacity, was selected as the contrastive agent (Fig. S25). The intracellular ROS detection assay based on 2,7-dichlorofluorescein diacetate (DCFH-DA) indicator reveals that in the HG-impaired cells, Vc significantly reduces oxidative stress whereas PtSNC exhibits superior ROS-scavenging efficacy owing to its excellent CAT/SOD-like catalytic activities (Fig. 5h, i). Furthermore, in the hypoxia assessment employing luminescent oxygen sensor [Ru(dpp)_3_]Cl_2_, PtSNC largely alleviates cellular hypoxia compared to the untreated and Vc-treated groups (Fig. S26), attributing to its catalytic O_2_ generation. As shown in Fig. 5c, treatment of HG-impaired cells with PtSNC sustainably elevates the ratio of NAD^+^/NADH, peaking near-normal levels by day 3, and then undergoes gradual decline probably due to normal cell metabolism and the persistent HG stress, whereas Vc treatment exerts no discernible effect. Moreover, replenishment of fresh PtSNC can re-boost the NAD^+^/NADH ratio. This attributes the NAD^+^ elevation to the NOX-like catalytic capacity of PtSNC, and underscores that the modulation is a catalytically driven, reversible process. As anticipated, PtSNC can rescue ATP production to near-normal levels in the HG-impaired cells, while Vc fails to largely restore ATP levels (Fig. 5d), underscoring that mere antioxidant effect cannot rectify bioenergetic disorder and manipulating the NAD^+^/NADH levels is pivotal for energy metabolic recovery. To further establish whether this bioenergetic restoration is causally dependent on the PtSNC‑mediated NAD^+^/NADH reprogramming, function-blocking experiments were performed using the specific nicotinamide phosphoribosyltransferase (NAMPT) inhibitor FK866 (Fig. S27). Compared with PtSNC treatment alone, co‑treatment with FK866 significantly blunted the recovery of cellular ATP levels and impaired the PtSNC‑induced upregulation of key energy metabolism-related proteins, including AMPK, IDH1, and OGDH [57]. These results provide direct causal evidence that the metabolic benefits of PtSNC are dependent on its capacity to elevate NAD^+^ levels and sustain NAD^+^/NADH homeostasis. Disturbing this homeostatic maintenance pharmacologically severs the causal link between PtSNC catalysis and metabolic recovery.

To deeply investigate the influence of PtSNC on HG-induced mitochondrial dysfunction, the respiratory functions, oxidative metabolic state, and morphology of mitochondria were further evaluated. The oxygen consumption rate (OCR) analysis shows that HG obviously impairs OXPHOS process while PtSNC treatment robustly restores mitochondrial functions, evidenced by the upregulation of key respiratory capacity-related indexes (Fig. 5f, g). Meanwhile, PtSNC substantially normalizes mitochondrial redox status (Fig. 5j). In contrast, Vc treatment exhibits negligible modulation effects on damaged respiration functions, and marginally improves depolarized mitochondrial membrane potential in the HG-impaired cells (Fig. 5f, g, j). Clearly, HG-induced mitochondrial fragmentation and discontinuity in cells can be reversed by PtSNC treatment, which restores continuous and intact mitochondrial networks similar to control cells (Fig. 5k, l). TEM images display that PtSNC rescues the ultrastructural defects of mitochondria, including cristae disorganization and membrane blurring caused by HG (Fig. 5m). In addition, the cell functional tests demonstrate that PtSNC, but not Vc, fully improves the proliferation and migration capacities of HG-impaired cells, which are critical processes for tissue regeneration and angiogenesis (Figs. 5e, S28, and S29). Together, these data jointly indicate that PtSNC, harnessing its cascade NOX-/CAT-/SOD-like activities to catalyze NAD^+^-elevating, ROS-scavenging, and O_2_-evolving under neutral environments, can synchronously rectify NAD^+^/NADH homeostasis, mitochondrial functions and oxidative metabolic state, thereby reversing HG-induced bioenergetic chaos and reinstating cellular vitality.

Then, RNA-sequencing transcriptomic analysis was performed to elucidate the potential therapeutic mechanisms of PtSNC on the impaired HUVECs (Fig. S30). As a result, 613 differentially expressed genes (DEGs) were identified, including 239 upregulated and 374 downregulated in the PtSNC-treated cells compared to the control group (Figs. 5n and S30). Gene Ontology (GO) annotation and enrichment analyses link these DEGs to multiple aspects of biological processes, cellular components, and molecular functions, revealing pronounced correlations with amino acid/alkene metabolic processes, biosynthesis, oxidoreductases, and ATP-associated activities (Figs. 5o and S31). Kyoto Encyclopedia of Genes and Genomes (KEGG) enrichment analysis further highlights that plenty of metabolic/biosynthetic processes are significantly affected, involving Arginine biosynthesis, Thiamine metabolism, Nitrogen metabolism, Primary bile acid biosynthesis, Starch and sucrose metabolism, Alanine, aspartate and glutamate metabolism, etc. (Fig. 5p). Several important signaling pathways related to mitochondrial functions are obviously modulated, including Calcium signaling pathway, AMPK signaling pathway, cAMP signaling pathway, etc. [58–60] (Fig. 5p). Additionally, PtSNC treatment also affects ECM-receptor interaction, Gap junction, Focal adhesion, Apelin signaling pathway, and Rap1 signaling pathway, which are closely associated with cell proliferation, migration, adhesion, and angiogenesis activities [61, 62]. Gene Set Enrichment Analysis (GSEA) demonstrates significant upregulation of mitochondrial OXPHOS, ETC, ATPase activity, and oxidoreductase complex (Figs. 5q and S32), aligning with mitochondrial functional improvement observed in the above cellular assays. In summary, we propose that PtSNC acts as a potent metabolic regulator, reprogramming energy metabolism and redox homeostasis by rescuing HG-induced mitochondrial impairment, which can effectively improve cellular functionalities and behaviors benefiting for diabetic wound healing (Fig. 5r).

### Bioenergetic Disturbance in Tumor Cells Induced by PtSNC

**Fig. 6:**
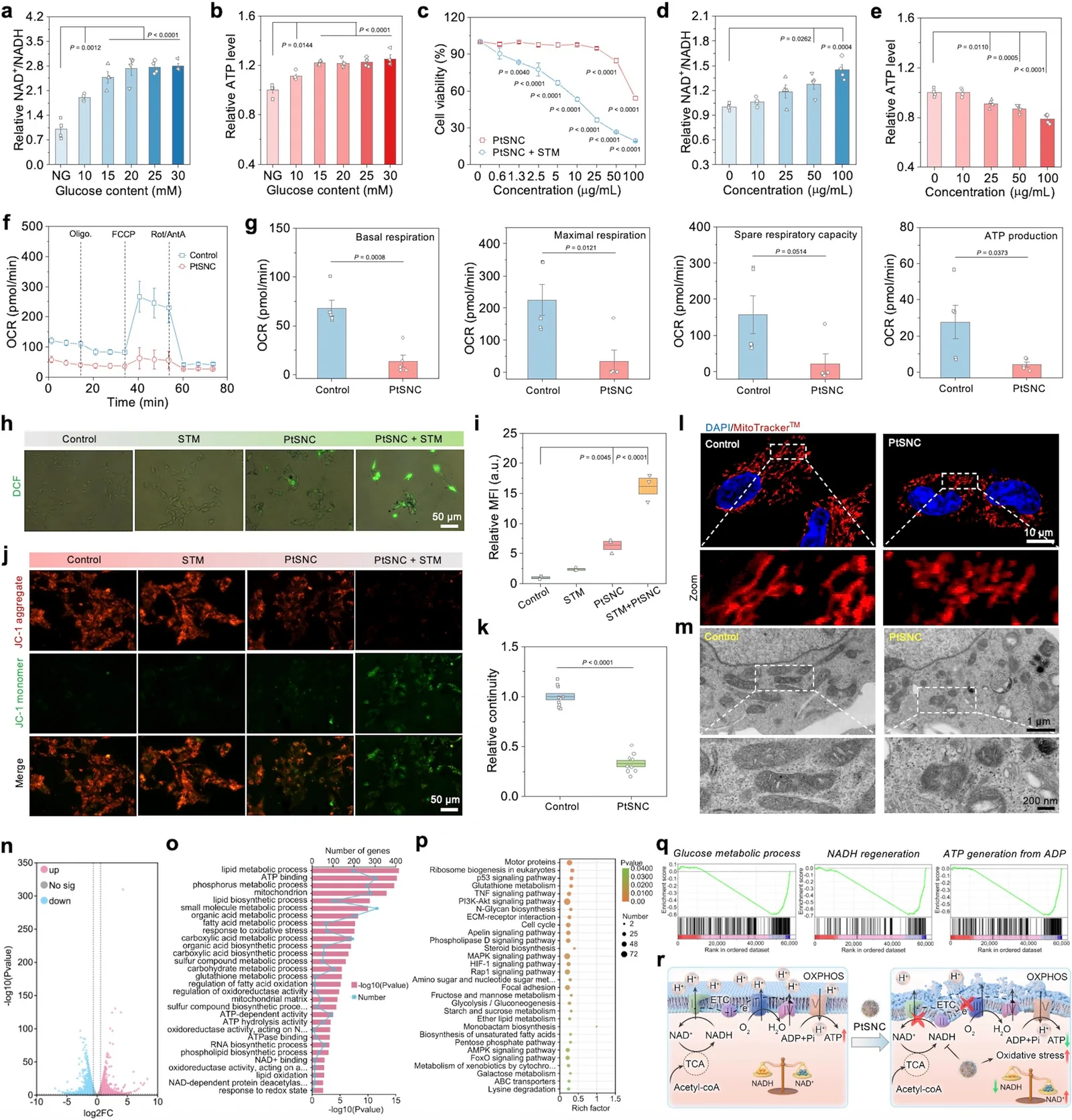
Inducing bioenergetic disturbance in tumor cells by PtSNC for cell exhaustion and death. **a**, **d** The relative NAD^+^/NADH ratios and **b**, **e** ATP levels in the B16F10 cells after incubation with different concentrations of glucose or PtSNC. **c** Cell viability of the B16F10 cells incubated with different concentrations of PtSNC under non-STM or STM culture condition. **f** Cellular OCR measurement of B16F10 cells and **g** analysis of key respiratory capacity-related indexes. **h**, **i** DCFH-DA-based ROS detection fluorescence images and intensities. **j** Fluorescence images of JC-1 staining of B16F10 cells. **l** Representative images of B16F10 cells with the mitochondria stained with MitoTracker Red CMXRos, and **k** corresponding mitochondrial continuity statistics. **m** Representative bio-TEM images of mitochondria in the treated B16F10 cells. **n** Volcano map for the distribution of DEGs. **o** GO and **p** KEGG enrichment analyses of DEGs. **q** GSEA of bioenergy-related pathways between two groups. **r** Proposed PtSNC-mediated bioenergetic disturbance mechanism in the B16F10 cells. Data are expressed as mean ± s.e.m. (*n* = 3 independent experiments in (**i**); *n* = 4 independent experiments in (**a**-**e**); *n* = 5 independent experiments in ( (**f**,**g**); *n* = 10 independent experiments in (**k**). Statistical significance was determined by one-way ANOVA with Tukey’s *post-hoc* test (**a-e**, **i**) or two-tailed Student’s *t*-test (**g**, **k**)

We next evaluated whether PtSNC could disrupt energy metabolism and induce cell death in tumor cells, even under glucose-replete conditions that favor tumor progression. Different from normal cells, tumor cells exhibit highly elevated glucose uptake and accelerated glycolysis even under normoxic environments, while maintaining almost unaltered respiration (the “Warburg effect”) [63]. In tumor cells, lactate dehydrogenase isoform A (LDHA) preferentially transforms accumulating pyruvate to lactate, with reproducing NAD^+^ from NADH to sustain glycolysis [36]. This process fosters an acidic TME through excessive lactate secretion, promoting tumor progression. Additionally, the “lactate shuttle” phenomenon enables it re-entry into certain tumor cells to fuel the TCA cycle and OXPHOS [64]. According to the LDHA reaction, the lactate accumulation hints a lowered NAD^+^/NADH ratio: [Pyruvate]/[Lactate] ∝ [NAD^+^]/[NADH] [36, 65, 66]. Hence, hyperglycemia can enhance tumor bioenergetics and progression [19]. From this perspective, depleting NADH reserves to disrupt NAD^+^/NADH homeostasis can inhibit both glycolysis and OXPHOS to cause energy privation for therapeutic benefit. Encouraged by the excellent NOX-, OXD-, and POD-like catalytic activities of as-designed PtSNC in acidic conditions, we then investigated the in vitro anti-tumor capability utilizing highly malignant B16F10 melanoma cells as a tumor cell model. Firstly, the response of tumor cells to varying concentrations of glucose in the culture environment was tested, revealing that the cell viability decreased at the glucose levels below 15 mM but plateaued above this threshold (Fig. S33). This trend paralleled the reductions in intracellular NAD^+^/NADH ratios and ATP levels (Fig. 6a, b), which could be explained by insufficient glucose supply impairing NAD^+^ regeneration and subsequently perturbing energy metabolism. The findings indicate that elevated glucose environments favor tumor cell survival and proliferation by stabilizing NAD^+^/NADH homeostasis and meeting exacerbated bioenergetic demands—a phenomenon diametrically opposed to the HG adaptation of normal cells (Figs. 5a, b and S24). This contrast creates a so-called “metabolic bifurcation”. Accordingly, we further examined the intervention effects of microenvironments-adaptive PtSNC on bioenergetically enriched tumor cells (HG environments).

Notably, PtSNC dose-dependently reduced tumor cell viability, with enhanced cytotoxicity under the culture conditions that simulated the TME (STM, pH 6.0 + H_2_O_2_ 100 µM) due to the TME-tendentious multi-catalytic therapeutic functions of PtSNC (Fig. 6c). PtSNC not only lowered the proportion of NADH to disrupt NAD^+^/NADH homeostasis, but also induced cellular oxidative stress which was exacerbated under STM conditions (Fig. 6d, h, i). These results further suggest the achievement of its NOX-/OXD-/POD-like catalysis for oxidation of NADH and generation of highly reactive Pt = O species within tumor cells. Specifically, treatment of PtSNC can cause significant mitochondrial damage, evidenced by mitochondrial respiratory dysfunction (Fig. 6f, g), membrane potential depolarization (Figs. 6j and S34), morphology fragmentation (Fig. 6k, l), and structural disorganization (Fig. 6m), culminating in ATP depletion for bioenergetic disturbance (Fig. 6e). To validate whether the anti‑tumor bioenergetic collapse is causally driven by its NOX-like catalysis, we conducted function‑rescuing experiments by supplementing the NAD^+^ precursor nicotinamide mononucleotide (NMN). NMN, as a pure metabolic substrate without direct antioxidant activity, can expand the total NAD(H) pool and enhance the metabolic buffering capacity against PtSNC’s catalytic NADH depletion. Notably, co‑treatment with NMN rescued the PtSNC‑induced ATP decline to some extent and partially reversed the downregulation of pro‑survival signaling proteins (PI3K and AKT) and key energy metabolic enzymes (IDH1 and OGDH) (Fig. S35). These findings establish a causal link between PtSNC’s NOX‑like catalysis and the subsequent collapse of both energy metabolism and PI3K/AKT‑driven metabolic/survival signaling [67, 68], culminating in irreversible tumor cell energy depletion and death. This incomplete rescue, however, indicates that while NAD^+^/NADH homeostasis disturbance is a critical initiating trigger, the concurrent POD-/OXD‑like oxidative catalysis constitutes an indispensable synergistic mechanism that amplifies and executes the final tumor-inhibiting signal. In addition, the tumor cell death pathways induced by PtSNC was also examined. The GSH detection assay confirms that PtSNC is able to remarkably consume intracellular GSH (Fig. S36), which could be attributed to two points: 1) the highly reactive Pt = O species generated by PtSNC can oxidize GSH, as demonstrated in the catalytic performance tests, and 2) bioenergetic intervention may impair the antioxidant defenses of tumor cells, involving GSH expression [18]. Breaking tumor energy metabolism and redox homeostasis is widely recognized to induce cell death by activating ferroptosis and/or apoptosis [26, 69, 70]. Indeed, PtSNC treatment significantly downregulated the expression of glutathione peroxidase 4 (GPX4) and caused irreversible lipid peroxidation (LPO) within tumor cells—hallmarks of ferroptotic pathway [71] (Figs. S37 and S38). Concomitantly, PtSNC activated the cysteine-requiring aspartate protease-3 (Caspase-3), indicative of apoptotic pathway induction [72] (Fig. S39). Additional function-rescuing experiments using specific death inhibitors further confirmed the major contribution of these two pathways: co-treatment with ferrostatin‑1 (a ferroptosis inhibitor) or Z‑VAD‑FMK (an apoptosis inhibitor) significantly rescued cell viability, whereas necrostatin‑1 (a necroptosis inhibitor) showed no protective effect (Fig. S41). The potent rescue by the antioxidant *N*-acetyl-L-cysteine (NAC) further underscored the strong relevance of oxidative damage to PtSNC’s tumor-inhibiting effect (Fig. S41). The dual cell death pathway was notably exacerbated under STM conditions, culminating in massive tumor cell mortality (Figs. S38-S40). Collectively, PtSNC exploits its TME-selective NOX-/POD-/OXD-like catalytic activities to deplete NADH reserves, destabilize redox homeostasis, and cripple energy metabolism even under glucose-sufficient conditions, while synergistically inducing ferroptosis and apoptosis to eradicate tumor cells.

RNA-sequencing transcriptomic profiling of PtSNC-treated melanoma cells identified 4090 DEGs, of which 1720 were upregulated and 2370 were downregulated compared to the control group (Figs. 6n and S42). GO annotation and enrichment analyses implicate these DEGs in various metabolic/biosynthetic processes, redox responses and regulations, mitochondrial matrix, and ATP/NAD-dependent activities (Figs. 6o and S43). KEGG enrichment analysis also emphasizes the markable impact on metabolic/biosynthetic processes, including Glutathione metabolism, Steroid biosynthesis, Amino sugar and nucleotide sugar metabolism, Fructose and mannose metabolism, Ether lipid metabolism, Biosynthesis of unsaturated fatty acids, Lysine degradation, etc. (Fig. 6p). Thereinto, Glutathione metabolism intervention is closely associated with the GSH depletion and GPX4 downregulation mentioned above [73–75]. PtSNC treatment largely regulates the P53, TNF, PI3K/AKT, MAPK, HIF-1, AMPK, FoxO signaling pathways and Glycolysis/Gluconeogenesis, all of which are closely related to cellular energy metabolism, redox homeostasis, and death [76, 77] (Fig. 6p). Furthermore, GSEA testifies the inhibitory effects of PtSNC on glycolytic and glucose catabolic processes, NADH regeneration and metabolic processes, oxidoreductase activity, and ATP generation from ADP (Figs. 6q and S44). Therefore, PtSNC holds promise as an efficacious tumor-specific bioenergetic disruptor to inhibit biogenesis and induce death of tumor cells for treating metabolically resilient tumors (Fig. 6r).

### In Vivo Diabetic Wound Healing-Promoting Effect

**Fig. 7:**
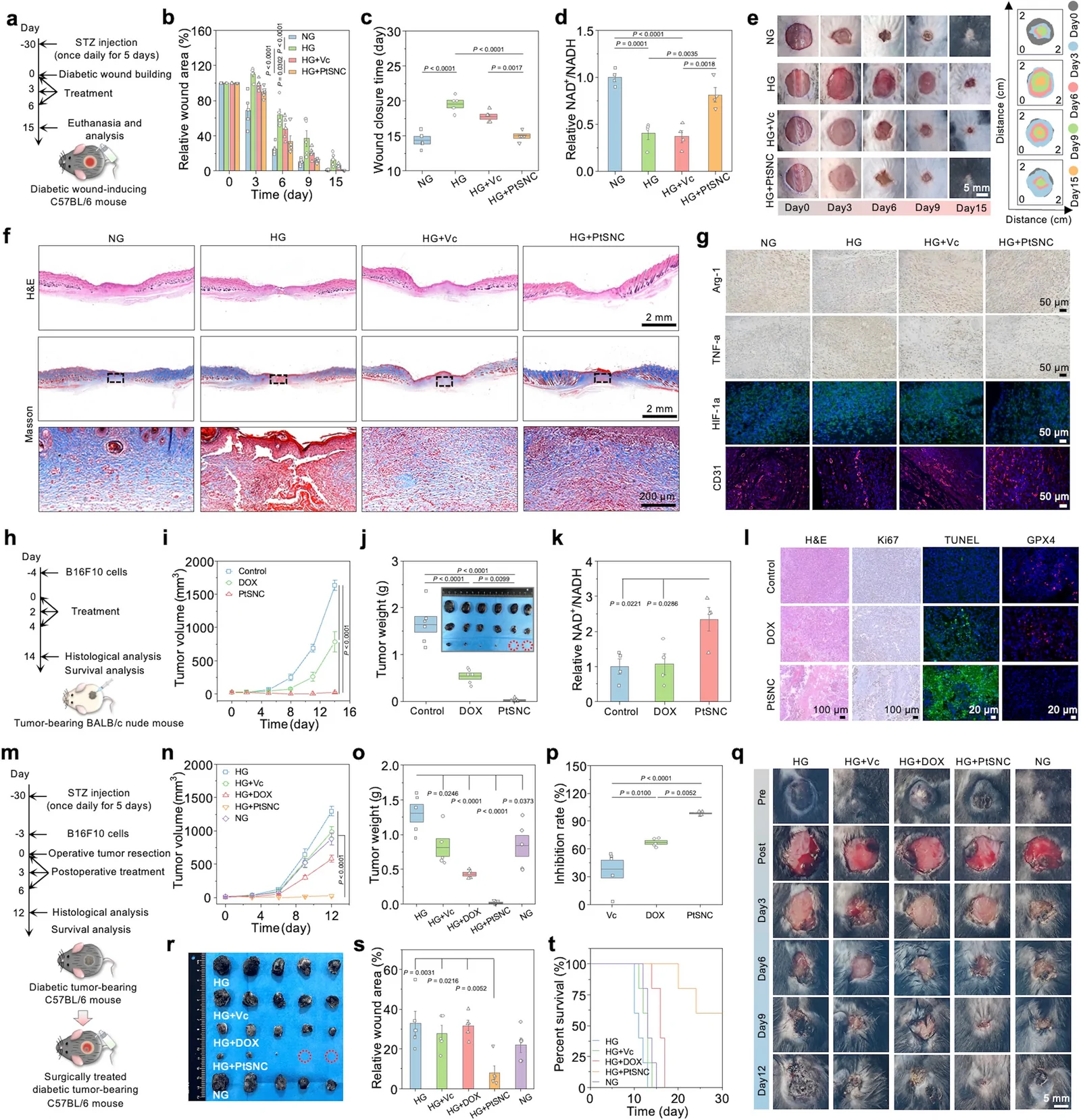
In vivo therapeutic assessments for the concurrent diabetic wound healing and tumor regression. Schematic illustrations of experimental protocols for **a** wound healing on diabetic skin wound-inducing mice, **h** tumor therapy on orthotopic melanoma-bearing mice, and **m** combined anti-tumor/wound healing on diabetic melanoma resection mice. **b** Quantitative analysis of the wound areas, **c** wound closure times, and **e** photographs of skin wounds and traces of unhealed wounds in different treated groups. **d** The relative NAD^+^/NADH ratios in the newly formed skin tissues in the wounds harvested at day 3. **f** H&E/Masson staining images of the wound skin tissues harvested at day 15. The bottom images are the enlarged view of the black boxed area in the Masson staining images. **g** Arg-1/TNF-α immunohistochemistry (at day 6), HIF-1α (at day 6) and CD31 (at day 15) immunofluorescence analyses of the wound skin tissues. **i** Tumor growth curves during different treatments. **j** Weights of the tumors extracted from different treated groups. Inset: display of the extracted tumors. The red dashed circle indicates the complete tumor elimination after treatments. **k** The relative NAD^+^/NADH ratios in the tumors at 48 h post-different treatments. **l** H&E/Ki67/TUNEL staining and GPX4 immunofluorescence analyses of tumor tissues. **n**, **q** Tumor recurrence monitoring post-operative resection during different treatments. **o** Weights and **r** photographs of the tumors extracted from different treated groups. The red dashed circle indicates the complete tumor recurrence suppression after treatments. **p** Calculated tumor inhibition rates for different groups. **s** Quantitative analysis of the wound areas at day 12. **t** Survival curves of the mice in different groups. Data are expressed as mean ± s.e.m. (*n* = 4 independent experiments in (**d**, **k**); *n* = 5 independent experiments in (**b, c, n-p, r**-**t**); *n* = 6 independent experiments in (**i, j**). Statistical significance was determined by one-way ANOVA with Tukey’s *post-hoc* test (**c, d, j, k, o, p, s**) or two-way ANOVA with Bonferroni’s *post-hoc* test (**b, i, n**)

Building on the pathological contexts-selective multi-catalytic activities of PtSNC and its bioenergetic restoration in HG-impaired endothelial cells in vitro, we next investigated its therapeutic potential in diabetic chronic wound healing. Full-thickness circular dorsal skin wounds were created on the diabetic C57BL/6 mice (HG group, induced by streptozotocin (STZ)), with the non-diabetic mice as healthy controls (NG group) (Fig. 7a). These untreated wounds received phosphate-buffered saline (PBS) while the treatment groups were administered with Vc (HG + Vc group) or PtSNC (HG + PtSNC group) via spray followed by occlusive dressing (Fig. S45). According to the results of wound area variation and closure time statistics, the HG group exhibited delayed wound healing compared to the NG group (Fig. 7b, c, e). Treatment with Vc partially accelerated wound healing, while PtSNC treatment showed a more efficient promotion and achieved complete closure on day 15, matching the healing rate of healthy controls (Fig. 7b, c, e). In contrast with the NG group, the HG group has higher blood glucose levels and lower body weight (Fig. S46), which are the typical pathological features of diabetes. During the treatment period, PtSNC treatment did not obviously alter blood glucose levels, body weight (Fig. S46), blood-related parameters (Fig. S47), liver/kidney function indexes (Fig. S48), and histologic characteristics of major organs (Fig. S49), suggesting the high biosafety of PtSNC.

The histological analyses involving H&E and Masson staining were performed to explore the healing status within the skin tissues. As shown in the staining images (Figs. 7f and S50), the PtSNC-treated diabetic wounds developed robust scab formation by day 6 and regenerated intact neoepidermis with nascent hair follicles and dense collagen deposition by day 15, akin to the non-diabetic wound healing. In contrast, the untreated diabetic wounds showed minimal scabbing by day 6, delayed re-epithelialization and sparse collagen generation by day 15 (Figs. 7f and S50). In addition, the HG + Vc group improved epidermal closure, but failed to restore subcutaneous collagen fiber density compared to the HG + PtSNC group on day 15, implying the incomplete skin reconstruction and immature tissue structure with single antioxidant treatment. These results reveal that PtSNC with multifaceted modulating functions could efficiently facilitate wound repair in vivo by promoting both neoepidermis generation and collagen deposition.

Further experiments were carried out to reveal the latent mechanisms of boosted wound healing in vivo by PtSNC. Compared with the NG group, the HG group maintained relatively low levels of NAD^+^/NADH and ATP in the wound tissues (Figs. 7d, S51, and S52), manifesting the bioenergetic disorder induced by hyperglycemia that impedes wound healing. In the early healing process (day 3, 6, and 9), PtSNC treatment transiently restored the NAD^+^/NADH ratios and ATP levels in the diabetic wounds close to the non-diabetic levels, whereas Vc treatment showed negligible effect (Figs. 7d, S51, and S52). This demonstrates that PtSNC, but not Vc, can exert NOX-like catalytic activity in the wound environments to achieve catalytic NAD^+^ regeneration and thereby restoration of energy metabolism. By day 15, however, the NAD^+^/NADH ratios and ATP levels in the PtSNC-treated diabetic wounds declined to the untreated diabetic levels, likely due to that PtSNC shows metabolizable and non-persistent nature and the newly formed tissues/cells post-treatment are in the persistent hyperglycemic environments of diabetes. The dihydroethidium (DHE) staining of diabetic wound sections proves the effective catalytic ROS-scavenging ability of PtSNC in neutral tissue environments (Fig. S53). The results of immunohistochemical staining confirm the reduced anti-inflammatory Arginase-1 (Arg-1) and increased pro-inflammatory tumor necrosis factor-α (TNF-α) expressions in the HG group compared to the NG group (Fig. 7g), attributable to the ROS overproduction in the diabetic wounds to cause detrimental inflammation [78, 79]. PtSNC treatment elevated Arg-1 and suppressed TNF-α more effectively than Vc treatment (Fig. 7g), indicating the synergistic effects of energy metabolism and redox reprogramming on eliminating inflammation. O_2_ supply in the chronic wounds is beneficial for increasing cell activity and promoting angiogenesis [80]. Excitingly, the hypoxia-inducible factor 1α (HIF-1α) expression was significantly downregulated and the final CD31-positive vascular density was largely enhanced in the HG + PtSNC group (Fig. 7g), demonstrating the capacities of PtSNC to catalyze the oxygenation and promote the vascularization in vivo. Thus, PtSNC can drive bioenergetic normalization in the early healing stage to enhance cellular functionalities, and collaboratively promote diabetic chronic wound repair through simultaneously relieving inflammation, alleviating hypoxia, and accelerating angiogenesis.

### In Vivo Anti-Tumor Performance

Inspired by the bioenergetic disturbance and killing effects of PtSNC on tumor cells in vitro, its in vivo anti-tumor efficacy was further assessed using a B16F10 melanoma-bearing mouse model (Fig. 7h). These tumor-bearing mice were randomized into 3 groups with different interventional treatments: 1) control group; 2) doxorubicin (DOX, a clinically used chemotherapeutic drug) group; and 3) PtSNC group. During a 14-day treatment period, the PtSNC group exhibited significant tumor growth suppression compared to the control group. In contrast, treatment with DOX at a clinically relevant dose showed only moderate tumor-inhibiting efficacy, probably due to drug resistance (Fig. 7i). Final tumor excisions reveal striking therapeutic divergence, with near-complete tumor eradication in several PtSNC-treated mice (Figs. 7j and S54d). The tumor-inhibiting rates, calculated from the weight of the extracted tumors, are 98.5% for the PtSNC group and 67.2% for the DOX group (Fig. S54b). This indicates the superior in vivo potency of PtSNC with multiple regulatory capabilities. Furthermore, PtSNC significantly extended the survival time of the tumor-bearing mice that had received the treatment (Fig. S54c). Fortunately, no remarkable body weight loss or histopathological lesion on major organs was observed in the PtSNC group compared to the control group, confirming its good in vivo biocompatibility (Figs. S54a and S55).

The in vivo therapeutic mechanisms were also explored. Intratumoral NAD^+^/NADH and GSH analyses and DCFH-DA staining demonstrate that PtSNC can effectively disrupt the NAD^+^/NADH homeostasis, deplete GSH, and induce oxidative stress in tumors (Figs. 7k, S56, and S57), consistent with its tumor-selective NOX-/OXD-/POD-like multi-catalysis-driven metabolic and redox interventions. It hints that PtSNC could implement the bioenergetic disturbing within tumors. The histopathological evaluations involving hematoxylin–eosin (H&E) and Ki67 staining expose substantial tumor cell damage and suppressed proliferation in the PtSNC group, of which the effects far exceeded those of the DOX group (Fig. 7l). In addition, the terminal deoxynucleotidyl transferase-mediated dUTP nick end labeling (TUNEL) staining and GPX4 immunofluorescence analyses prove that PtSNC is capable of concurrently activating apoptosis and ferroptosis in vivo, aligning with the in vitro experiments (Fig. 7l). These results collectively manifest the potential of PtSNC as a potent and biosafe therapeutic agent for suppressing malignant tumors by catalytic bioenergetic intervention.

### Concurrent Tumor Recurrence Inhibition/Wound Healing Acceleration in a Diabetic Postoperative Model

Owing to the profound metabolic disparities, diabetes fosters a postoperative milieu where residual tumor cells and normal cells exhibit divergent bioenergetic dynamics, creating a pro-tumor recrudescing and anti-wound healing status. Based on the validated microenvironments-adaptive tumor-inhibiting and repair-promoting capabilities of PtSNC via bi-polar bioenergetic intervention, we then established a diabetic tumor resection model using C57BL/6 mice to assess its combined therapeutic functions. Following STZ-induced diabetes, B16F10 cells were implanted into these mice to build dorsal melanomas which were partially resected to generate residual melanomas alongside circular skin wounds (Fig. 7m). Postoperative treatments included untreated controls (HG group), Vc (HG + Vc group), DOX (HG + DOX group), and PtSNC (HG + PtSNC group), with the non-diabetic mice as healthy controls (NG group). PtSNC-treated diabetic wounds exhibited progressive closure without tumor recurrence, nearing complete healing by day 12, whereas other groups including the NG group retained unhealed, scabbed wounds with residual tumors (Fig. 7q, s). The HG + PtSNC group displayed the smallest unhealed wound area (Fig. 7s), underscoring the superior chronic wound repair-promoting capacity of PtSNC. Tumor growth curves reveal an accelerated tumor progression in the HG group compared to the NG group (Fig. 7n), confirming that hyperglycemia favors tumor recurrence. The anti-tumor efficacy varied across treatments: Vc showed the minimal effect, DOX exhibited the moderate tumor suppression, and PtSNC possessed the most significant tumor-inhibiting ability (98.3% suppression rate) (Fig. 7n-p). Remarkably, PtSNC treatment eradicated tumors in several diabetic mice without relapse (Fig. 7r), demonstrating its excellent tumor-suppressing effectiveness even under pro-tumorigenic hyperglycemic conditions. Furthermore, PtSNC treatment significantly prolonged the postoperative survival of these treated mice (Fig. 7t).

The histopathological analyses including H&E, Ki67, and TUNEL staining demonstrate the intact tumor tissue structures with clear cell nuclei in the NG, HG, and HG + Vc groups, whereas DOX induced a portion of tumor cell death and PtSNC caused extensive nuclear fragmentation, structural disintegration, and maximal apoptotic rate (Fig. S58). The most obvious suppression of Ki67 expression was observed in the PtSNC-treated tumors, suggesting its robust interception of tumor proliferation (Fig. S58). Moreover, PtSNC treatment did not perturb blood glucose levels, body weight, and histologic characteristics of major organs, affirming again the high biosafety (Figs. S59 and S60). The results establish that PtSNC as a biocompatible double-effect therapeutic agent can suppress tumor recurrence by eradicating residual malignant cells and synchronously accelerate diabetic wound healing—a promising dual-pronged strategy showing attractive potential for diabetic tumor postoperative management.

### Biosafety Profile, Translational Prospects, and Remaining Challenges of PtSNC

A comprehensive long-term safety assessment was performed to evaluate the in vivo biosafety of PtSNC. In diabetic mice receiving repeat intravenous doses of PtSNC (up to 500 μg mL^−1^) over 31 days, no significant exacerbation of diabetes-associated body weight loss was observed, and blood glucose levels remained stably high, confirming that PtSNC did not induce acute systemic toxicity (Fig. S61). Terminal hematological and serum biochemical analyses at day 31 revealed no statistically significant alterations in the blood-related parameters and key liver/kidney function indexs compared to diabetic controls (Figs. S62 and S63). Histopathological examination of major organs further showed no evidence of inflammation, necrosis, or other pathological lesions attributable to PtSNC administration (Fig. S64). The biodistribution and clearance profile was analyzed using the Cy5-labeled PtSNC. The results of in vivo fluorescence imaging showed rapid signal reduction in the abdominal region from 2 to 24 h, indicating effective systemic clearance (Fig. S65a). The ex vivo imaging results revealed a time-dependent transition: initial strong renal accumulation at 2 h, followed by increased hepatic and splenic signal at 12 h, and ultimately substantial clearance from all organs by 24 h (Fig. S65b). Importantly, the fluorescence signal was also detected in feces at 24 h, providing direct evidence of excretory clearance (Fig. S65c). Therefore, PtSNC demonstrates a highly favorable long-term biosafety profile, with no detectable chronic toxicity at a relatively high dose and an efficient clearance pathway via hepatic and renal excretion, minimizing the risk of long-term accumulation.

Collectively, the therapeutic efficacy and biosafety data underscore the translational promise of PtSNC. Its synthesis via a scalable porphyrinic MOF-based mixed-ligand strategy ensures the reproducible production of catalytically critical, atomically dispersed Pt-N_4_ sites. The absence of chronic toxicity even upon repeated systemic exposure in a sensitive diabetic model suggests a low risk of immunogenicity and supports its therapeutic safety window. Critically, the therapeutic paradigm demonstrated in this work is already aligned with a practical clinical delivery route. In our in vivo therapeutic studies, PtSNC was applied via direct spray onto the wound and resection area, followed by occlusive dressing, which represents a method directly adaptable to postoperative management. For future clinical translation, this approach can be further optimized by formulating PtSNC into sprayable, biocompatible hydrogels or in situ-forming depots, which would enhance retention at the surgical site, provide sustained release, and further minimize systemic exposure, thereby maximizing the therapeutic outcome and patient compliance.

Complementing these translational advances, a deeper mechanistic insight into PtSNC’s in vivo behavior elucidates how it achieves microenvironments-selective bi-polar efficacy and also highlights remaining challenges for further optimization. Mechanistically, the pathology self-adaptive bi-polar therapeutic outcome of PtSNC arises from two integrated layers of selectivity: (i) pH-dependent catalytic adjustment, directing OXD-/POD-like pro-oxidative catalysis to acidic tumors and CAT-/SOD-like antioxidative catalysis to neutral wounds; and (ii) cell-intrinsic metabolic decoding, wherein tumor cells and hyperglycemia-impaired normal cells translate the same catalytic NADH oxidation event into opposite outcomes, i.e., lethal energy collapse versus metabolic recovery. This dual-layer mechanism enables functional selectivity even when tumor remnants and regenerating tissues coexist in close spatial proximity. In practice, absolute catalytic switching is unattainable in vivo, and low-level off-target activity inevitably exists. Nonetheless, the predominant context-appropriate catalytic mode, together with cell-type-specific metabolic resilience, ensures robust bi-polar therapeutic efficacy. Future efforts, e.g., active-site engineering to sharpen catalytic contrast, incorporation of targeting ligands, and development of activatable pro-drug nanocatalysts, will further enhance precision and translatability, paving the way for next-generation catalytic nanomedicines tailored to complex pathological milieus.

## Conclusions

This proof-of-concept study introduces PtSNC, a single-atom nanocatalyst engineered with pathological contexts-selective multienzyme-mimicking activities, as a promising solution to the metabolic dilemma of promoted tumor recurrence and impaired wound healing in diabetic cancer patients. Synthesized via a porphyrinic MOF-based mixed-ligand strategy, PtSNC features high-density atomically dispersed Pt-N_4_ sites that enable microenvironments-dependent catalysis: in acidic and NADH-overexpressed tumor niches, its NOX-/OXD-/POD-mimicking activities can deplete NADH and generate highly reactive Pt = O species to disrupt glycolysis/OXPHOS-driven ATP synthesis and induce apoptosis/ferroptosis; in neutral and NAD^+^/NADH-dysregulated diabetic wounds, its NOX-/CAT-/SOD-like cascades can rectify cellular NAD^+^/NADH abnormity and bioenergetic disorder incurred by hyperglycemia, accelerates ATP supply, depletes excess ROS, and replenishes O_2_. In murine models of diabetic melanoma resection, PtSNC achieved a near-complete tumor recurrence inhibition (98.3% suppression rate), and much accelerated surgical wound healing, outperforming conventional treatments while maintaining high biosafety. Therefore, PtSNC as a highly biocompatible and efficacious nanoformulation can be used for synchronously suppressing surgical tumor recurrence and accelerating diabetic wound healing, which provides an encouraging therapeutic option for clinical diabetic oncologic postoperative treatment.

While numbers of studies have engineered nanozymes (including M–N-C single-atom nanomaterials) with diverse enzyme-mimicking activities—such as CAT-, SOD-, or POD-like functionality for redox modulations [26, 81–83], glucose oxidase-, lactate oxidase-, or hydrolase-like property for metabolic substrate regulations [84–87]—to manipulate pathological microenvironments for treating tumors, chronic wounds, bacterial infection, or other diseases, few cases, however, have being successful in solving the metabolic delimma stemming from the bifurcated pathological microenvironments through the self-selective multi-catalytic activities. More importantly, to our knowledge, no previous work has reported using a single material to achieve bidirectional interventions on such a bifurcated bioenergetic landscape. Herein, PtSNC distinguishes itself from the previous reports, not only by its flexible multienzyme-like catalytic activities but also by its unique capacity to specifically solve the bioenergetic metabolic predicament through adaptive calaytic activities to the almost contradictory microenvionments between tumor tissues and diabetic wounds without reliance on external stimuli, underscoring the potential of single-atom catalytic medicines in combating multifactorial diseases featuring opposite pathological characters. Such a pioneering bi-polar bioenergetic intervention strategy of concurrently counterattacking tumor aggression and promoting diabetic wound healing offers an unprecedented paradigm for addressing the diseases characterized by metabolic bifurcation (e.g., obesity-associated cancers, infectious inflammatory diseases, or other metabolic syndromes).

## Supplementary Information

Below is the link to the electronic supplementary material.

Supplementary file1 (DOCX 9669 KB)
