# Supplementary material for: Bi-Polar Bioenergetic Intervention via a Pathology Self-Adaptive Single-Atom Nanocatalyst for Diabetic Tumor Postoperative Management
Source: Nanomicro Lett. 2026 Apr 28;18:346. doi: 10.1007/s40820-026-02169-w (PMC13125485; doi:10.1007/s40820-026-02169-w)
Supplement: Supplementary file 1 — Supplementary file1 (DOCX 9669 KB) [file 40820_2026_2169_MOESM1_ESM.docx]

Supporting Information for

**Bi-Polar Bioenergetic Intervention via a** **Pathology Self-****Adaptive Single-Atom Nanocatalyst for Diabetic Tumor Postoperative** **Management**

Jiajie Chen^1,3,#^, Jimin Huang ^1,6,#^, Zhibo Yang^1^, Kai Tang^1,2^, Chengtie Wu^1,2^, Huamao Ye^5^*, Jianlin Shi^1,4^*, and Yufang Zhu^1,2^*

^1^ State Key Laboratory of High Performance Ceramics, Shanghai Institute of Ceramics, Chinese Academy of Sciences, Shanghai 200050, P. R. China

^2^ Center of Materials Science and Optoelectronics Engineering, University of Chinese Academy of Sciences, Beijing 100049, P. R. China

^3^ Chair of Inorganic Chemistry I, Technische Universität Dresden, Bergstrasse 66, Dresden, 01069, Germany

^4^ Shanghai Frontiers Science Center of Nanocatalytic Medicine, Shanghai Tenth People’s Hospital, School of Medicine, Tongji University, Shanghai 200331, P. R. China

^5^ Department of Urology, Changhai Hospital, Second Military Medical University, Shanghai 200433, P. R. China

^6^ School of Pharmacy, Xuzhou Medical University, Xuzhou 221004, P. R. China

^#^ Jiajie Chen and Jimin Huang contributed equally to this work.

* Corresponding authors. E-mail: [yhm@smmu.edu.cn](mailto:yhm@smmu.edu.cn) (Huamao Ye); [jlshi@mail.sic.ac.cn](mailto:jlshi@mail.sic.ac.cn) (Jianlin Shi); [zjf2412@163.com](mailto:zjf2412@163.com) (Yufang Zhu)

**S1 S****upplementary Methods**

**S1.1 Characterizations**

Transmission electron microscopy (TEM) and high-resolution TEM (HRTEM) images with selected-area electron diffraction (SAED) patterns were acquired using JEM-2100F electron microscope (JEOL, Japan). Spherical aberration-corrected high-angle annular dark-field scanning TEM (HAADF-STEM), energy-dispersive X-ray spectroscopy (EDS) profiles, and elemental mapping were performed on Hitachi-HF5000 (Japan). Scanning electron microscopy (SEM) images and associated EDS profiles were obtained using SU9000 electron microscope (Hitachi, Japan). X-ray diffraction (XRD) patterns were recorded on Ultima IV X-ray diffractometer (Rigaku, Japan). Raman spectra were obtained from in Via spectrometer (Renishaw, UK). X-ray photoelectron spectroscopy (XPS) measurements were conducted on K-Alpha instrument (Thermo Fisher Scientific, USA), and corresponding analyses were performed employing XPSPEAK software (version 4.0). Inductively coupled plasma mass spectrometry (ICP-MS) test was carried out using Agilent 7850 system (USA). Fourier-transform infrared (FTIR) spectra were collected on Nicolet iS10 spectrometer (Thermo Fisher Scientific, USA). In situ diffuse reflectance infrared Fourier-transform spectroscopy (DRIFTS) measurements were carried out on VERTEX 80v spectrometer (Bruker, Germany), where the sample was swept by H_2_O and O_2_ gas at 310 K in the dark for 60 min and the data were recorded in real time. Hydrodynamic diameter and zeta potential measurements were performed using Zetasizer Nano ZS90 system (Malvern Instruments, UK). UV-vis absorption spectra and values were acquired with either an ultraviolet spectrophotometer (UV-2550, Shimadzu Corporation, Japan) using quartz cuvettes or a microplate reader (Epoch, BIO-TEK, USA) using multi-orifice plates. Electron spin resonance (ESR) spectroscopy was performed on EMXplus-6/1 spectrometer (Bruker, Germany). Biological TEM (Bio-TEM) imaging of cellular ultrastructure was conducted using HT7800 electron microscope (Hitachi, Japan). Confocal laser scanning microscopy (CLSM) images was obtained from TCS SP8 confocal fluorescence microscope (Leica, Germany). Cell and tissue observations were made with DMi8 inverted microscope (Leica, Germany).

**S1.2 X-ray absorption fine structure (XAFS) measurements**

Pt L3-edge XAFS measurements, including X-ray absorption near-edge structure (XANES) and extended X-ray absorption fine structure (EXAFS), were performed at the beamline BL11B (Shanghai Synchrotron Radiation Facility, China). Powder samples were pressed into thin sheets with 1 cm in diameter and sealed using Kapton tape film, and then recorded in transmission mode using a Bruker 5040 4-channel Silicon Drift Detector (SDD). Negligible changes in the line-shape and peak position of Pt L3-edge XANES spectra were found between two scans taken for a specific sample. Raw data were processed using Athena software (version 0.9.26) for background, pre-edge line and post-edge line calibrations. The Fourier transformed fitting was conducted in Artemis software (version 0.9.26) [S1, S2]. The parameters were listed as follow: *R* range, 1.0-2.4 Å; *k* range, 3.0-10.8 Å^-1^; *k^3^*-weighting. Reference models included Pt foil (Pt-Pt), PtO_2_ (Pt-O), and TCPP(Pt) (Pt-N). The four parameters involving coordination number (CN), bond length (*R*), Debye-Waller factor (*σ*^2^), and E_0_ shift (ΔE_0_) were fitted and partially fixed. Furthermore, for wavelet transformation analysis, the *χ*(*k*) exported from Athena was imported into the Hama Fortran code, and the parameters were listed as follow: *R* range, 1.0-3.0 Å; *k* range, 0-13.9 Å^-1^; *k* weight, 3. The Morlet function with *κ* = 5, *σ* = 1 was used as the mother wavelet to offer the overall distribution. The authors would like to thank Shiyanjia Lab (www.shiyanjia.com) for the support of XAFS measurements.

**S1.3 Total antioxidant performance of PtSNC**

The total antioxidant capacity of PtSNC was evaluated via ABTS radical scavenging assay. ABTS working solution was prepared by mixing 38.4 mg mL^-1^ ABTS (reagent I) with 13.4 mg mL^-1^ potassium persulfate (reagent II) at 1:1 v/v, followed by 12 h dark incubation. PtSNC (100 μg mL^-1^) was incubated with 4 μL ABTS solution in the sodium acetate buffers (1 mL, pH 4.0-9.0) for 30 min in the dark. After centrifugation, the 730 nm absorbance of obtained supernatant (100 μL) was measured with the untreated ABTS solution as a control. ABTS radical scavenging capacity (i.e., total antioxidant activity) was quantified by the absorbance-reducing percentage.

**S1.4 ESR test**

The •OH generation was detected by ESR spectroscopy. PtSNC (25 μg mL^-1^) was dispersed in the sodium acetate buffer (pH 5.2) with or without H_2_O_2_ (10 mM). The mixture was treated with an equal volume of 5,5-dimethyl-1-pyrroline-*N*-oxide (DMPO) (100 mM in H_2_O) as a spin-trapping agent before the ESR test.

**S1.5 Catalytic GSH oxidation**

The catalytic GSH oxidation by PtSNC was tested using Ellman’s assay. In details, PtSNC (0-200 μg mL^-1^) was incubated with GSH (0.2 mM) in the Tris-HCl buffers (10 mM) at 37 °C for 30 min. Besides, PtSNC (50 μg mL^-1^) was reacted with GSH (0.2 mM) in the Tris-HCl buffers at 37 °C for different durations (0, 5, 15, 30, 60, and 90 min). Post-reaction mixtures were treated with 10 μL DTNB test solution (10 mM in DMSO) for 3 min at room temperature. The UV-vis spectra (300-550 nm) were acquired, and GSH-eliminating rate was calculated from the 412 nm absorbance reduction.

**S1.6 Computational details**

All DFT calculations were carried out using the Vienna *Ab-initio* Simulation Package (VASP), employing the projector augmented-wave method [S3]. The generalized gradient approximation proposed by Perdew, Burke, and Ernzerhof was chose for the exchange-correlation potential [S4]. The long-range van der Waals interactions were described by the DFT-D3 method [S5]. A plane-wave cutoff energy of 400 eV was applied, and the electronic energy convergence criterion was set to 10^-6^ eV during iterative solution of the Kohn-Sham equation. To avoid artificial interaction between periodic images, a vacuum layer of 15 Å was introduced normal to the sheet. The Brillouin zone integration was carried out using k-mesh with the K-spacing value of 0.04. Structural relaxations were considered complete when residual atomic forces fell below 0.03 eV Å^-1^.

**S1.7 Tumor cell-killing effect of PtSNC**

B16F10 cells were seeded in 96-well plates at 1 × 10^4^ cells per well and cultured for 24 h. The medium was replaced with 100 μL fresh HG medium (pH 7.4) containing varying concentrations of PtSNC, followed by 24 h incubation before viability measurement. In addition, the mildly acidic HG medium (pH 6.0) supplemented with H_2_O_2_ (100 μM) as the simulated TME were prepared to treat B16F10 cells for comparisons. To further elucidate the cell death pathways, rescue experiments were performed using specific inhibitors: B16F10 cells were pre-treated for 2 h with ferrostatin‑1 (1 μM), Z‑VAD‑FMK (20 μM), necrostatin‑1 (20 μM), or *N*‑acetyl‑L‑cysteine (NAC, 2 mM), followed by co‑incubation with PtSNC (100 μg mL^-1^) for 24 h prior to viability measurement.

**S1.8 Intracellular ROS staining**

HUVECs were seeded in 24-well plates and cultured for 72 h in the HG medium (the NG medium as a control). After different treatments for additional 12 h, these cells in different groups were incubated with DCFH-DA (10 μM) for 20 min at 37 °C. After three washes with PBS, intracellular ROS levels were measured via fluorescence microscope. Differently, B16F10 cells were seeded in 24-well plates and cultured for 24 h, and subsequently the medium was replaced with the fresh HG medium (pH 7.4) or the TME-simulated HG medium (pH 6.0, 100 μM H_2_O_2_) containing PtSNC (50 μg mL^-1^) followed by 5 h incubation before DCFH-DA treatment and fluorescence observation.

**S1.9 Mitochondrial tracking in HUVECs**

Following the same cell treatments applied for ROS staining assay, HUVECs were co-stained with Mito-Tracker Deep Red 633 (200 nM, Beyotime)—a novel deep red fluorescent probe that can specifically label mitochondria in live cells and indicate mitochondrial membrane potential—and Hoechst 33342 (5 μg mL^-1^, Beyotime) for mitochondrial and nuclear labeling before fluorescence observation.

**S1.10 JC-1 assay**

Based on JC-1 probe, red fluorescence can be detected in normal mitochondrial membranes with high potential (JC-1 aggregate) while green fluorescence can be found in damaged mitochondrial membranes with low potential (JC-1 monomer). In details, after treated with PtSNC (50 μg mL^-1^) in the HG medium (pH 7.4) or the TME-simulated HG medium (pH 6.0, 100 μM H_2_O_2_) for 5 h, B16F10 cells were incubated with JC-1 working solution according to the manufacturer’s protocol before fluorescence observation and imaging.

**S1.11 Visualization of mitochondrial morphology**

For fluorescence imaging, as-treated HUVECs and B16F10 cells were incubated with MitoTracker Red CMXRos (50 nM, Invitrogen) for 20 min at 37 °C, and then stained with Hoechst 33342 for 5 min at 37 °C before observation. Additionally, these cells were also collected by centrifugation and fixed with glutaraldehyde fixative (2.5%) for embedding, sectioning, staining, and bio-TEM imaging.

**S1.12 Detection of intracellular oxygenation in HUVECs**

The oxygen sensor [Ru(dpp)_3_]Cl_2_ was employed to detect intracellular O_2_ generation, leveraging its O_2_-dependent fluorescence quenching performance. HUVECs were seeded in 24-well plates and cultured in the HG medium for 48 h, and then these cells were exposed to the H_2_O_2_ (100 μM)-containing HG medium for 12 h under four conditions: 1) normoxia (21% O_2_); 2) hypoxia (2% O_2_); 3) hypoxia + 0.1 mM Vc; 4) hypoxia + 8 μg mL^-1^ PtSNC. Subsequently, the treated cells were incubated with [Ru(dpp)_3_]Cl_2_ (8 μg mL^-1^) for 4 h at 37 °C. After three PBS washes, these cells were fixed with 4 wt% paraformaldehyde solution (10 min) and further stained with 4′,6-diamidino-2-phenylindole (DAPI) (5 min) before fluorescence imaging on microscope.

**S1.13 Test of cellular GSH level in tumor cells**

B16F10 cells were seeded in 12-well plates at 5 × 10^4^ cells per well and treated with varying concentrations of PtSNC for 5 h. After treatments, cells were washed with PBS and lysed in 0.4% Triton-X-100 buffer (4 °C, 10 min). Lysates were centrifuged (4 °C, 12000 rpm, 5 min), and supernatants were reacted with 10 μL DTNB test solution (10 mM in DMSO) for 3 min. The relative GSH levels were quantified by measuring characteristic absorbance at 412 nm.

**S1.14 Western blot assay**

For GPX4 protein analysis, B16F10 cells were incubated with or without PtSNC (100 μg mL^-1^) for 24 h. In the function-blocking experiments using FK866, HUVECs were preconditioned in the HG medium for 48 h, followed by 3 days of different treatments before ATP level and specific protein analyses. In the function‑rescuing experiments using NMN, B16F10 cells in the TME-simulated HG medium accepted different treatments for 24 h before ATP level and specific protein analyses. After these different treatment processes, the total proteins of cells were extracted by RIPA lysis buffer (Beyotime) and further quantified using a bicinchoninic acid (BCA) assay kit (Thermo Fisher Scientific Inc.). Then, typical Western blot assay was performed by electrophoresis run on denaturing polyacrylamide gels to visualize the comparable results with a Tanon 4800 imaging system (China).

**S1.15 Cell proliferation, migration, and invasion assessments**

For cell proliferation assay, HUVECs seeded in 96-well plates were preconditioned in the HG medium for 48 h, followed by 5-day treatments: 1) NG; 2) HG; 3) HG + 0.1 mM Vc; 4) HG + 8 μg mL^-1^ PtSNC. Cell viability was quantified at days 1, 3, and 5 by CCK-8 assay. Cell migration ability was evaluated by an in vitro wound healing assay. HUVECs in 6-well plates were preconditioned in the HG medium for 48 h. Then, uniform scratches were created using a sterile 200 μL pipette tip, debris was removed with PBS, and scratches were imaged using inverted microscope. After different treatments as above for 24 h, these cells were fixed with 4 wt% paraformaldehyde solution (10 min), stained with 0.1 wt% crystal violet solution (3 min), and finally imaged. The migration ratio was calculated as follows: migration radio = (A_0_-A_1_)/A_0_ × 100%, where A_0_ = initial scratch area and A_1_ = residual scratch area. In addition, Transwell invasion assay was further performed. After 48 h incubation in the HG medium, HUVECs were digested and seeded onto the upper chamber of Transwell inserts with different incubations as above for 24 h. Afterwards, the non-invaded cells in the upper chamber were wiped off while the invaded cells on the lower surface were fixed with 4 wt% paraformaldehyde solution and stained with 0.1 wt% crystal violet solution. The stained cells were imaged and counted in the randomly selected areas.

**S1.16 Analysis of LPO and Caspase-3 activations in tumor cells**

Following the same cell treatments applied for ROS staining assay, B16F10 cells were treated with BODIPY™ 581/591 C11 and GreenNuc™ Caspase-3 detection agents, respectively, following the manufacturer’s protocols for fluorescent analysis of cellular LPO and Caspase-3 activations.

**S1.17 Live/dead cell staining assay**

After treated with PtSNC (50 μg mL^-1^) in the HG medium (pH 7.4) or the TME-simulated HG medium (pH 6.0, 100 μM H_2_O_2_) for 24 h, live and dead B16F10 cells were stained by Calcein-AM and propidium iodide (PI), respectively, before fluorescence observation and imaging.

**S1.18 In vivo anti-tumor evaluation**

B16F10 cells suspended in PBS were injected intradermally into the right back of hind leg area of BALB/c nude mice for in vivo orthotopic melanoma model building. Four days post-inoculation, these tumor-bearing mice were randomized into three groups (*n* = 6) for intratumoral injections as interventional treatments: 1) PBS (as a control group); 2) DOX (2 mg kg^-1^, a clinically relevant dose [S6]); 3) PtSNC (10 mg kg^-1^). The treatments were administered on days 0, 2, and 4. Tumor volumes and body weights were continuously measured until day 14, when mice were euthanized and their tumors were harvested, weighed, and finally sectioned for H&E/Ki67/TUNEL staining and GPX4 immunofluorescence analyses. Besides, major organs were harvested and sectioned for H&E staining analysis. In the parallel experiments, the treated mice were monitored for 30 days, with survival defined as tumor volume ≤ 1000 mm^3^. In addition, at 48 h post-treatment, tumors from each group were homogenized, and then intratumoral GSH levels and NAD^+^/NADH ratios were quantified using methods consistent with the in vitro assays; at 24 h post-treatment, the tumor regions in the different groups were in situ injected with 100 μL of DCFH-DA/PBS solution (10 μM) for 30 min before frozen slicing and fluorescent observation.

**S2 Supplementary Figures**


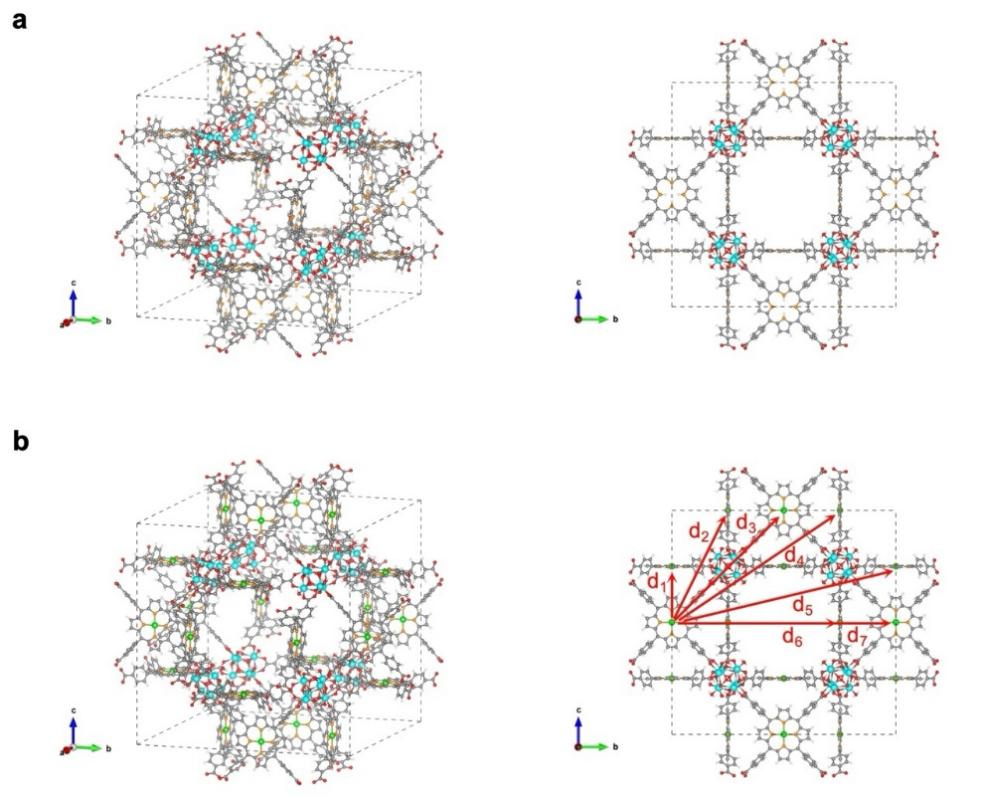


**Fig. S1** Schematic illustrations of the structures of a) PCN-224 with TCPP as ligand and b) PCN-224-Pt with TCPP(Pt) as ligand (d_1-7_: the possible distances between two Pt atoms in the adjacent TCPP(Pt) ligands in a PCN-224-Pt_x_ nanoparticle)


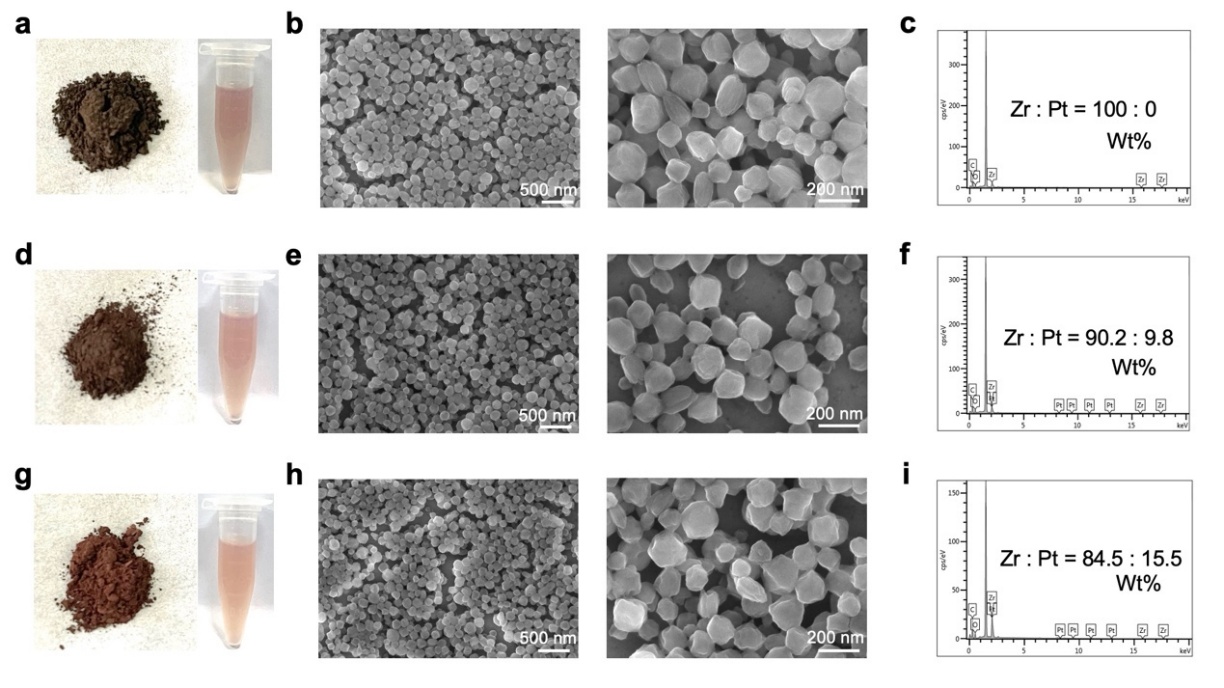


**Fig. S2** Powder photographs and ethanol dispersion liquids of a) PCN-224, d) PCN-224-Pt_20_, and g) PCN-224-Pt_40_. SEM images and corresponding EDS profiles of b, c) PCN-224, e, f) PCN-224-Pt_20_, and h, i) PCN-224-Pt_40_. Notably, the product color progressively shifted from yellowish-brown to reddish-brown with increasing TCPP(Pt) incorporation during synthesis, while these SEM images reveal no significant alterations in nanoparticle morphology.


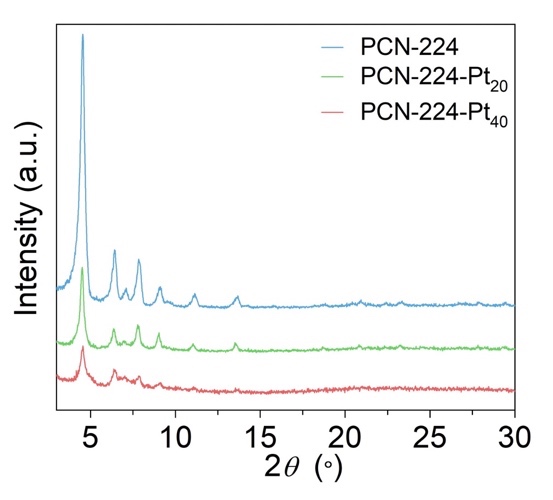


**Fig. S3** XRD patterns of PCN-224, PCN-224-Pt_20_, and PCN-224-Pt_40_


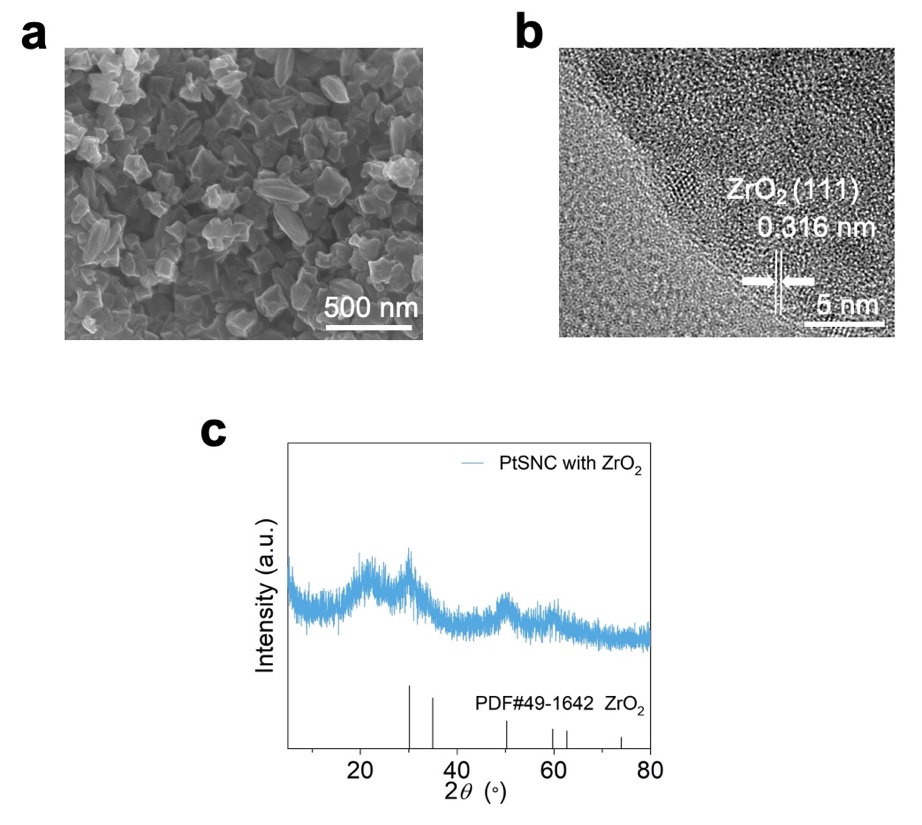


**Fig. S4** a) SEM image, b) HRTEM image, and c) XRD pattern of PCN-224-Pt_20_ after pyrolysis (i.e., ZrO_2_-containing PtSNC)


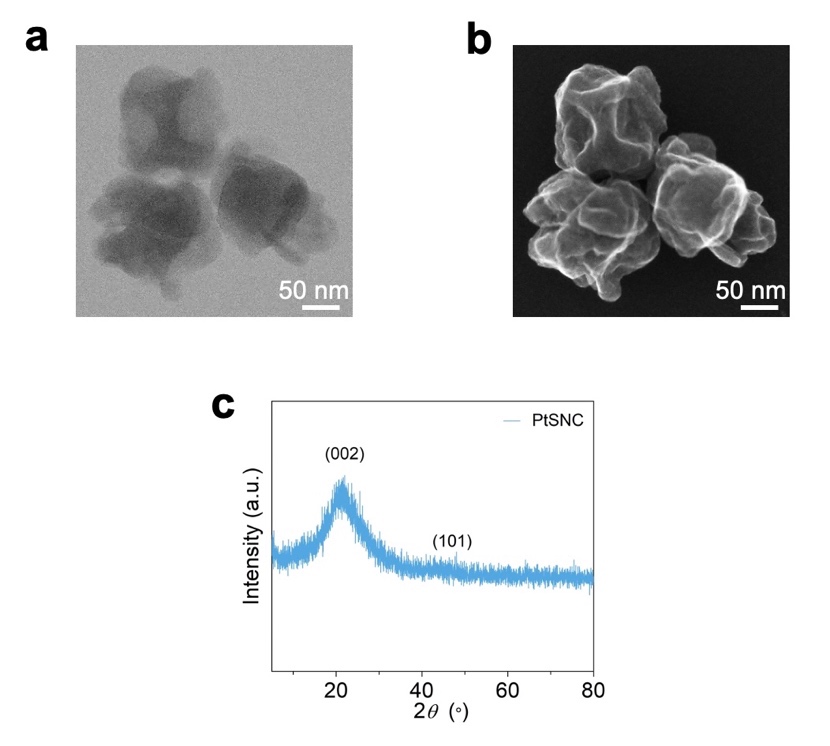


**Fig. S5** Spherical aberration-corrected TEM images of PtSNC in a) bright field and b) secondary electron modes. c) XRD pattern of PtSNC


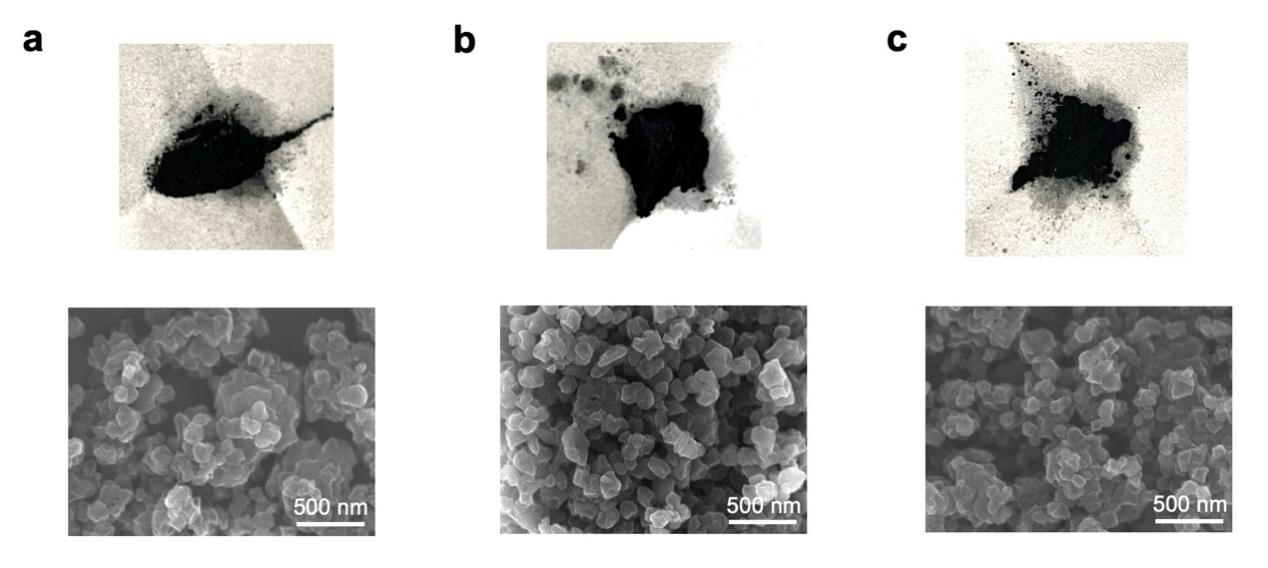


**Fig. S6** Powder photographs and SEM images of a) PCN-224, b) PCN-224-Pt_20_, and c) PCN-224-Pt_40_ after post-pyrolysis and acid etching procedures


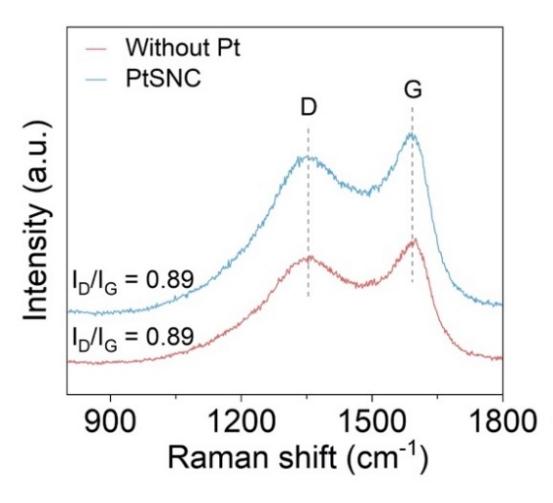


**Fig. S7** Raman spectra of PCN-224-derived Pt-free N-doped carbon nanoparticles and PtSNC


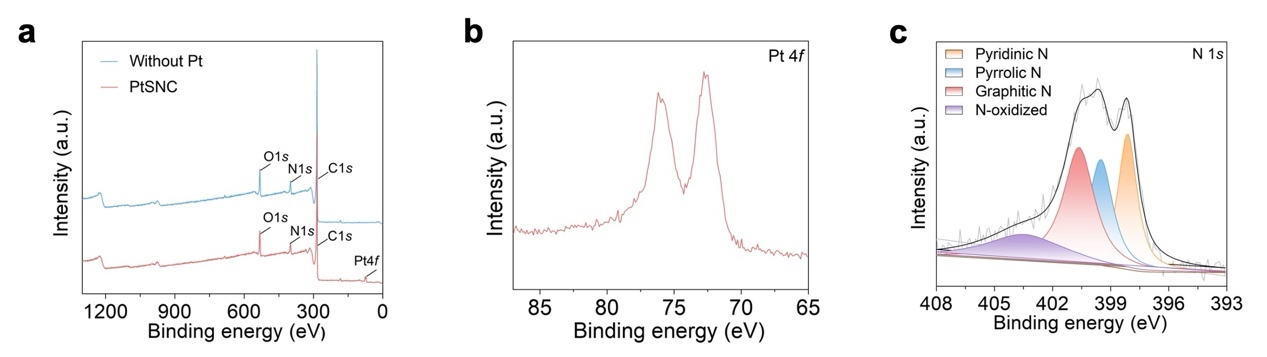


**Fig. S8** a) Full survey XPS spectra of different nanoparticles. b) Pt 4*f* XPS spectrum of PtSNC. c) N 1*s* XPS spectrum of PCN-224-derived Pt-free N-doped carbon nanoparticles


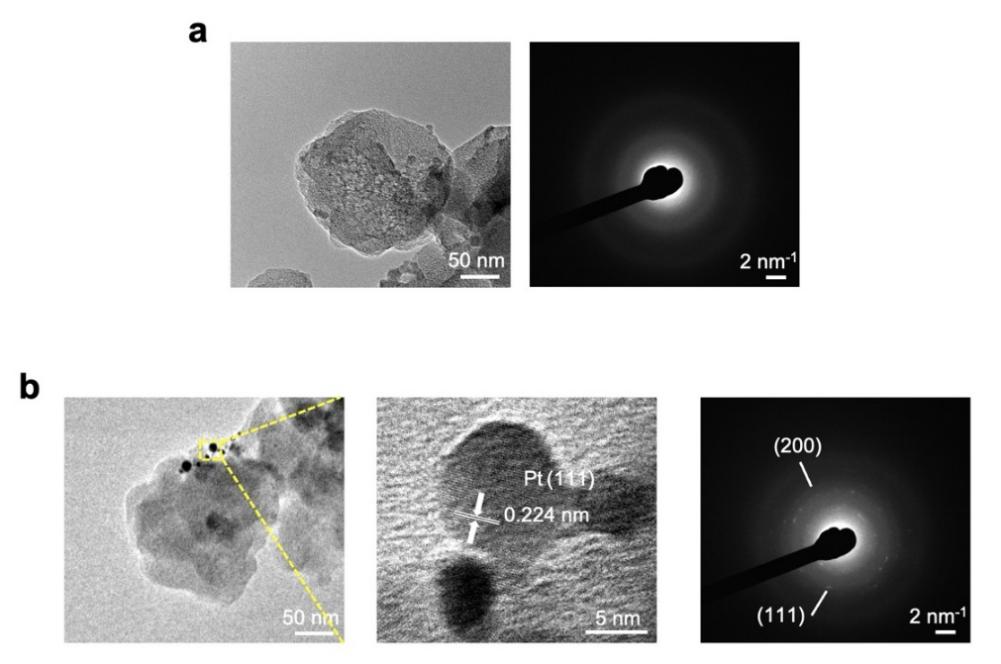


**Fig. S9** a) TEM image and corresponding SAED pattern of PCN-224-derived Pt-free N-doped carbon nanoparticles. b) TEM image, HRTEM image, and corresponding SAED pattern of PCN-224-Pt_40_-derived N-doped carbon nanoparticles


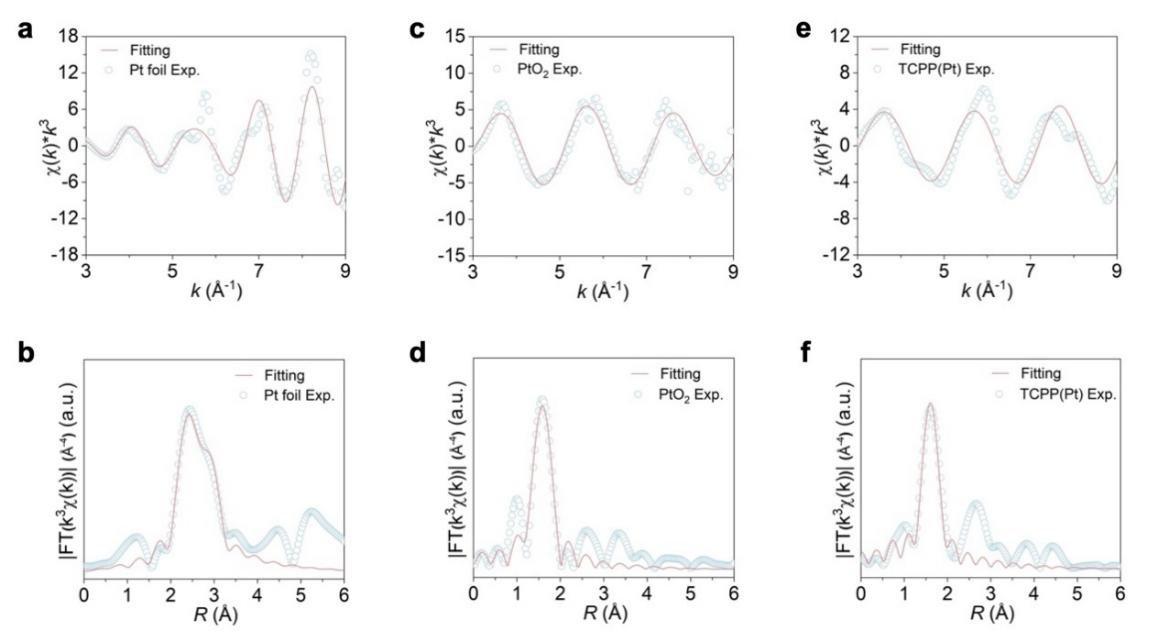


**Fig. S10** The a,c,e) *k*-space and b,d,f) *R*-space fitting results of Pt foil, PtO_2_, and TCPP(Pt)


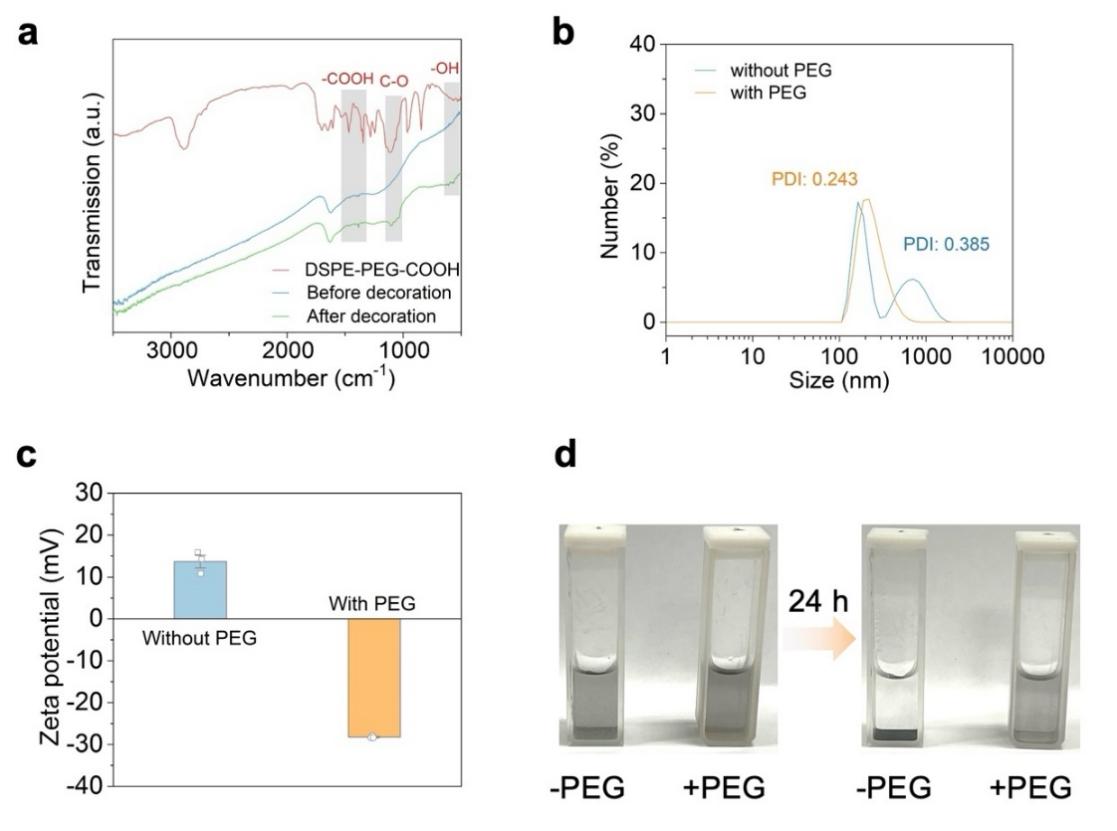


**Fig. S11** a) FTIR spectra, b) hydrodynamic size distributions, and c) zeta potentials of the PtSNC before and after PEGylation. PDI: polydispersity index. A smaller PDI means better uniformity and narrower dispersity width. d) Photographs of the PtSNC with or without PEG decoration dispersed in water before and after 24 h. The PEGylated PtSNC still remained stable after 24 h of incubation, while an obvious agglomeration phenomenon was observed for bare PtSNC, confirming the enhanced colloidal stability after PEGylation. Data are expressed as mean ± s.e.m. (*n* = 3 independent experiments)


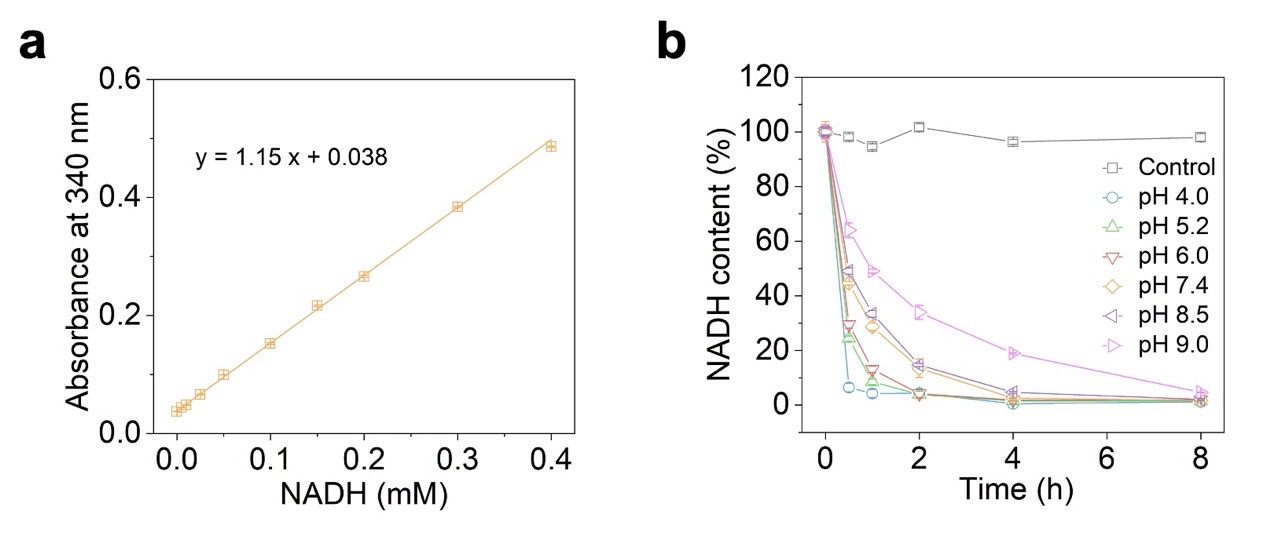


**Fig. S12** a) The linearly fitted standard curve of NADH characteristic absorption intensity at 340 nm *vs* concentration. b) PtSNC-catalytic NADH depletion at different pH conditions. Data are expressed as mean ± s.e.m. (*n* = 3 independent experiments in (a); *n* = 4 independent experiments in (b))


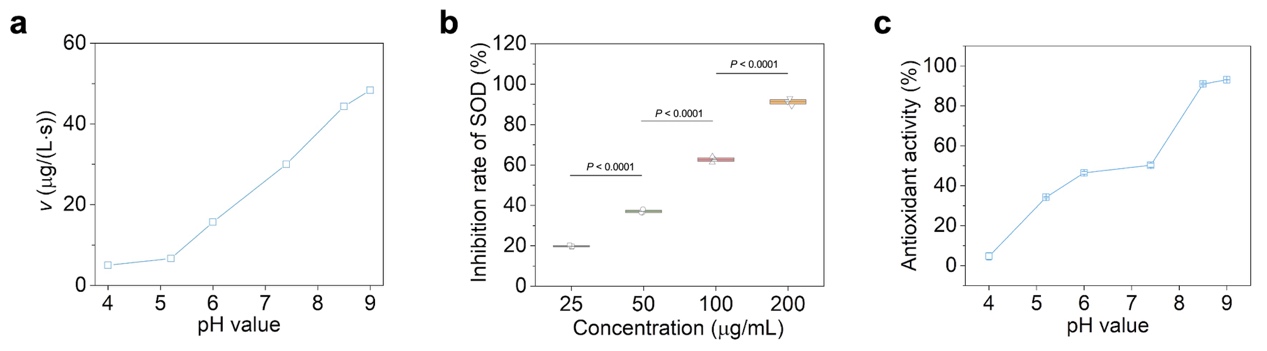


**Fig. S13** a) The initial O_2_-generating rates of CAT-like catalysis of PtSNC at different pH conditions. b) The calculated inhibition rates of SOD using a total SOD assay kit with WST-8 (Beyotime). A higher inhibition rate means better SOD-like activity. c) The calculated total antioxidant activities of PtSNC at different pH conditions in the ABTS radical scavenging assay. Data are expressed as mean ± s.e.m. (*n* = 3 independent experiments in (b); *n* = 4 independent experiments in (c)). Statistical significance was determined by one-way ANOVA with Tukey’s *post-hoc* test


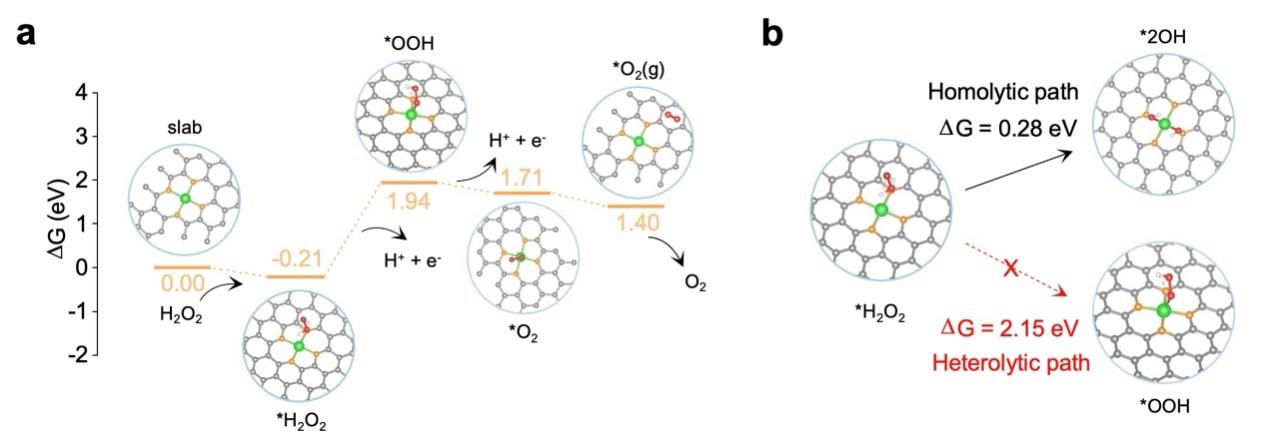


**Fig. S14** a) Proposed heterolytic path for CAT-like catalysis and the DFT-determined free energy diagram. b) Comparison of the rate-determining step free energy variations between proposed homolytic and heterolytic paths


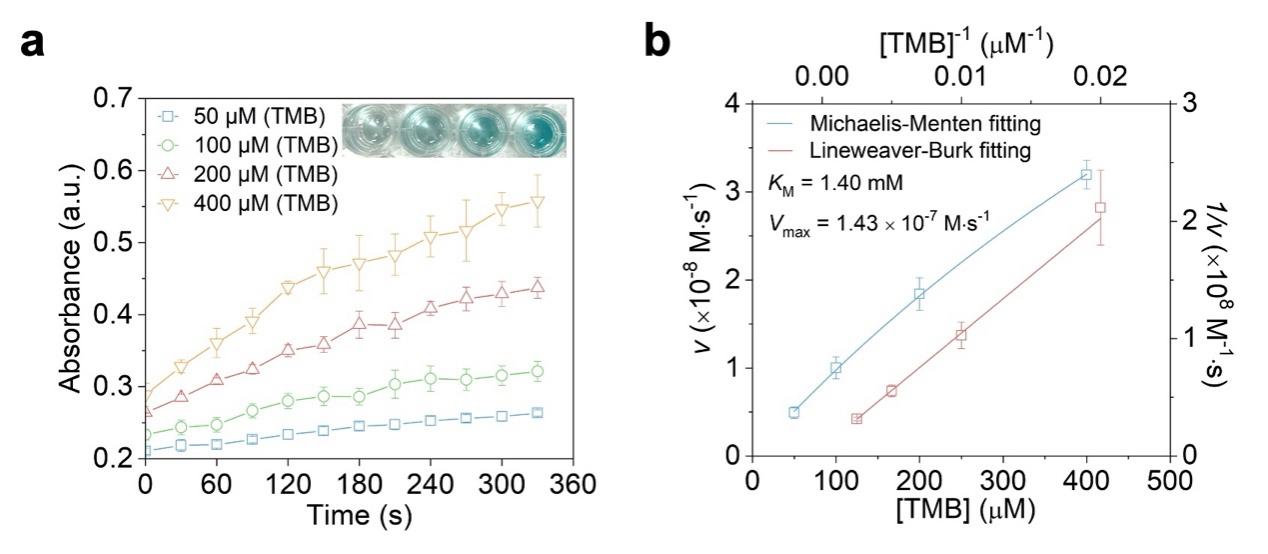


**Fig. S15** a) OXD-like catalysis of PtSNC with different concentrations of TMB as the reaction substrate. Inset: display of the test solutions after reactions. b) Michaelis-Menten kinetic analysis and Lineweaver-Burk plot of OXD-like activity of PtSNC. Data are expressed as mean ± s.e.m. (*n* = 3 independent experiments)


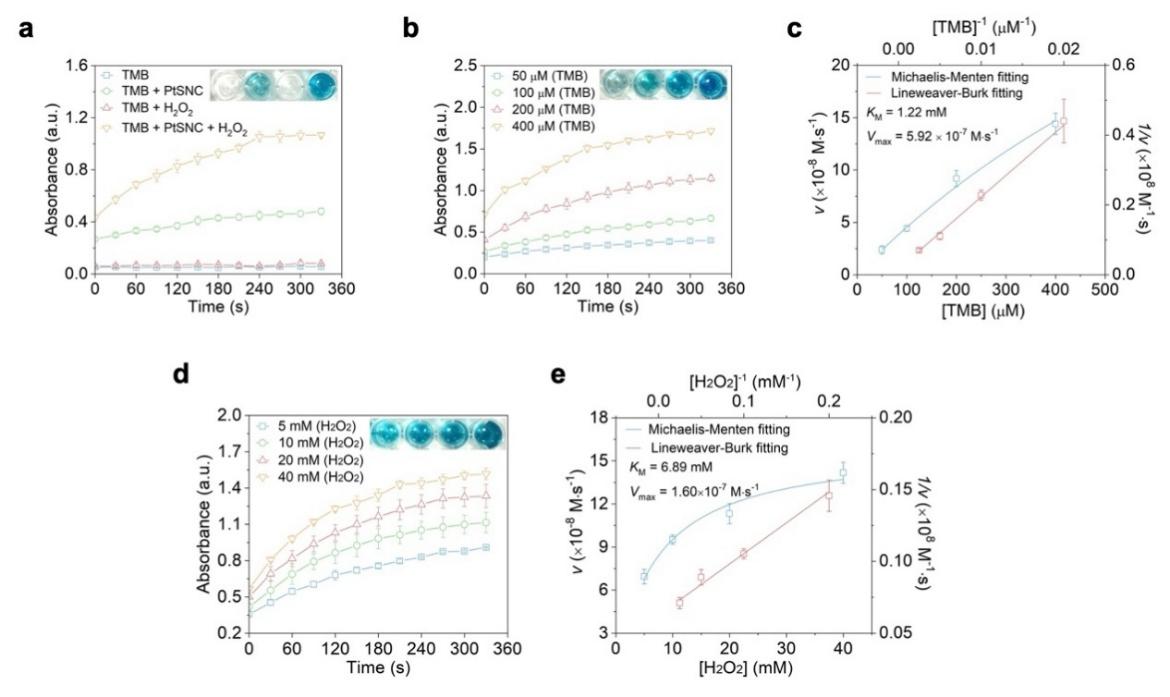


**Fig. S16** a) Time-dependent TMB oxidation process by PtSNC without or with adding H_2_O_2_. b) POD-like catalysis of PtSNC with different concentrations of TMB as the reaction substrate, and c) corresponding Michaelis-Menten kinetic analysis and Lineweaver-Burk plot. d) POD-like catalysis of PtSNC with different concentrations of H_2_O_2_ as the reaction substrate, and e) corresponding Michaelis-Menten kinetic analysis and Lineweaver-Burk plot. Inset: display of the test solutions after reactions. Data are expressed as mean ± s.e.m. (*n* = 3 independent experiments)


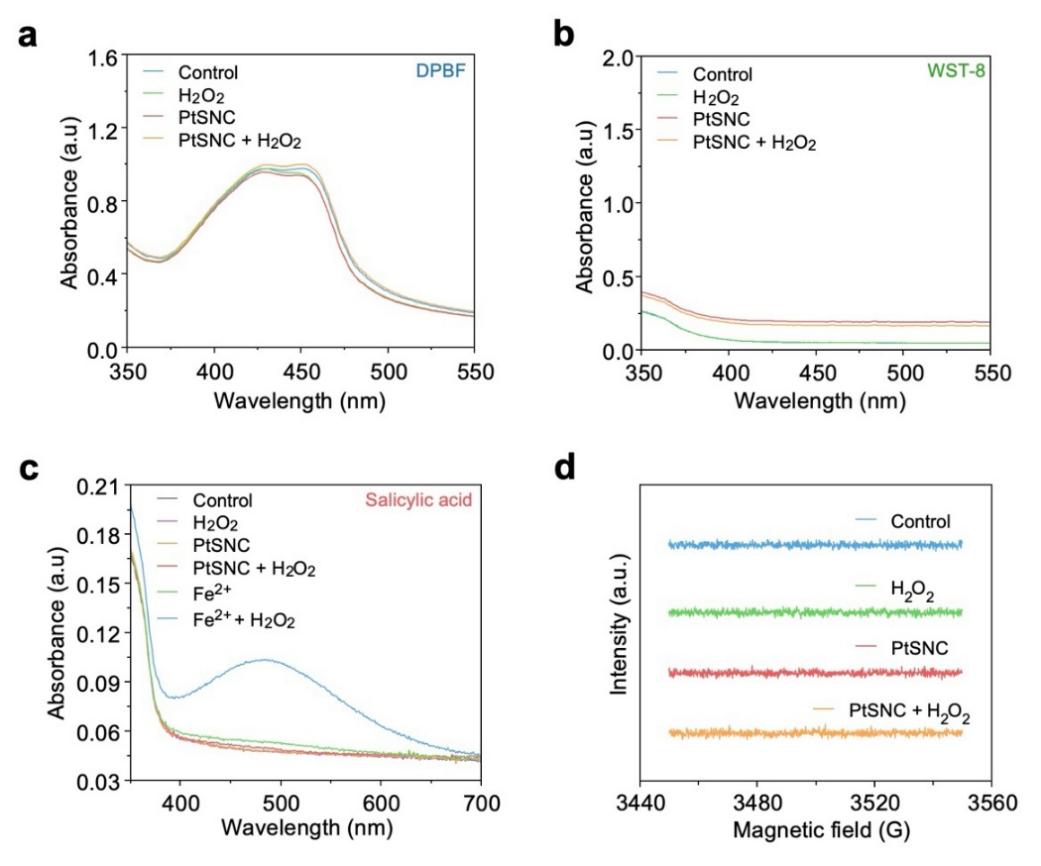


**Fig. S17** a) Verification of PtSNC-catalytic ^1^O_2_ generation using DPBF as a specific probe. b) Verification of PtSNC-catalytic O_2_•^-^ generation using WST-8 from a total SOD assay kit (Beyotime) as a specific probe. c) Verification of PtSNC-catalytic •OH generation using salicylic acid as a specific probe. d) ESR spectra evaluating PtSNC-catalytic •OH generation using DMPO as a spin-trapping agent. All the detection results demonstrate no free ROS including ^1^O_2_, O_2_•^-^, and •OH, generated in the PtSNC-mediated catalytic processes.


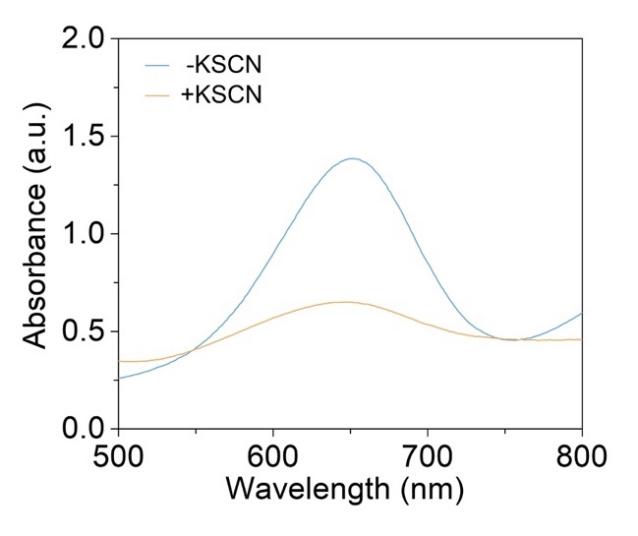


**Fig. S18** UV-Vis absorption spectra of PtSNC-treated H_2_O_2_/TMB solutions with and without the addition of KSCN. The POD-like activity of PtSNC was significantly impaired after KSCN treatment, due to the strong coordination of SCN^-^ with the Pt of PtSNC. It indicates that atomically dispersed Pt-N_4_ sites in the PtSNC are actual active catalytic centers in catalysis [S7]

**
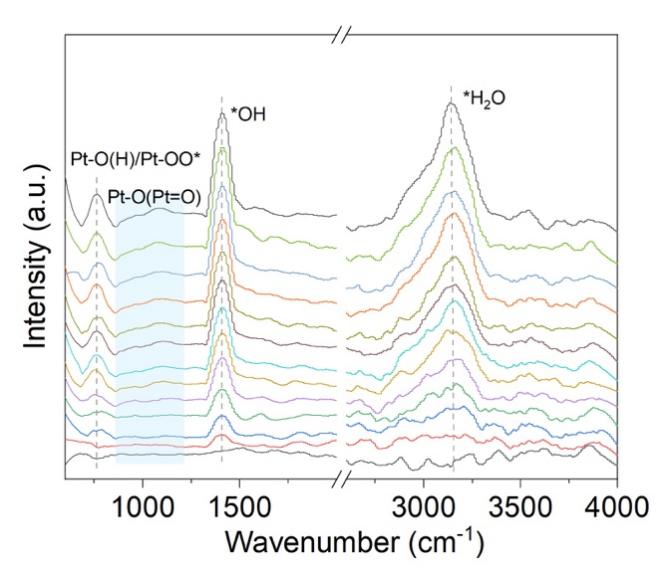
**

**Fig. S19** In situ DRIFTS spectra of the PtSNC-H_2_O interface collected at different time intervals at 310 K under O_2_ for 60 min. The bands corresponding to adsorbed H_2_O/hydroxyl-related species (3140 and 1410 cm^-1^) gradually appeared, accompanied by features associated with oxygen activation precursors Pt-O(H)/Pt-OO* (762 cm^-1^). Concurrently, a broad band in the 860-1210 cm^-1^ range emerged, indicating the formation of strongly bound Pt-O species associated with the proposed Pt=O intermediates


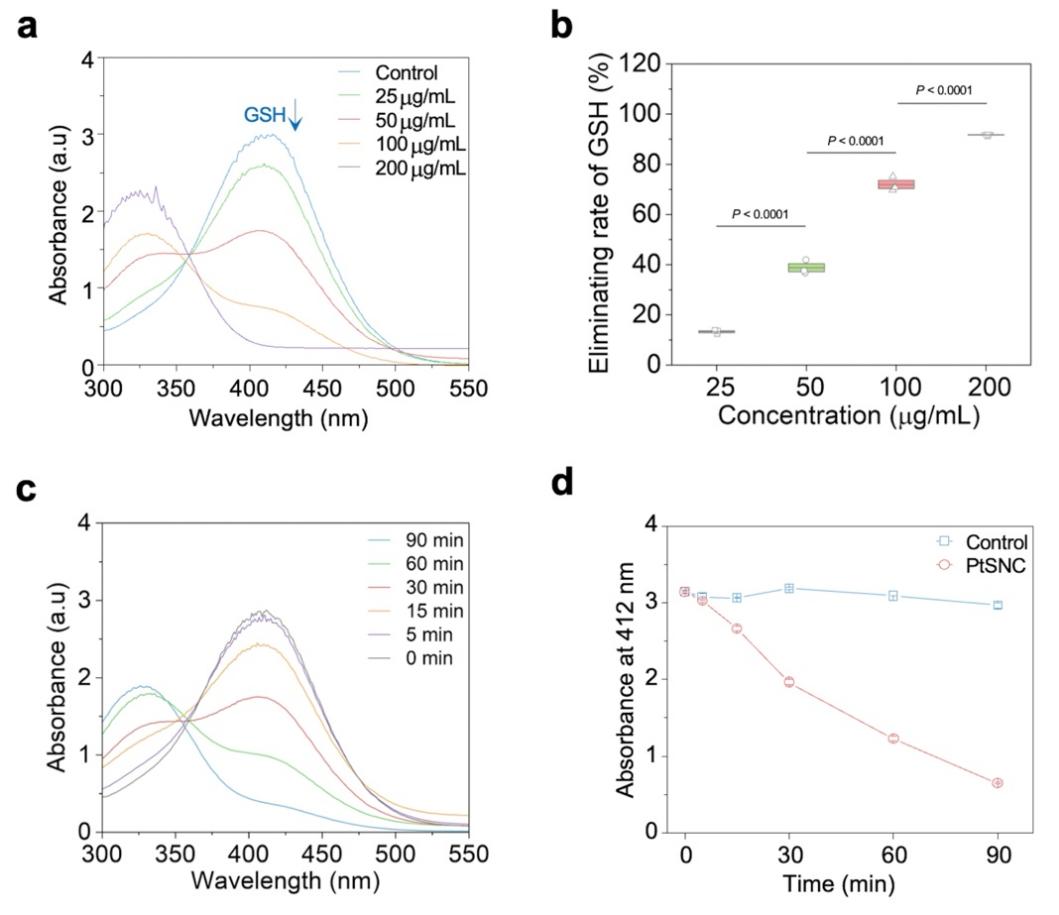


**Fig. S20** a) UV-Vis absorption spectra of the PtSNC-reacted GSH-containing test solutions with DTNB as a colorimetric GSH detection agent. The decrease in 412 nm characteristic absorption indicates a reduction in the concentration of GSH. b) The calculated GSH-eliminating rates after reaction with different concentrations of PtSNC. c) Time-dependent GSH oxidation mediated by PtSNC. d) Time-dependent changes of the characteristic absorption at 412 nm of the test solutions reacted with and without PtSNC. Data are expressed as mean ± s.e.m. (*n* = 3 independent experiments). Statistical significance was determined by one-way ANOVA with Tukey’s *post-hoc* test


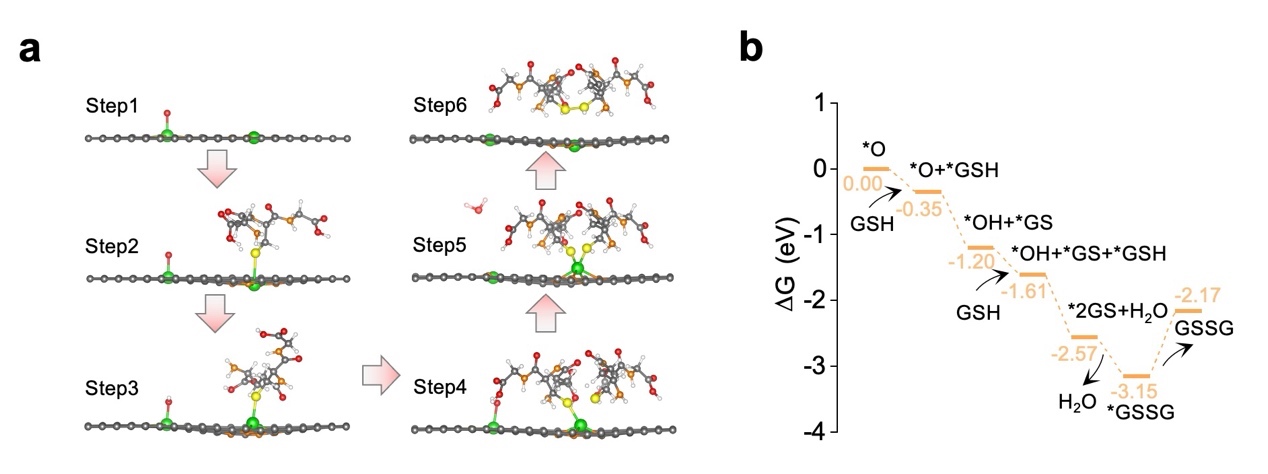


**Fig. S21** a) Proposed catalytic pathway for GSH oxidation on the PtSNC model with highly active Pt=O intermediate, and b) the free energy diagram determined by the DFT calculations


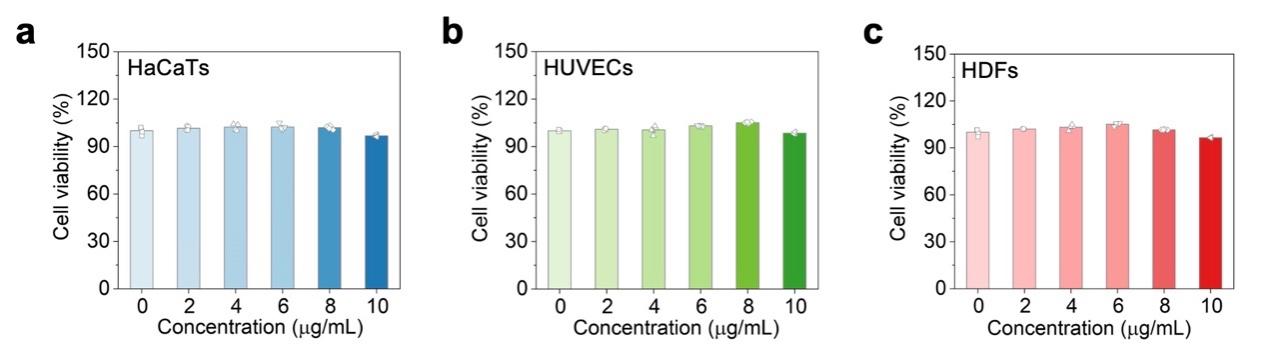


**Fig. S22** Cell viabilities of a) HaCaTs, b) HUVECs, and c) HDFs after incubation with different concentrations of PtSNC for 24 h. Data are expressed as mean ± s.e.m. (*n* = 4 independent experiments)


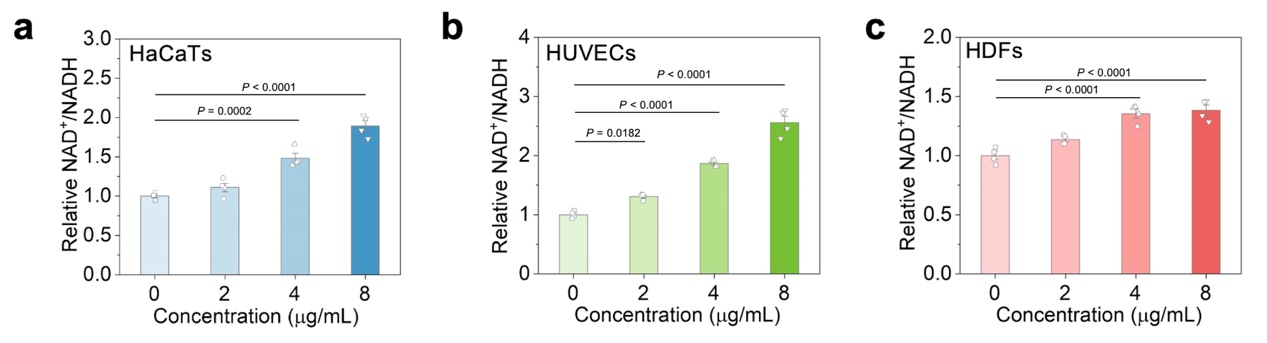


**Fig. S23** The relative NAD^+^/NADH ratios in different kinds of cells including a) HaCaTs, b) HUVECs, and c) HDFs, after incubation with different concentrations of PtSNC for 24 h. Data are expressed as mean ± s.e.m. (*n* = 4 independent experiments). Statistical significance was determined by one-way ANOVA with Tukey’s *post-hoc* test


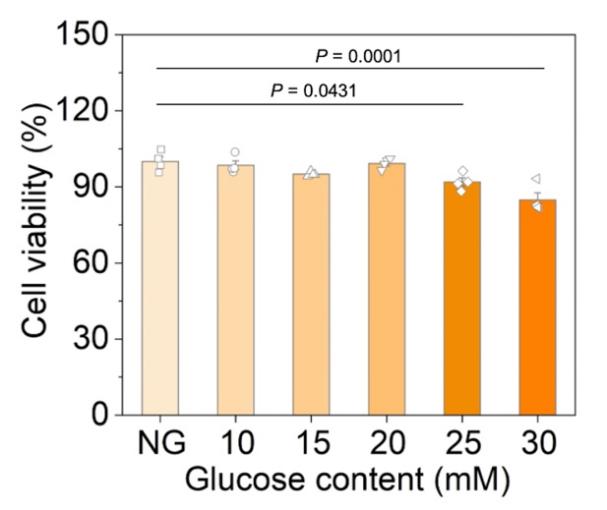


**Fig. S24** Cell viabilities of the HUVECs incubated with different concentrations of glucose for 48 h. Data are expressed as mean ± s.e.m. (*n* = 4 independent experiments).Statistical significance was determined by one-way ANOVA with Tukey’s *post-hoc* test


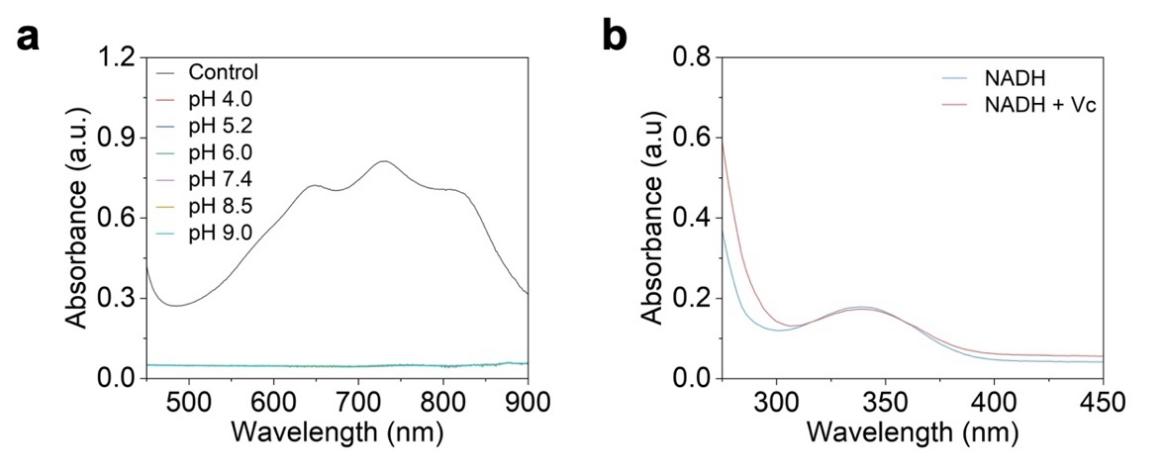


**Fig. S25** a) The total antioxidant capacity of Vc at different pH conditions tested by ABTS radical scavenging assay. b) UV-Vis absorption spectra of NADH before and after 8 h reaction with Vc. The results indicate that Vc possesses potent antioxidant property over a broad pH range (4-9), but has no effect on NADH


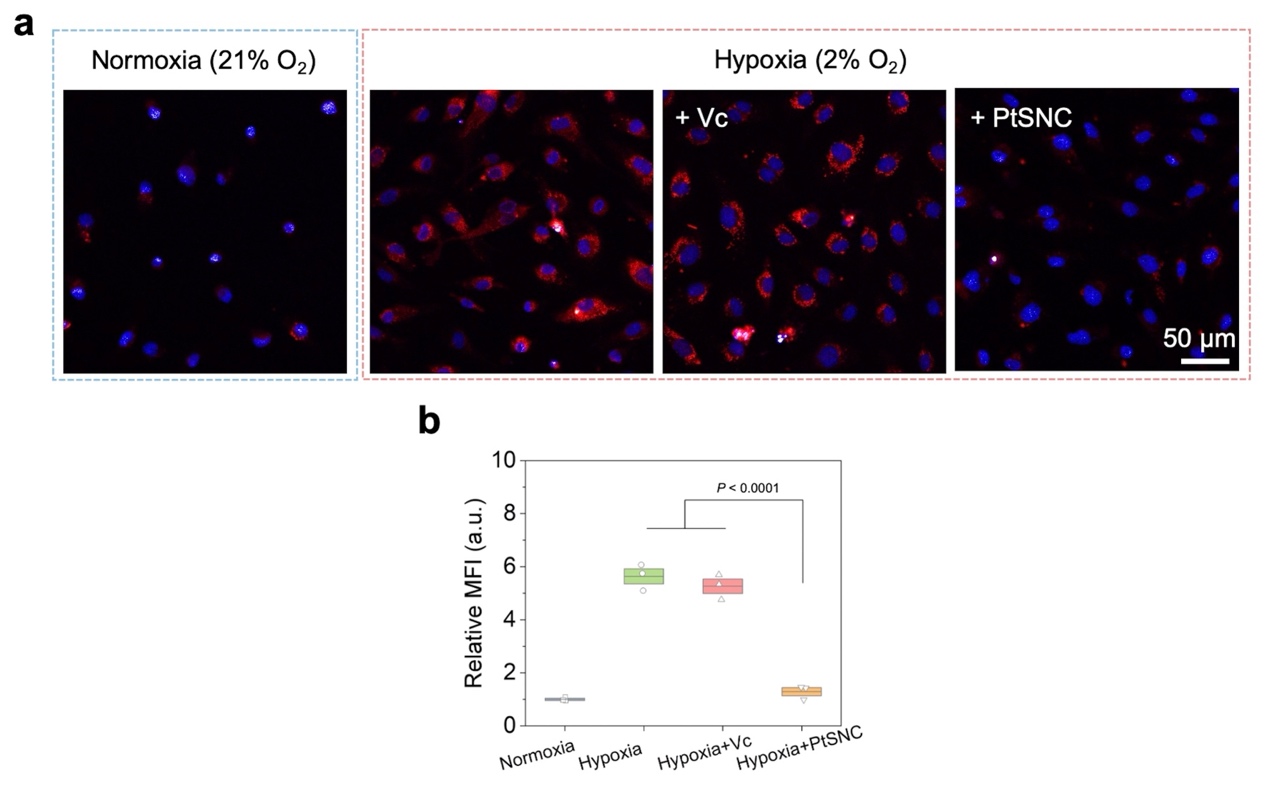


**Fig. S26** Luminescent oxygen sensor [Ru(dpp)_3_]Cl_2_-based O_2_ detection fluorescence a) images and b) intensities. The red fluorescence quenching indicates the alleviation of hypoxia condition and the generation of O_2_ within cells. Data are expressed as mean ± s.e.m. (*n* = 3 independent experiments). Statistical significance was determined by one-way ANOVA with Tukey’s *post-hoc* test

**
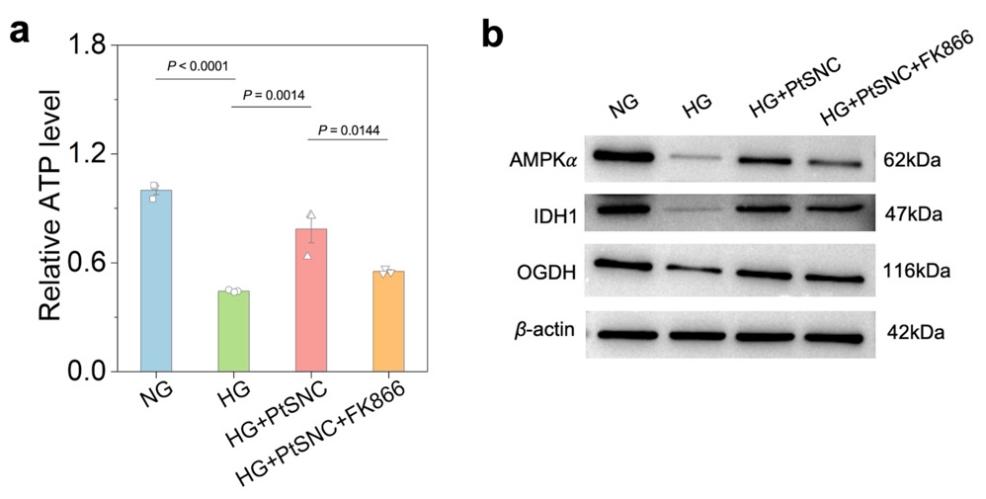
**

**Fig. S27** a) The relative ATP levels in the HUVECs with different incubations. b) Western blot analysis of key proteins involved in energy sensing and production, including AMPK, IDH1, and OGDH, in the HUVECs with different incubations. Data are expressed as mean ± s.e.m. (*n* = 3 independent experiments). Statistical significance was determined by one-way ANOVA with Tukey’s *post-hoc* test


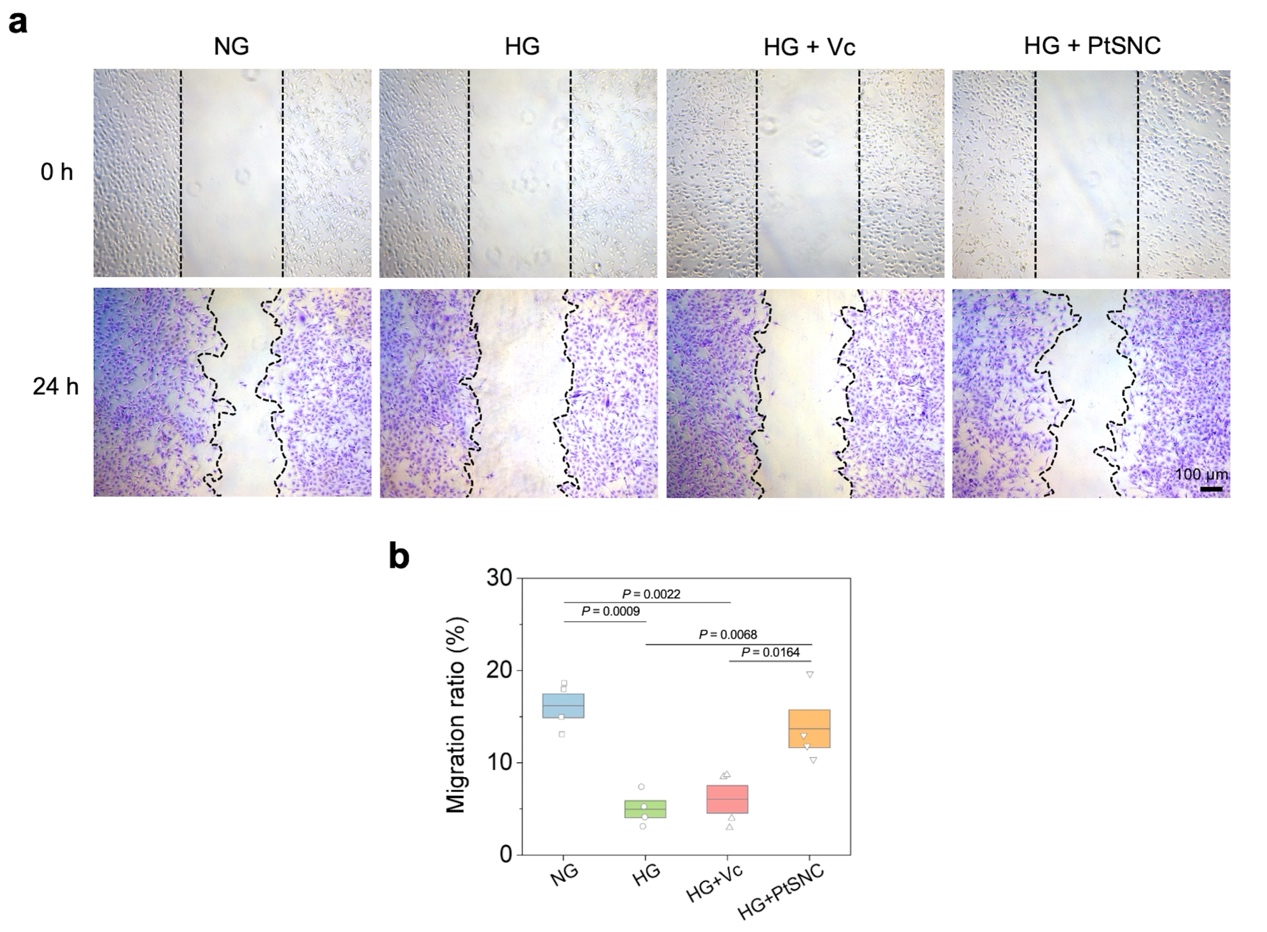


**Fig. S28** a) Cell migration images of HUVECs with different incubations, and b) corresponding migration ratio analysis. Data are expressed as mean ± s.e.m. (*n* = 4 independent experiments). Statistical significance was determined by one-way ANOVA with Tukey’s *post-hoc* test


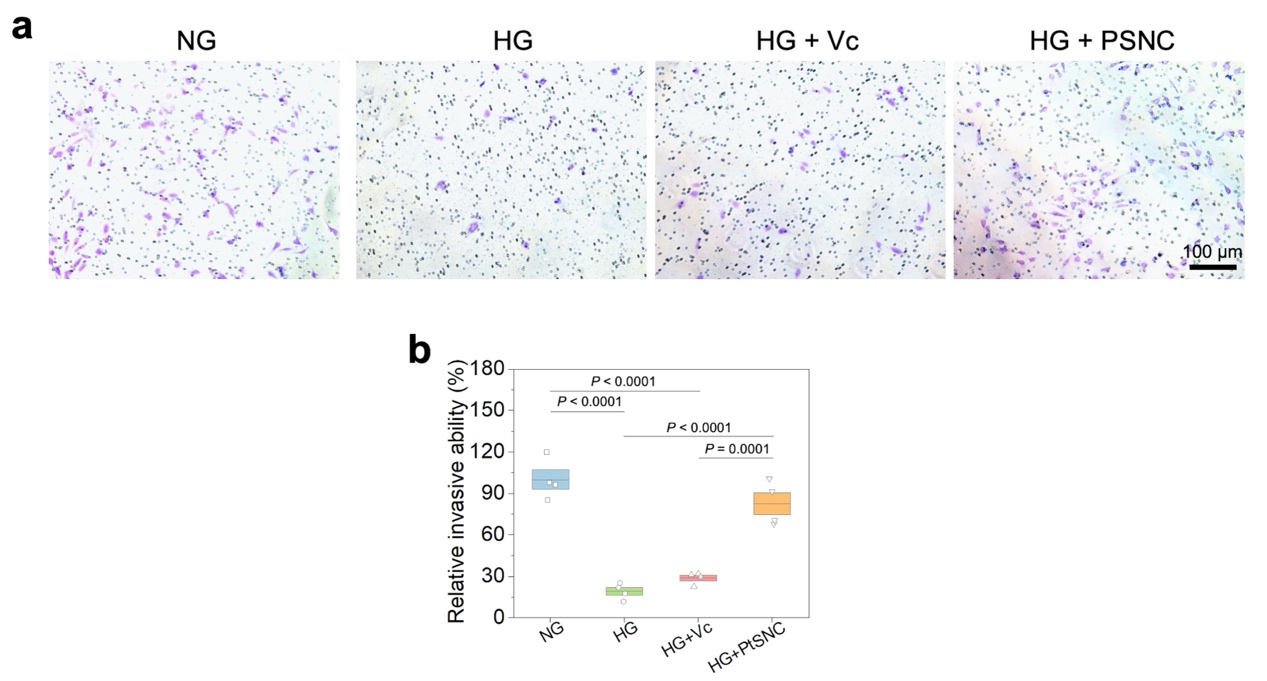


**Fig. S29** a) Cell invasion assay of HUVECs with different incubations using Transwell, and b) corresponding relative invasive ability evaluation. Data are expressed as mean ± s.e.m. (*n* = 4 independent experiments). Statistical significance was determined by one-way ANOVA with Tukey’s *post-hoc* test


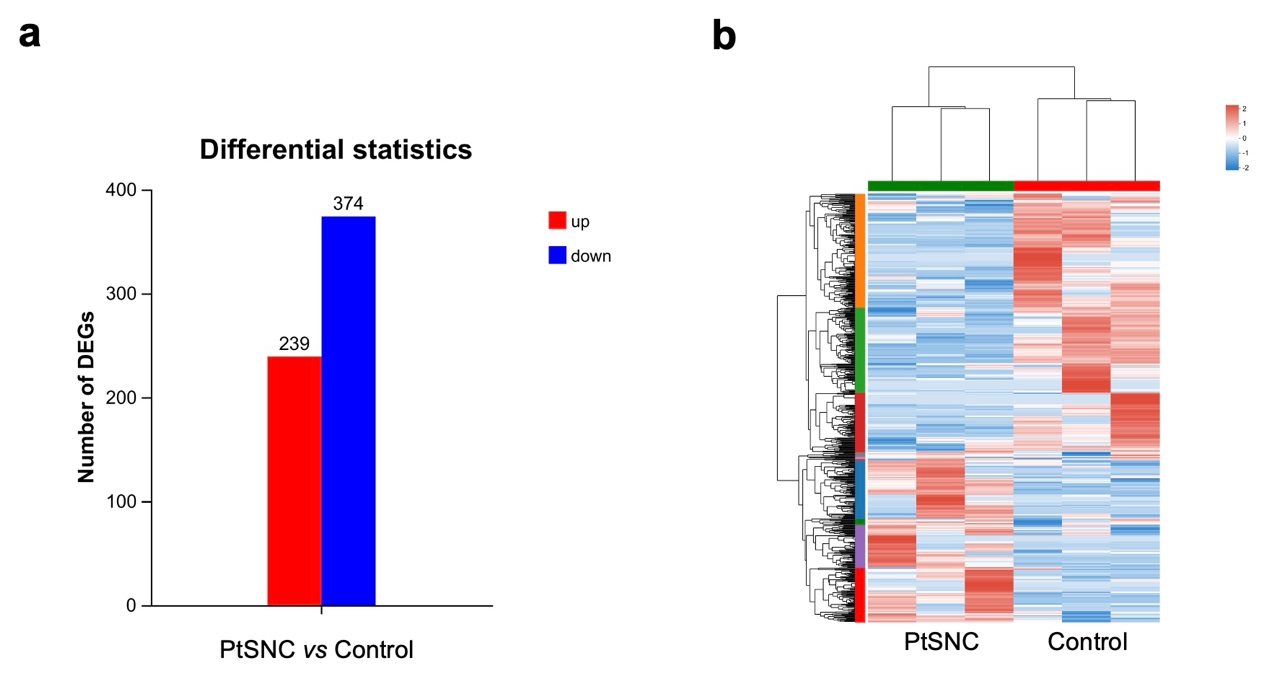


**Fig. S30** a) The differential statistics between the HG-impaired HUVECs with and without PtSNC treatment. b) The heatmap of DEGs (*n* = 3).


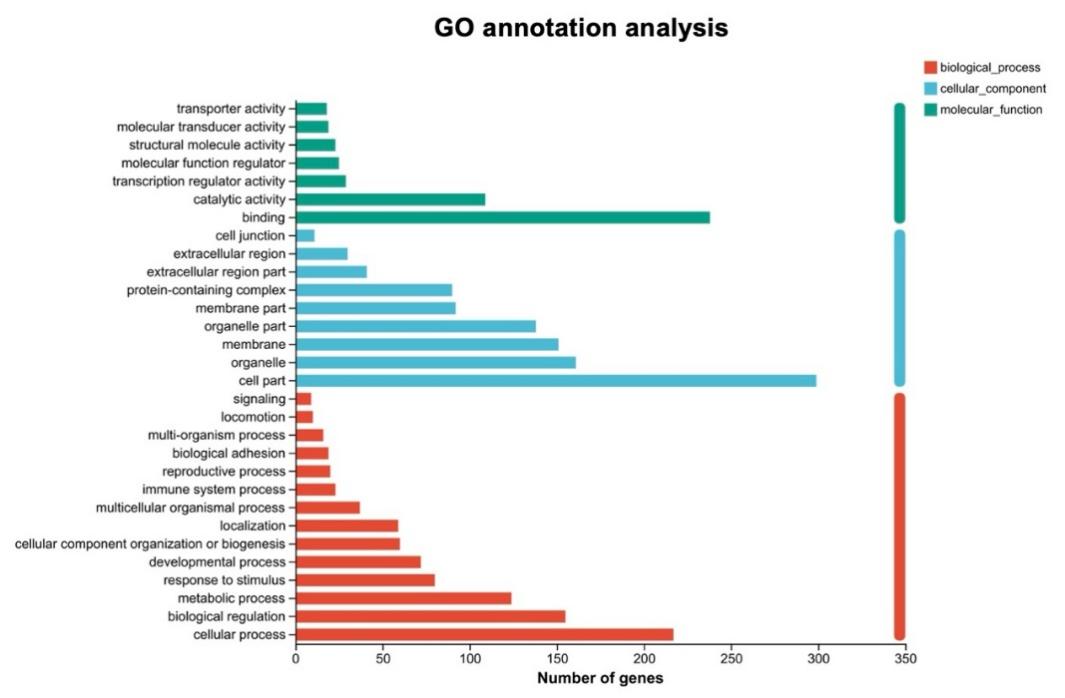


**Fig. S31** GO annotation analysis for presenting the biological process, cellular component, and molecular function associated genes


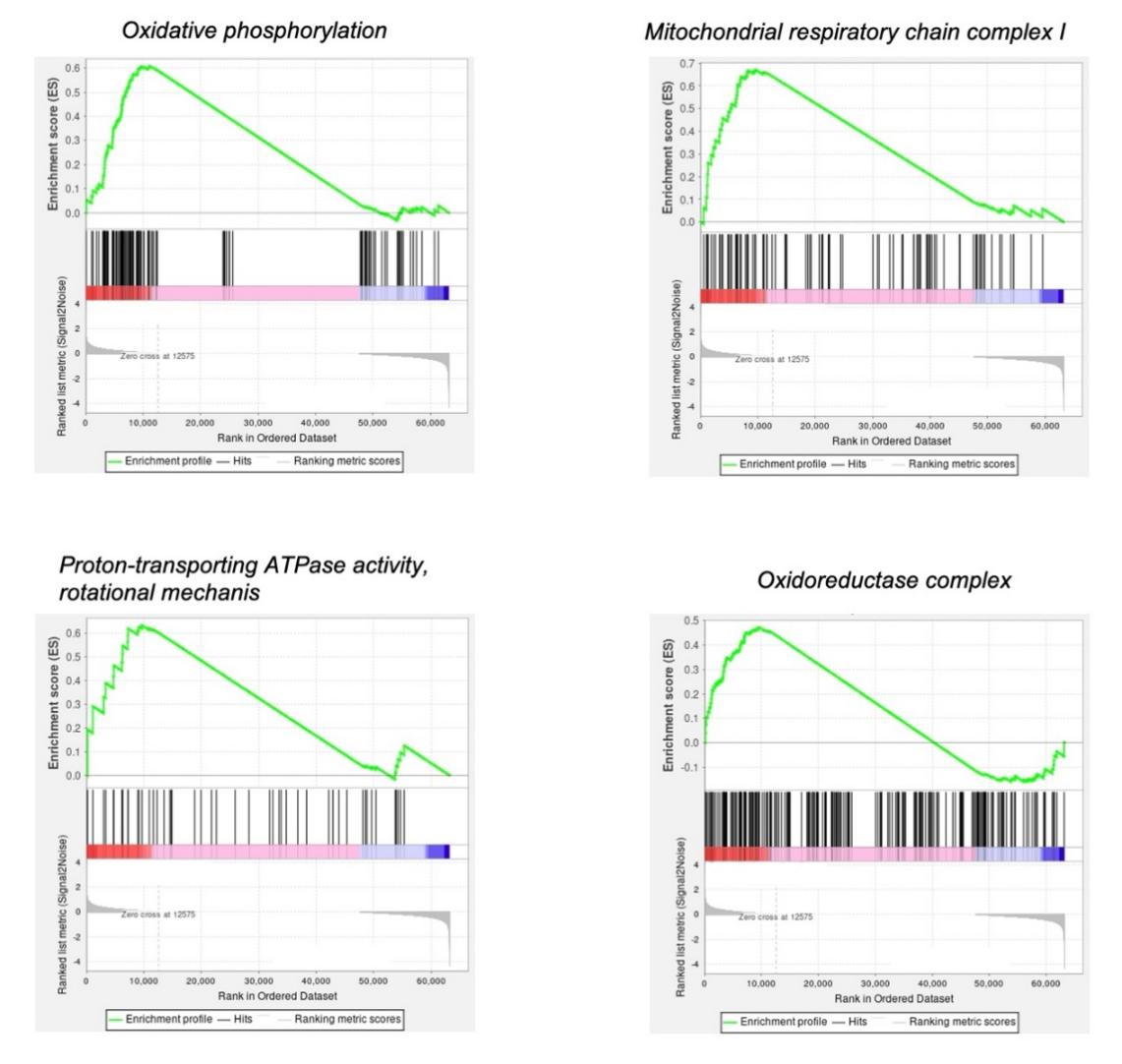


**Fig. S32** GSEA of specific pathways between the HG-impaired HUVECs with and without PtSNC treatment


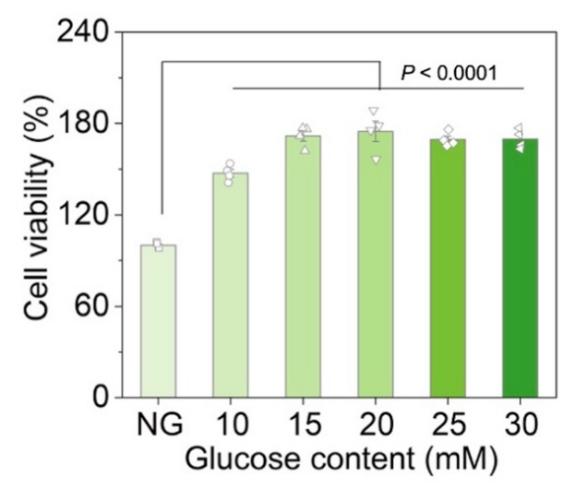


**Fig. S33** a) Cell viabilities of the B16F10 cells incubated with different concentrations of glucose for 48 h. Data are expressed as mean ± s.e.m. (*n* = 4 independent experiments). Statistical significance was determined by one-way ANOVA with Tukey’s *post-hoc* test


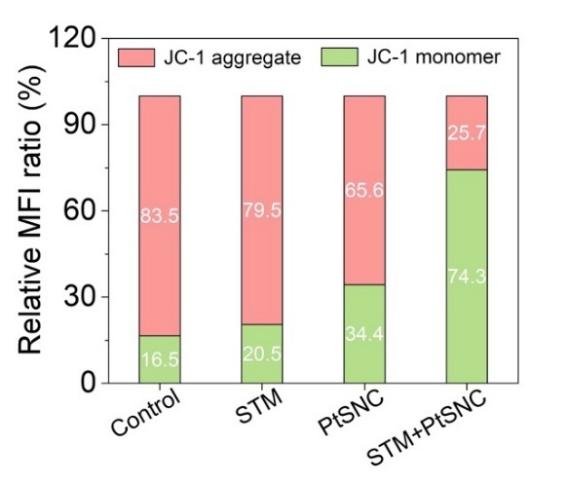


**Fig. S34** Relative fluorescence intensity analysis of JC-1 staining of the B16F10 cells incubated under different conditions, corresponding to **Fig. 6j**. The red fluorescent JC-1 aggregate indicates normal mitochondrial membrane potential, but the green fluorescent JC-1 monomer represents the depolarized mitochondrial membrane

**
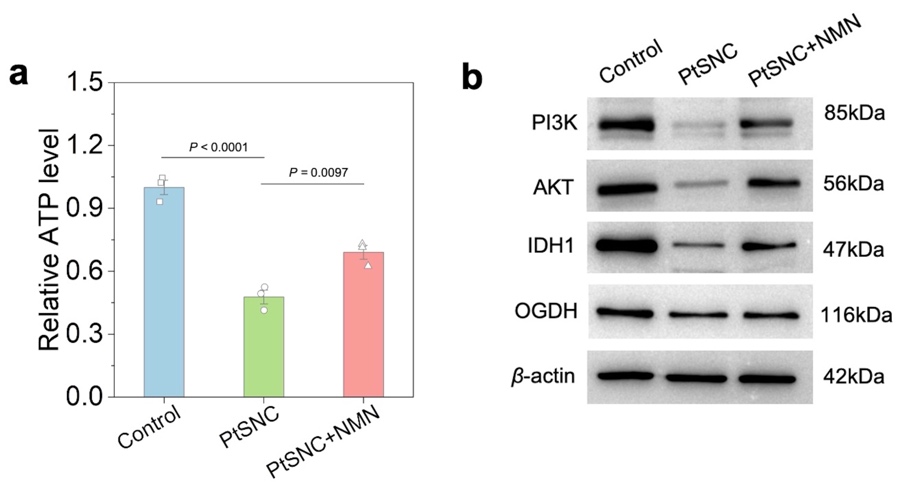
**

**Fig. S35** a) The relative ATP levels in the B16F10 cells with different incubations under STM conditions. b) Western blot analysis of proteins (PI3K, AKT, IDH1, and OGDH) in the B16F10 with different incubations under STM conditions. Data are expressed as mean ± s.e.m. (*n* = 3 independent experiments). Statistical significance was determined by one-way ANOVA with Tukey’s *post-hoc* test


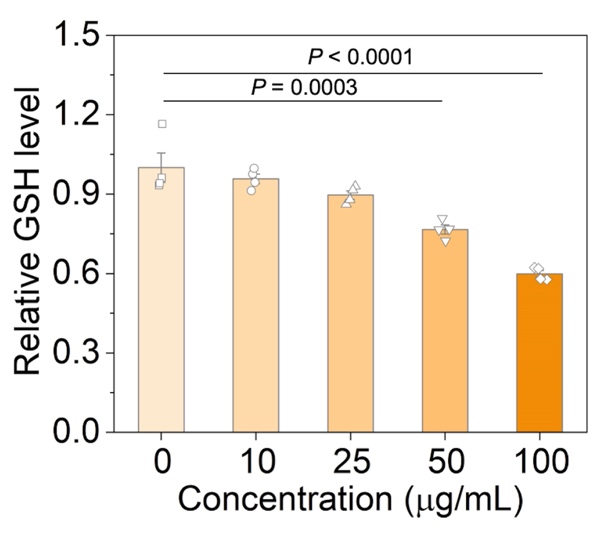


**Fig. S36** The relative GSH levels in the B16F10 cells after incubation with different concentrations of PtSNC. Data are expressed as mean ± s.e.m. (*n* = 4 independent experiments). Statistical significance was determined by one-way ANOVA with Tukey’s *post-hoc* test


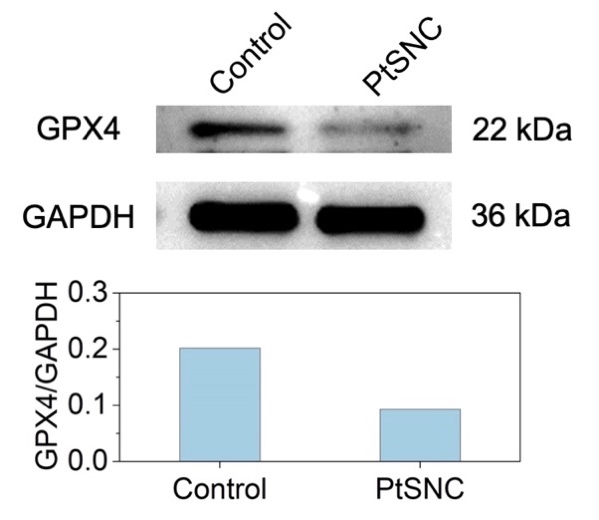


**Fig. S37** Western blot analysis of the GPX4 expressions in the B16F10 cells with and without PtSNC treatment


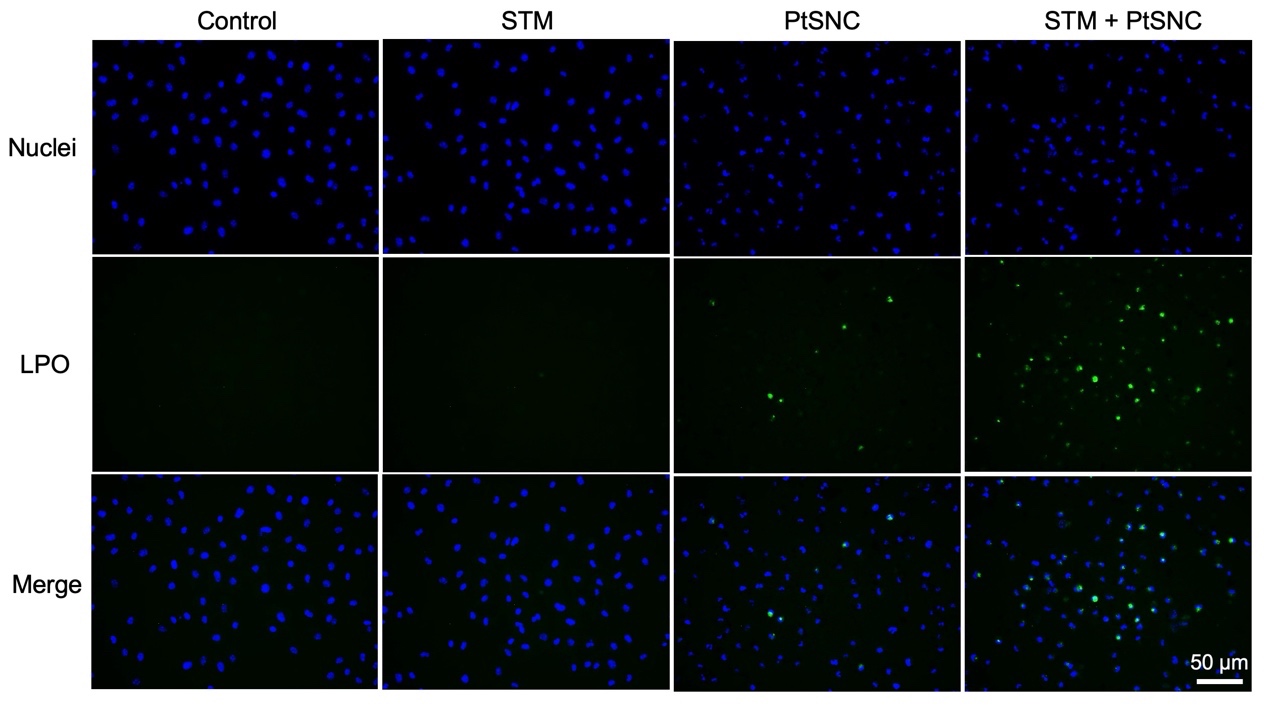


**Fig. S38** Fluorescence images of BODIPY™ 581/591 C11 staining of the B16F10 cells incubated under different conditions. The green fluorescence represents cellular LPO


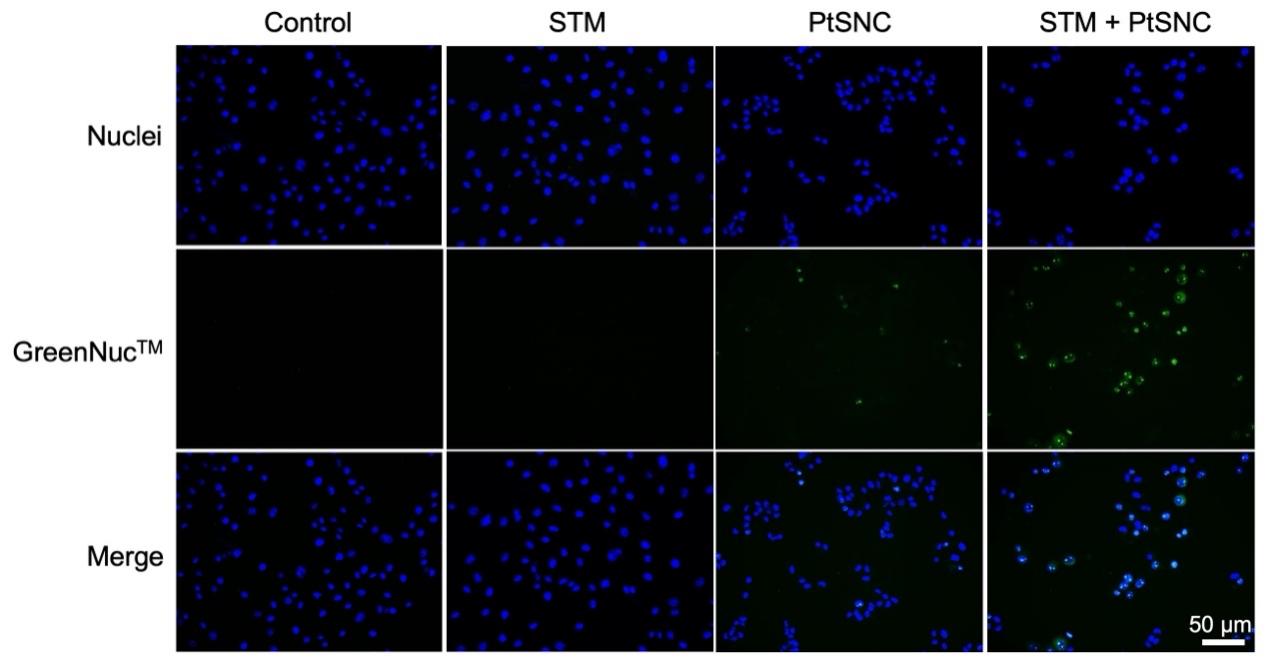


**Fig. S39** Fluorescence images of GreenNuc™ staining of the B16F10 cells incubated under different conditions. The green fluorescence represents the Caspase-3 activation within cells


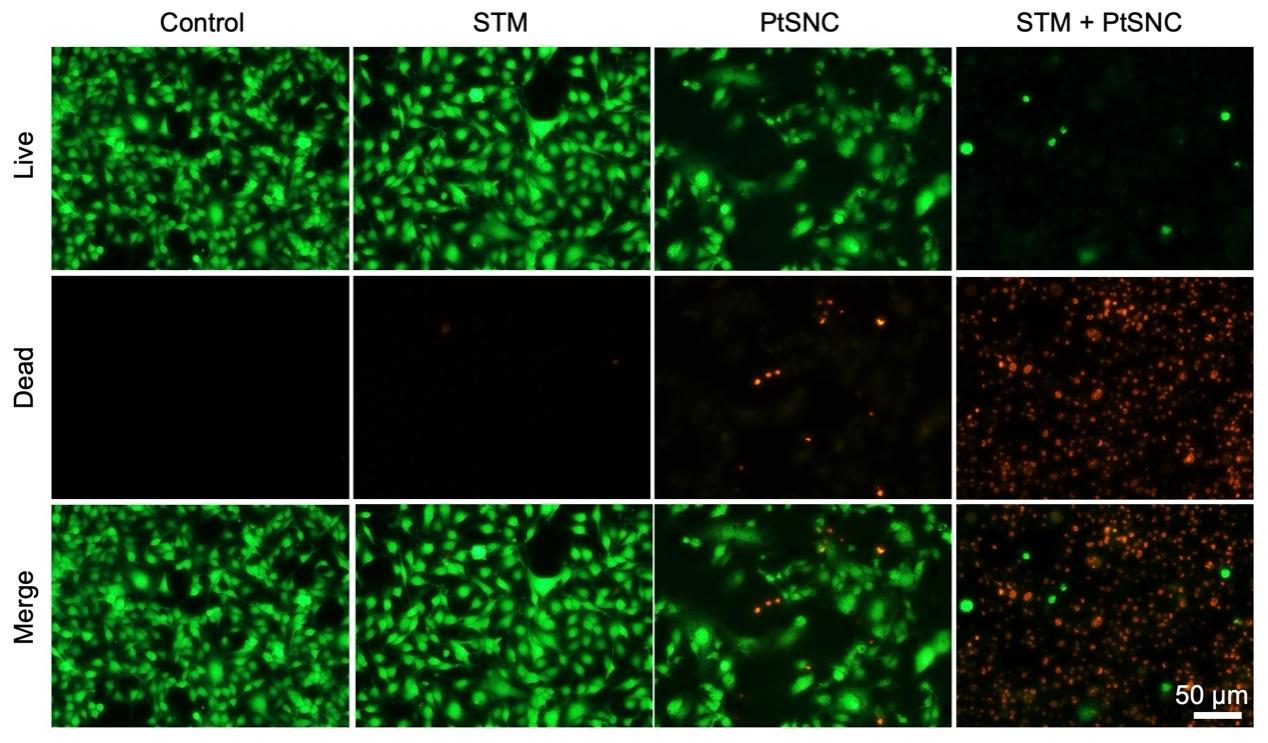


**Fig. S40** Fluorescence images of live/dead cell staining of the B16F10 cells incubated under different conditions. The green fluorescence represents the live cells while the red fluorescence represents the dead cells

**
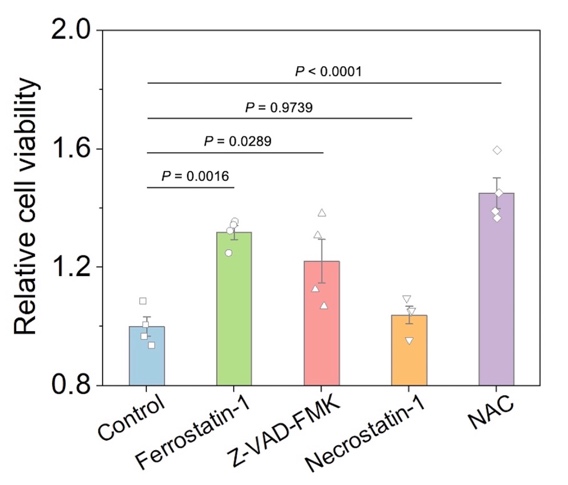
**

**Fig. S41** Relative cell viabilities of the PtSNC-treated B16F10 cells with the additions of different inhibitors. Data are expressed as mean ± s.e.m. (*n* = 4 independent experiments). Statistical significance was determined by one-way ANOVA with Tukey’s *post-hoc* test


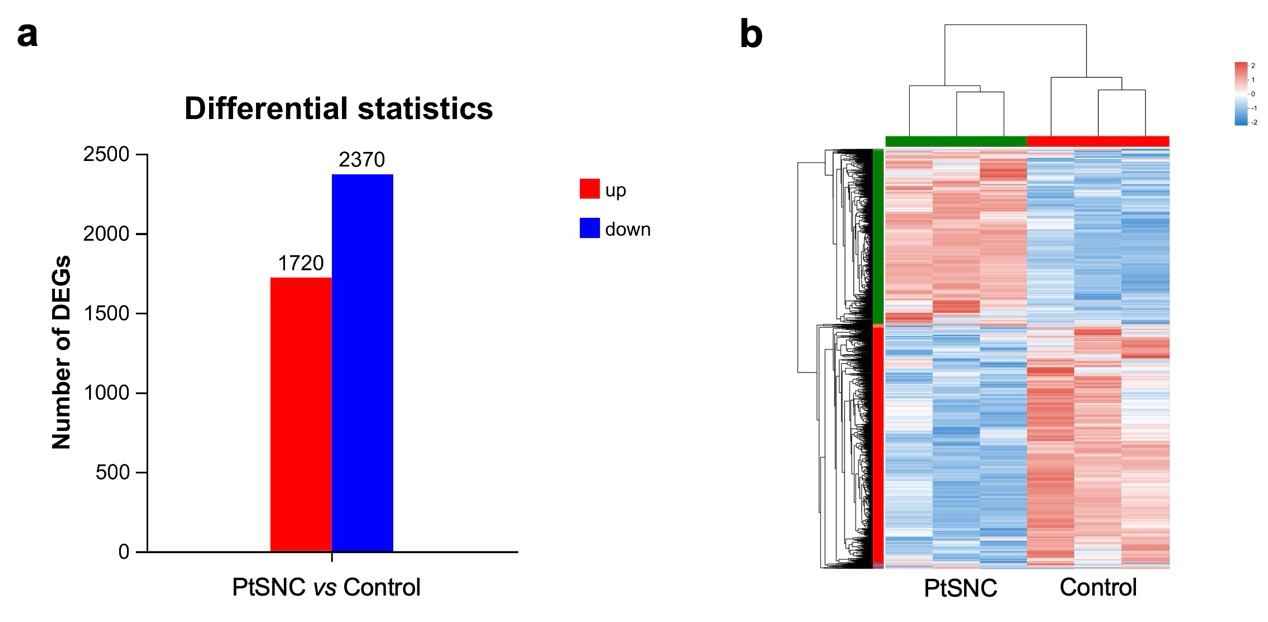


**Fig. S42** a) The differential statistics between the melanoma cells with and without PtSNC treatment. b) The heatmap of DEGs (*n* = 3)


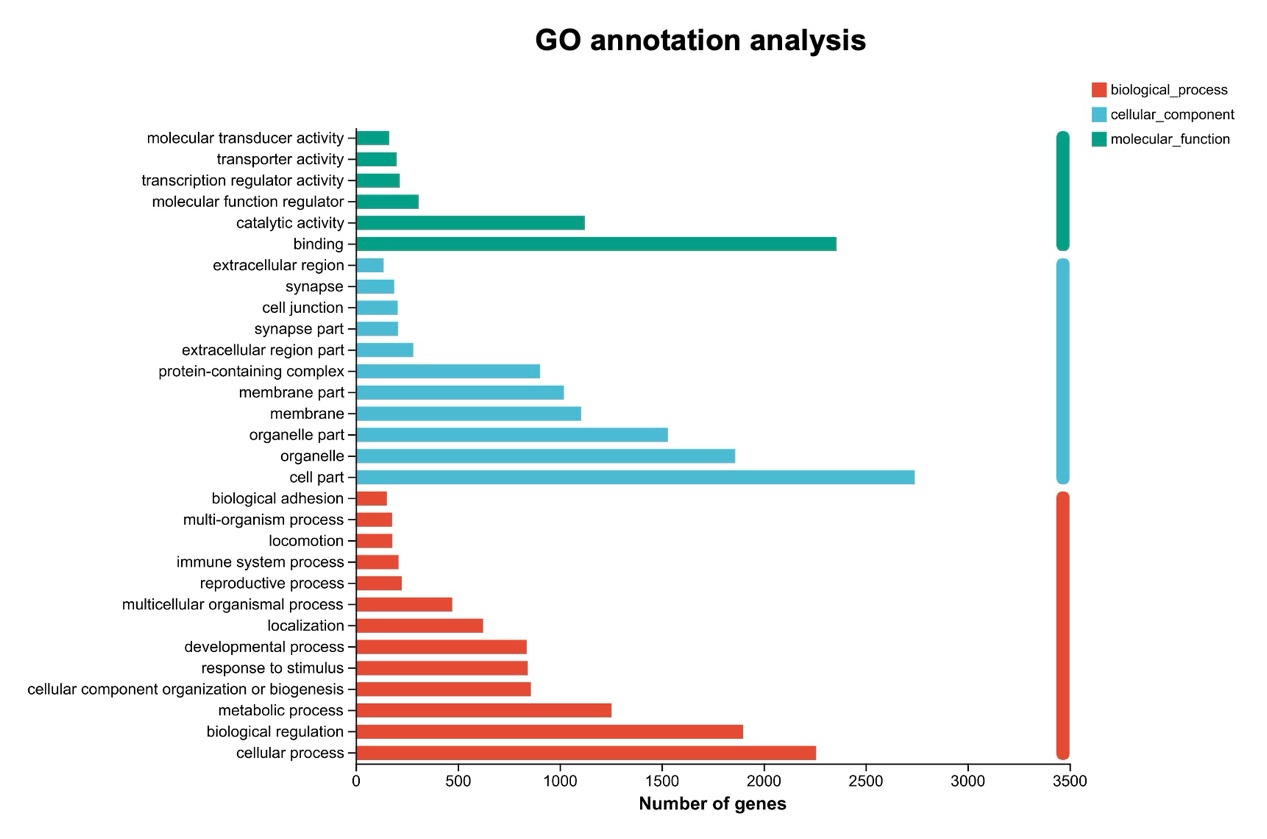


**Fig. S43** GO annotation analysis for presenting the biological process, cellular component, and molecular function associated genes


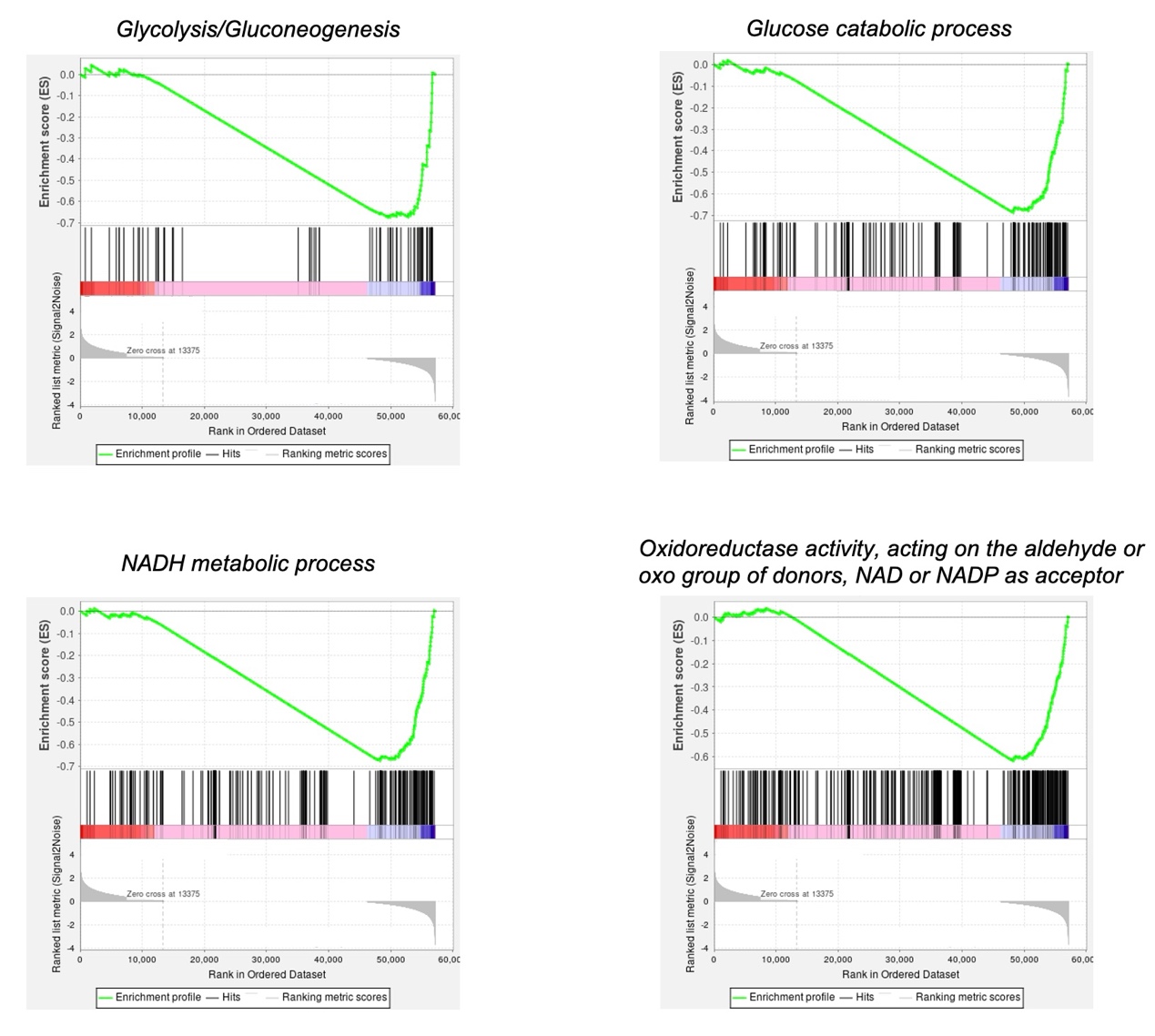


**Fig. S44** GSEA of specific pathways between the melanoma cells with and without PtSNC treatment


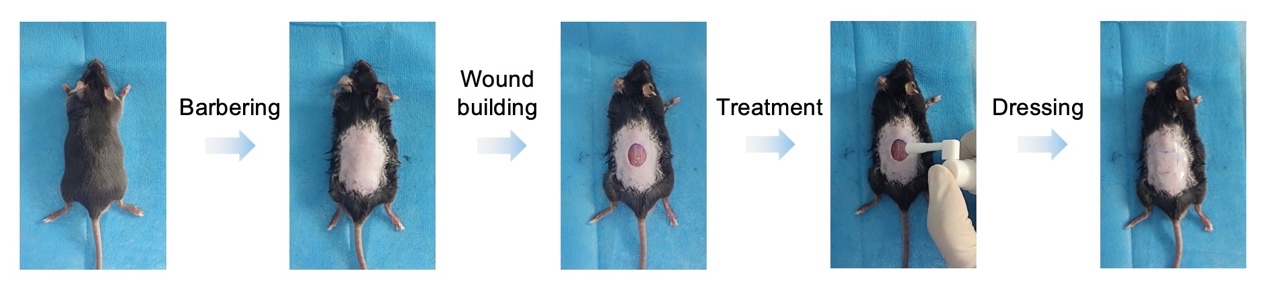


**Fig. S45** Treatment procedures for promoting chronic wound healing on diabetic skin wound-inducing mice


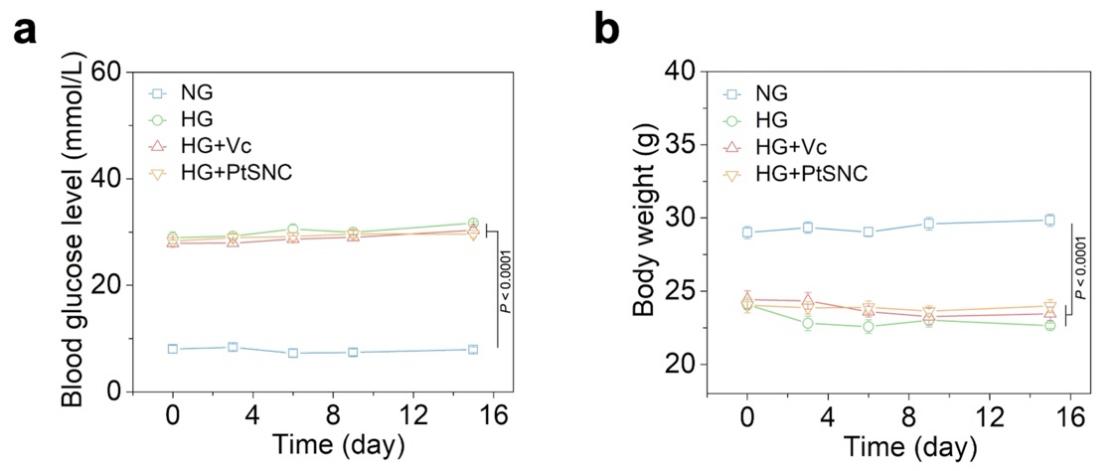


**Fig. S46** a) Blood glucose level and b) body weight changes during different treatments for chronic wound healing. Data are expressed as mean ± s.e.m. (*n* = 5 independent experiments). Statistical significance was determined by two-way ANOVA with Bonferroni’s *post-hoc* test


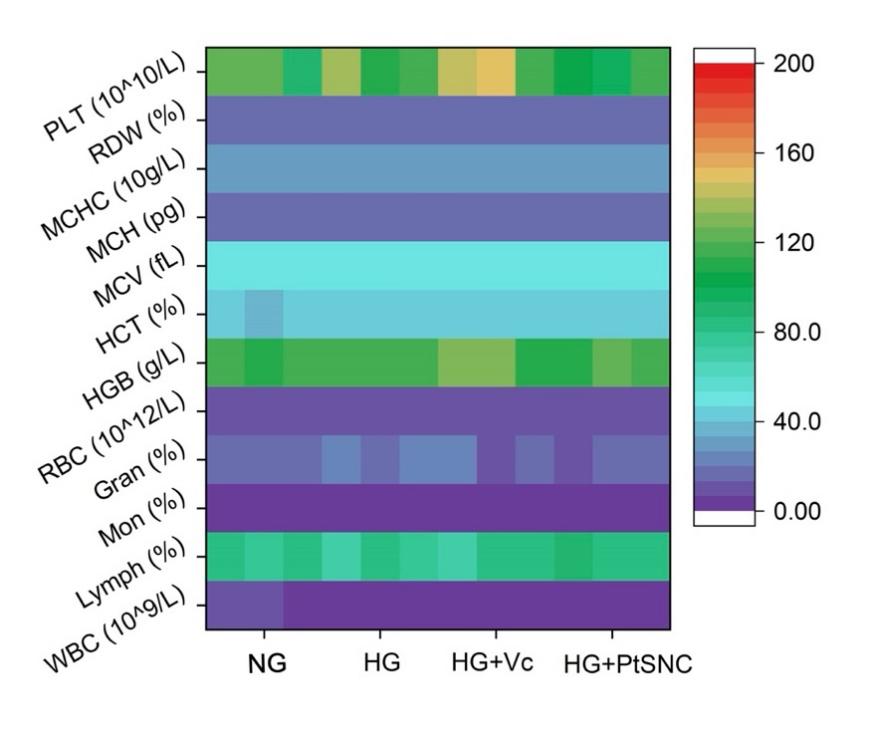


**Fig. S47** Blood-related parameter analysis of the diabetic skin wound-inducing mice on day 15 post-different treatments. PLT: platelet count; RDW: red cell distribution width; MCHC: mean corpuscular hemoglobin concentration; MCH: mean red blood cell hemoglobin; MCV: mean red blood cell volume; HCT: haematocrit; HGB: hemoglobin; RBC: red blood cell count; Gran: percentage of neutrophils; Mon: percentage of monocytes; Lymph: percentage of lymphocytes; WBC: white blood cell count. Data are expressed as mean ± s.e.m. (*n* = 3 independent experiments)


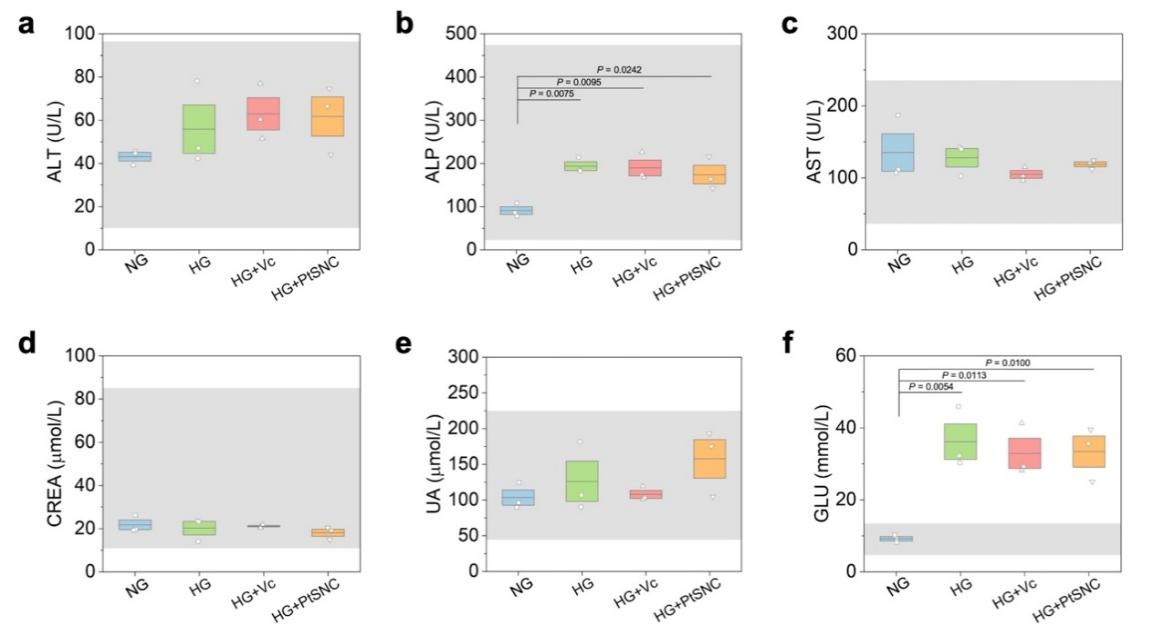


**Fig. S48** a-c) Liver (ALT: alanine aminotransferase; ALP: alkaline phosphatase; AST: aspartate aminotransferase) function, d,e) kidney (CREA: creatinine; UA: uric acid) function, and f) blood glucose analyses of the diabetic skin wound-inducing mice on day 15 post-different treatments. Lighter gray bars represent the range of normal values obtained from the healthy mice. All the liver/kidney function parameters in these groups were within the normal range while the blood glucose levels in the diabetic groups were much higher than the non-diabetic group. The result indicates the high biosafety of PtSNC. Compared with the non-diabetic group, several liver/kidney function parameters in the diabetic groups were slightly higher, meaning that hyperglycemia affects the liver/kidney functions which is consistent with the symptoms of metabolic diseases. Data are expressed as mean ± s.e.m. (*n* = 3 independent experiments). Statistical significance was determined by one-way ANOVA with Tukey’s *post-hoc* test


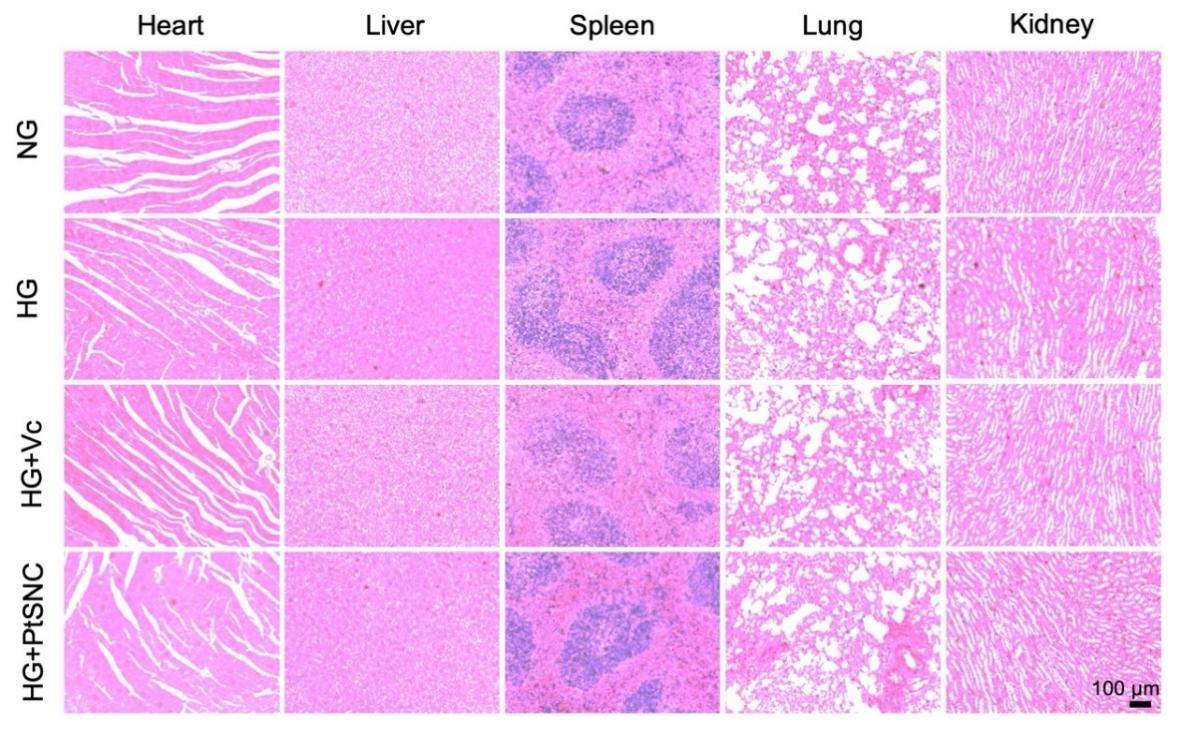


**Fig. S49** H&E staining images of the tissues (heart, liver, spleen, lung, and kidney) obtained from the diabetic skin wound-inducing mice post-different treatments


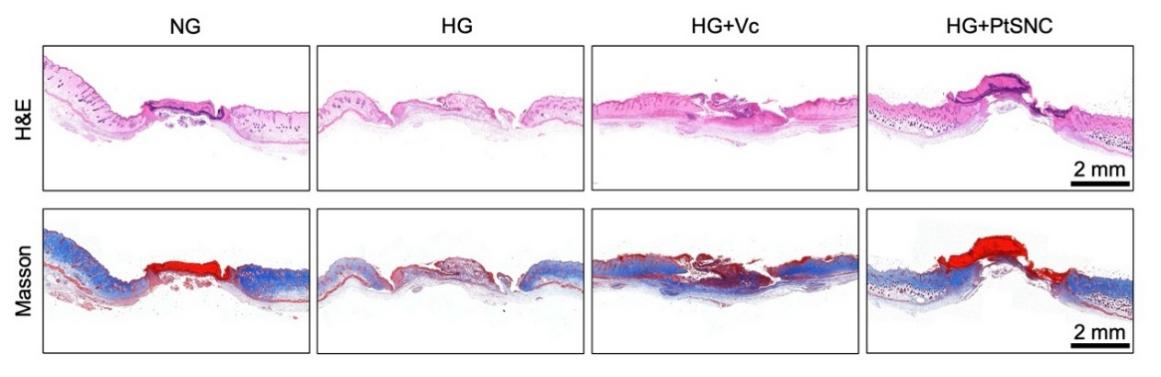


**Fig. S50** H&E/Masson staining images of the wound skin tissues harvested at day 6


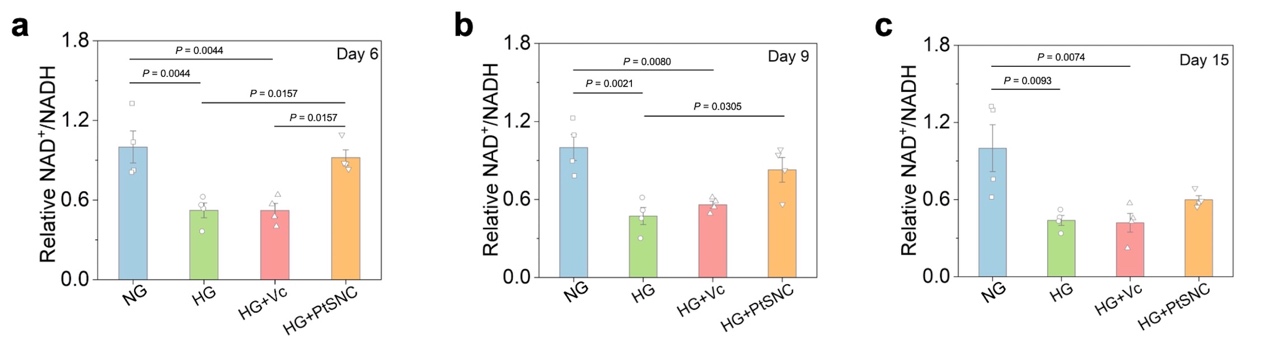


**Fig. S51** The relative NAD^+^/NADH ratios in the newly formed skin tissues in the wounds harvested at a) day 6, b) day 9, and c) day15. Data are expressed as mean ± s.e.m. (*n* = 4 independent experiments). Statistical significance was determined by one-way ANOVA with Tukey’s *post-hoc* test


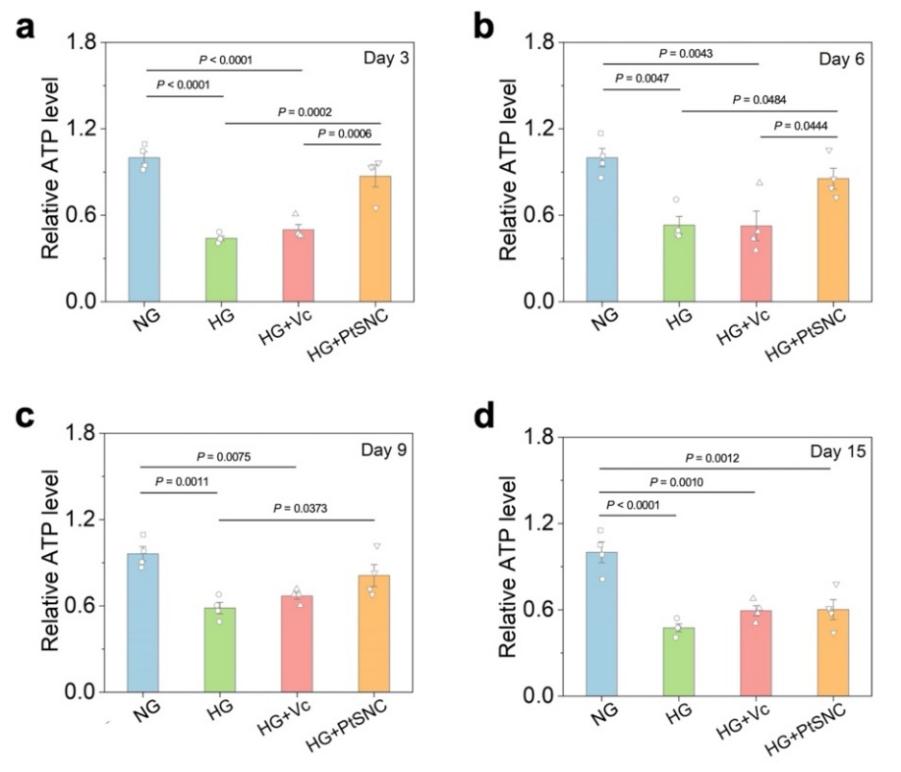


**Fig. S52** The relative ATP levels in the newly formed skin tissues in the wounds harvested at a) day 3, b) day 6, c) day 9, and d) day15. Data are expressed as mean ± s.e.m. (*n* = 4 independent experiments). Statistical significance was determined by one-way ANOVA with Tukey’s *post-hoc* test.

**
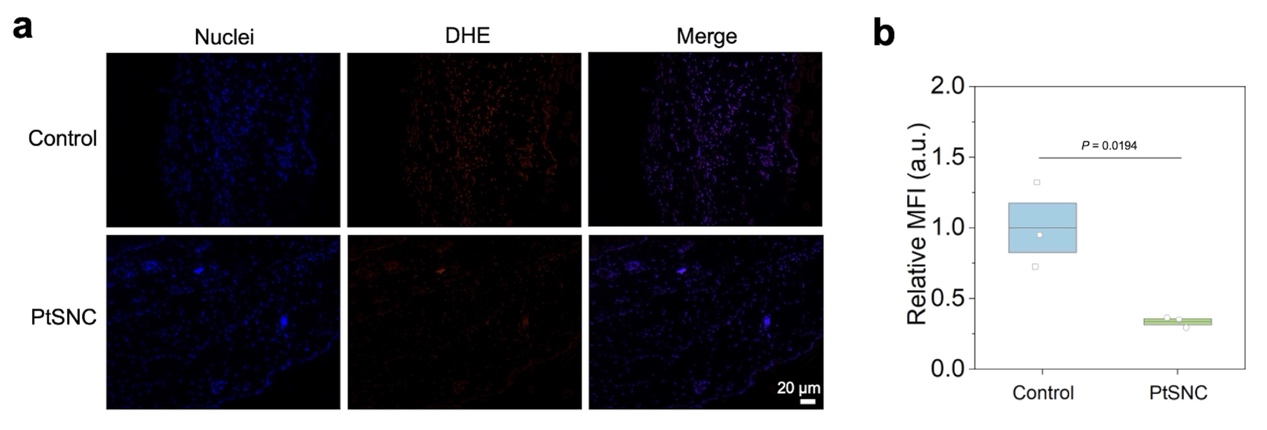
**

**Fig. S53** a) Fluorescence images for DHE-based ROS (O_2_•^-^) detection in the diabetic wound sections and b) corresponding intensity analysis. Data are expressed as mean ± s.e.m. (*n* = 3 independent experiments). Statistical significance was determined by two-tailed Student’s *t*-test


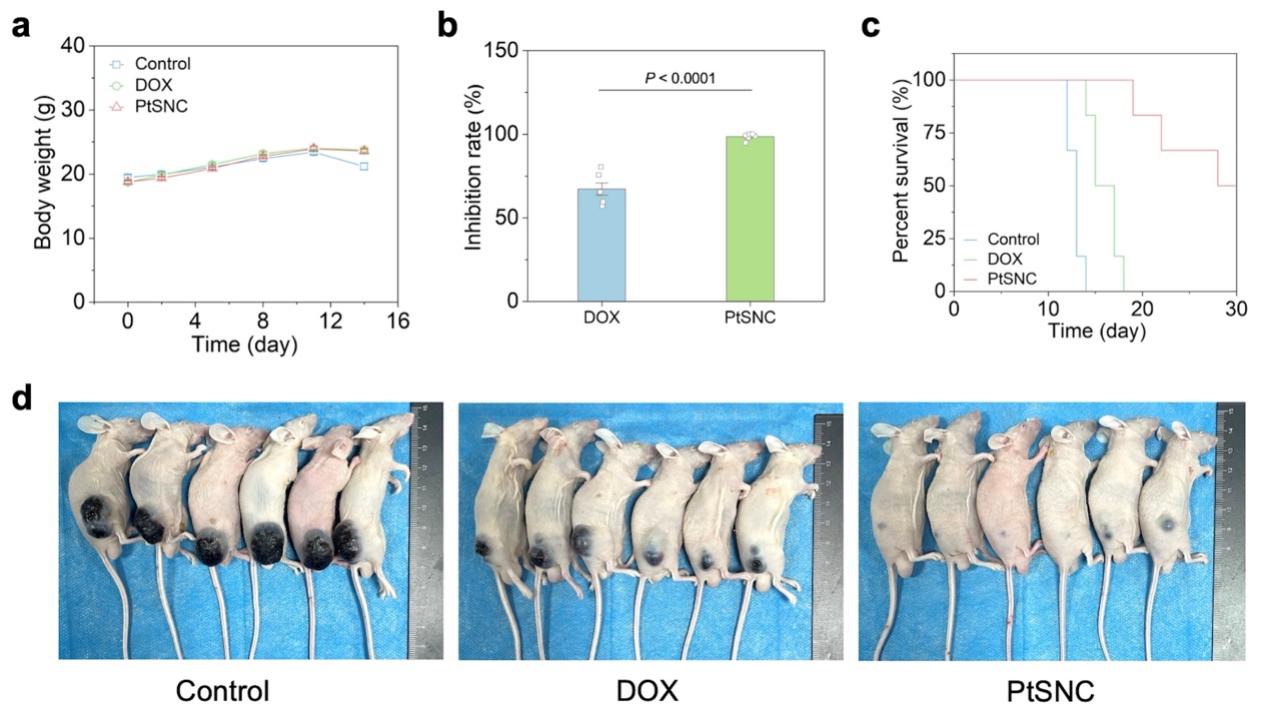


**Fig. S54** a) Body weight changes during different anti-tumor treatments. b) Calculated tumor inhibition rates for the DOX and PtSNC treatment groups. c) Survival curves of the mice in different groups. d) Photographs of these melanoma-bearing mice post-different treatments. Data are expressed as mean ± s.e.m. (*n* = 6 independent experiments). Statistical significance was determined by two-tailed Student’s *t*-test.


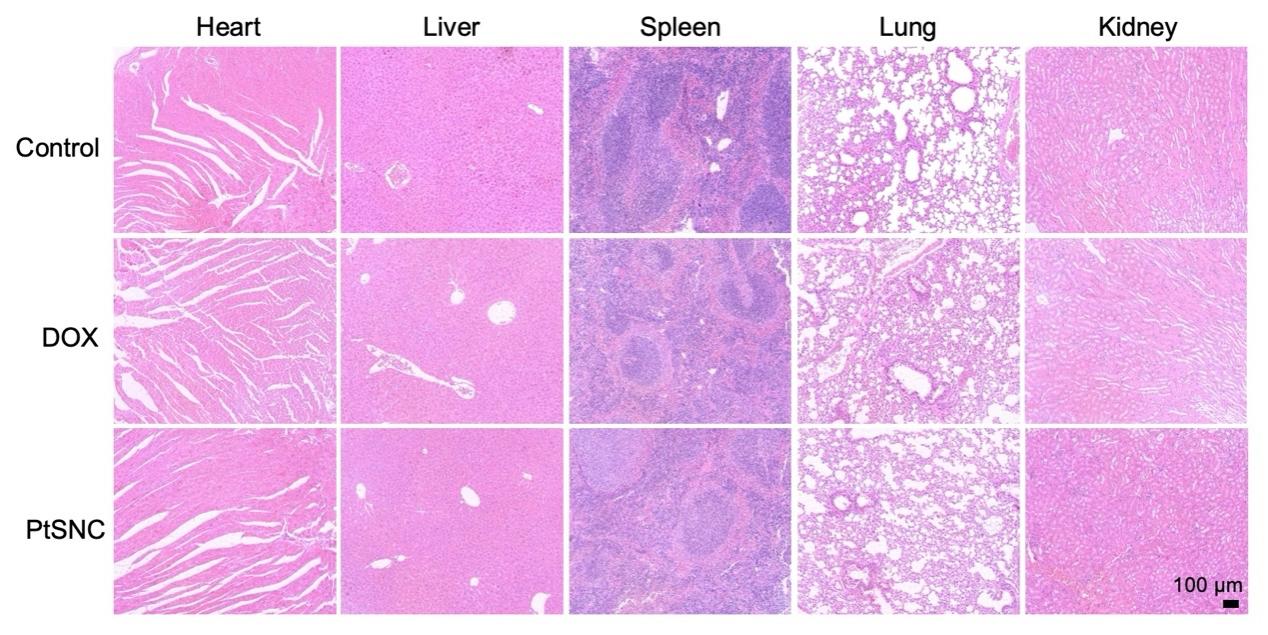


**Fig. S55** H&E staining images of the tissues (heart, liver, spleen, lung, and kidney) obtained from the melanoma-bearing mice post-different treatments


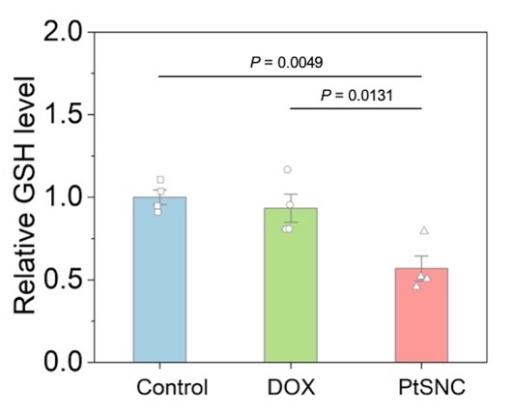


**Fig. S56** The relative GSH levels in the tumors at 48 h post-different treatments. Data are expressed as mean ± s.e.m. (*n* = 4 independent experiments). Statistical significance was determined by one-way ANOVA with Tukey’s *post-hoc* test

**
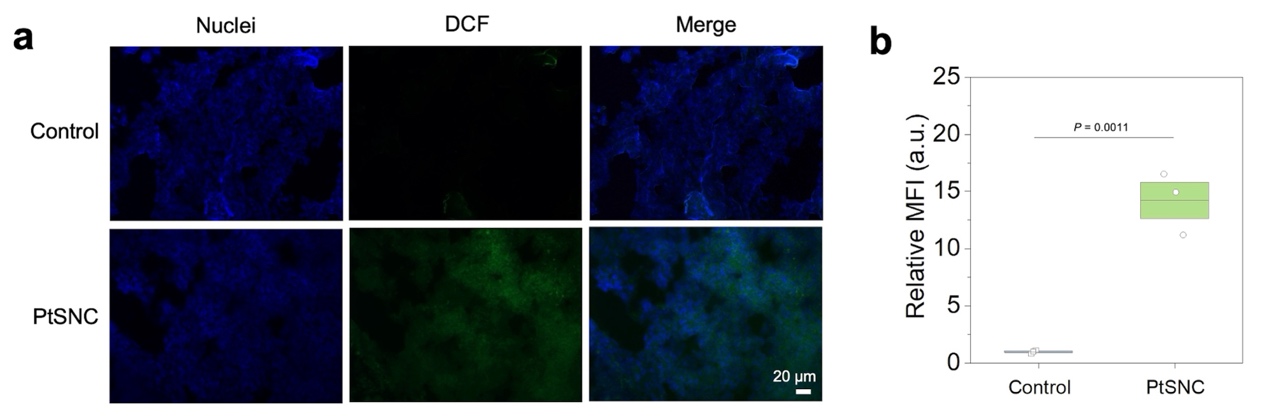
**

**Fig. S57** a) Fluorescence images for DCFH-DA staining in the tumor sections and b) corresponding intensity analysis. Data are expressed as mean ± s.e.m. (*n* = 3 independent experiments). Statistical significance was determined by two-tailed Student’s *t*-test.


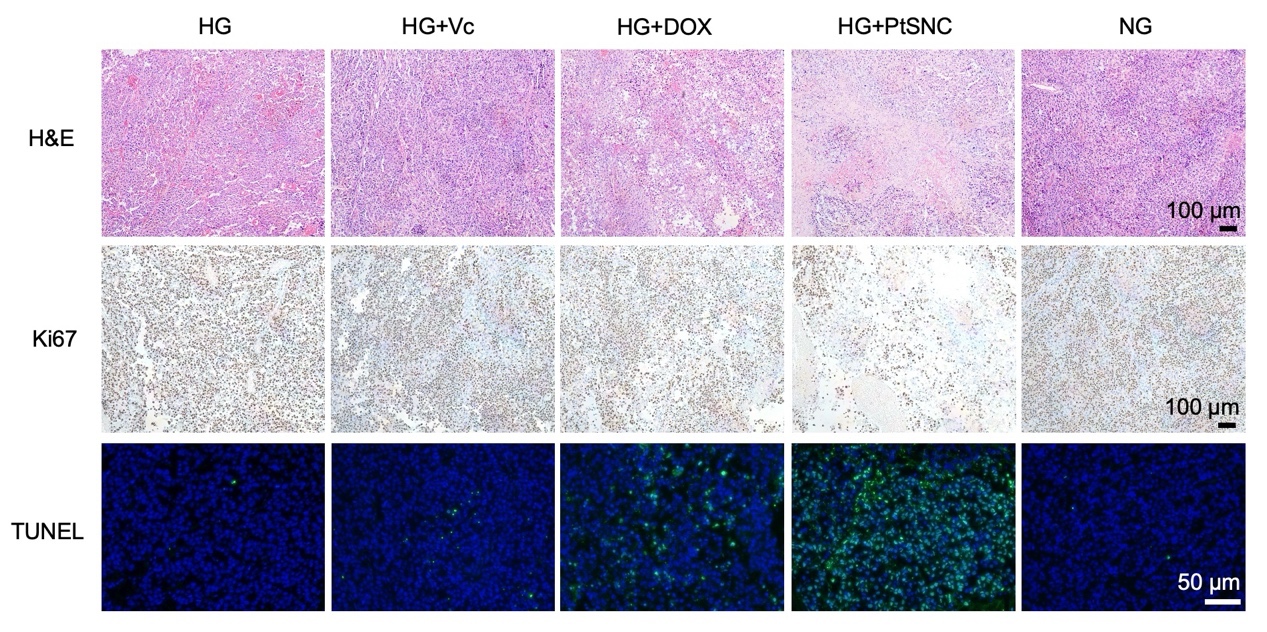


**Fig. S58** H&E/Ki67/TUNEL staining analyses of the recurrent tumor tissues in the operative mice post-different treatments


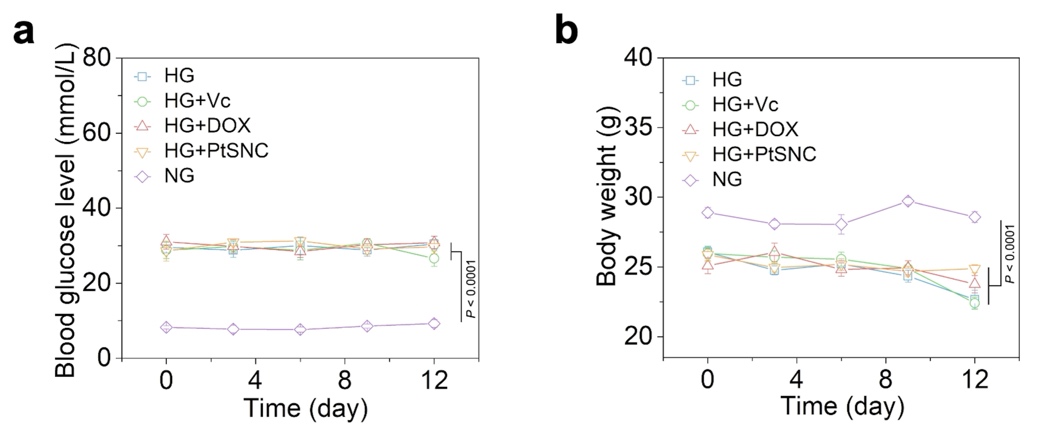


**Fig. S59** a) Blood glucose level and b) body weight changes during different treatments for simultaneous tumor recurrence inhibition and wound healing acceleration. Data are expressed as mean ± s.e.m. (*n* = 5 independent experiments). Statistical significance was determined by two-way ANOVA with Bonferroni’s *post-hoc* test.


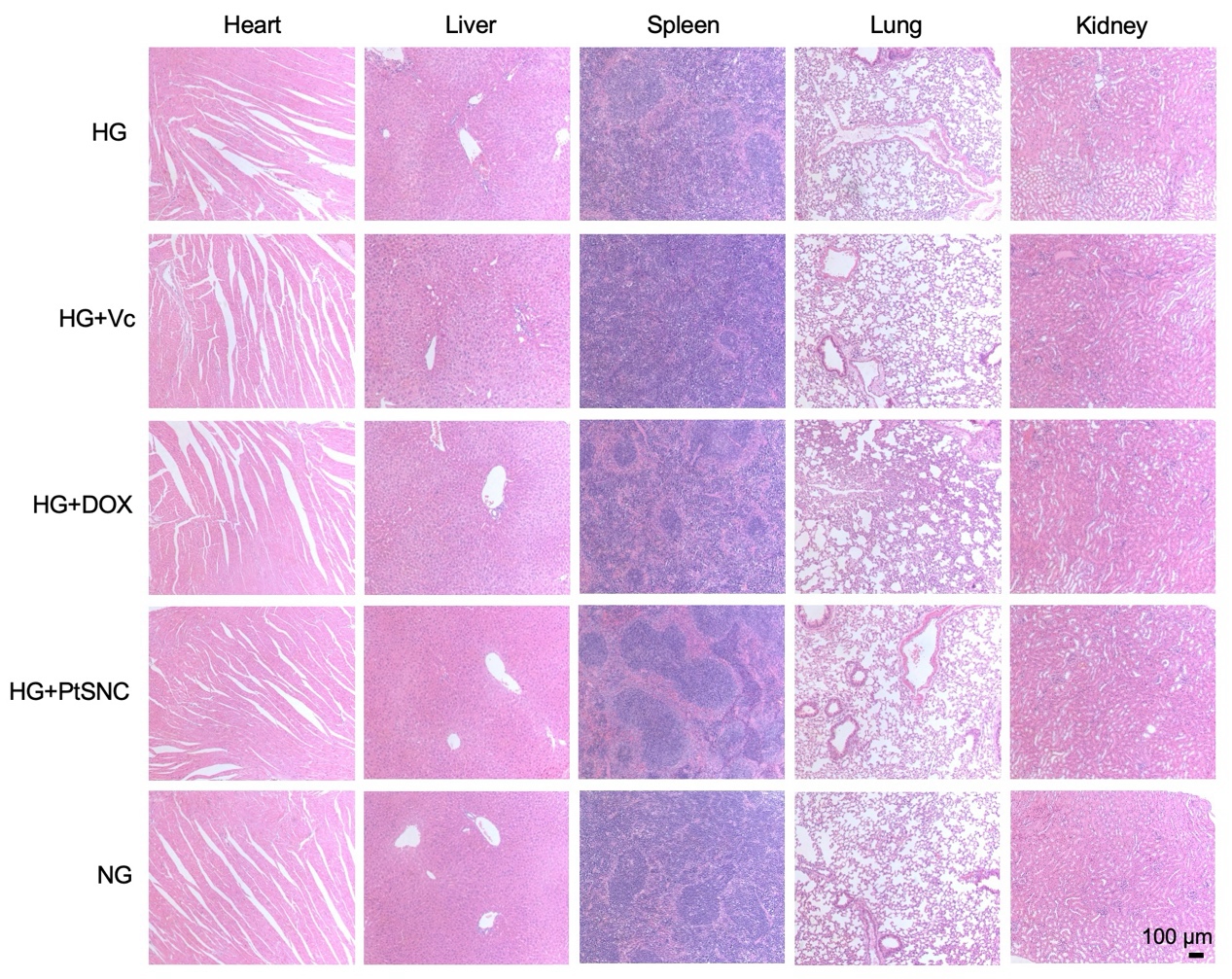


**Fig. S60** H&E staining images of the tissues (heart, liver, spleen, lung, and kidney) obtained from the diabetic tumor resection mice post-different treatments

**
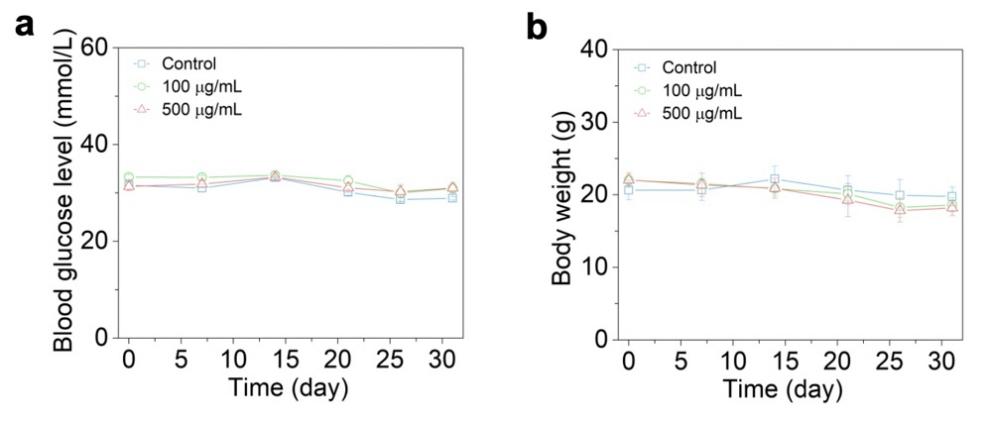
**

**Fig. S61** a) Blood glucose level and b) body weight changes during the long-term safety assessment in diabetic mice receiving repeat intravenous doses of PtSNC. Data are expressed as mean ± s.e.m. (*n* = 3 independent experiments)

**
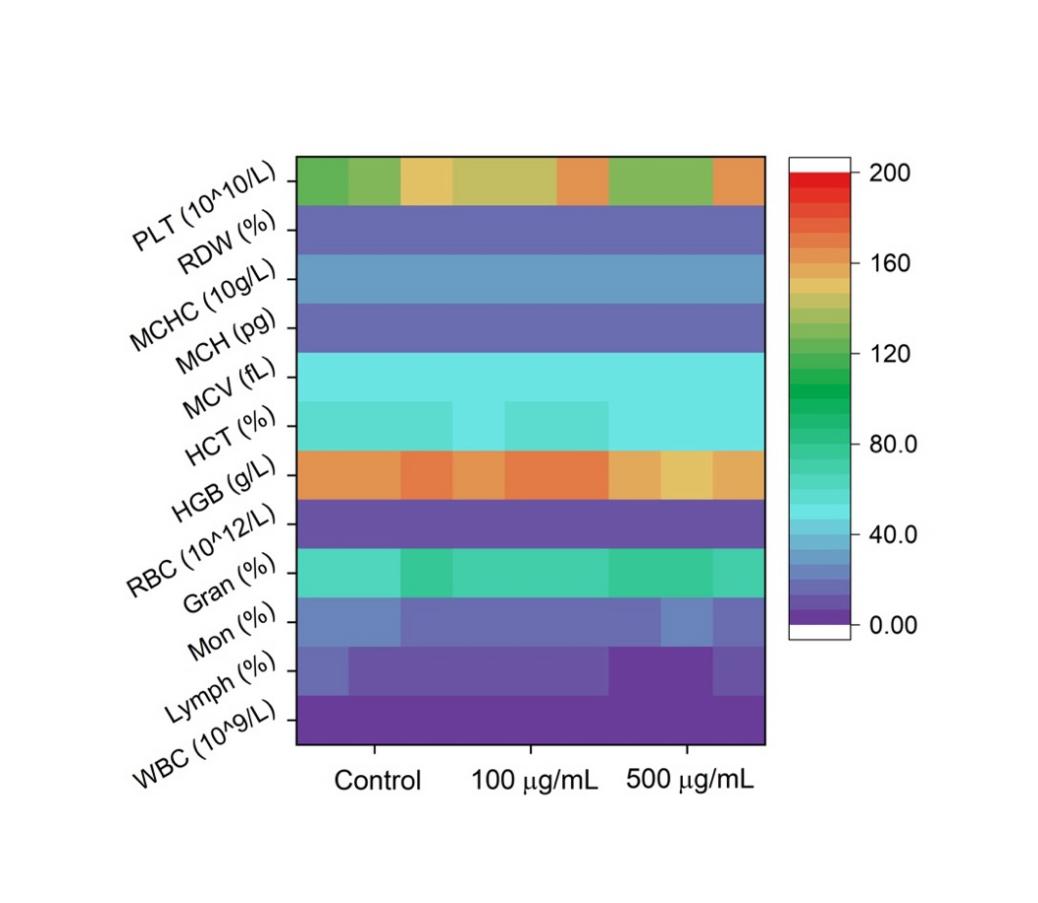
**

**Fig. S62** Blood-related parameter analysis of the diabetic mice on day 31 post-systemic PtSNC injections. Data are expressed as mean ± s.e.m. (*n* = 3 independent experiments)

**
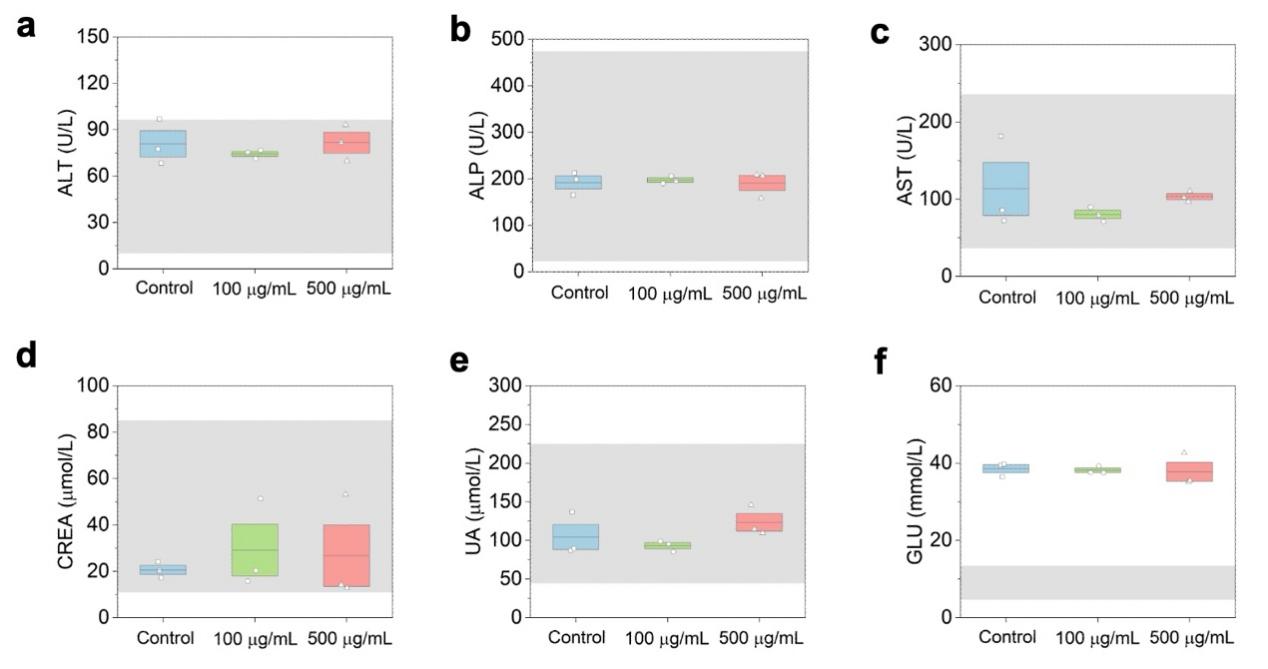
**

**Fig. S63** a-c) Liver function, d,e) kidney function, and f) blood glucose analyses of the diabetic mice on day 31 post-systemic PtSNC injections. Lighter gray bars represent the range of normal values obtained from the healthy mice. Data are expressed as mean ± s.e.m. (*n* = 3 independent experiments)

**
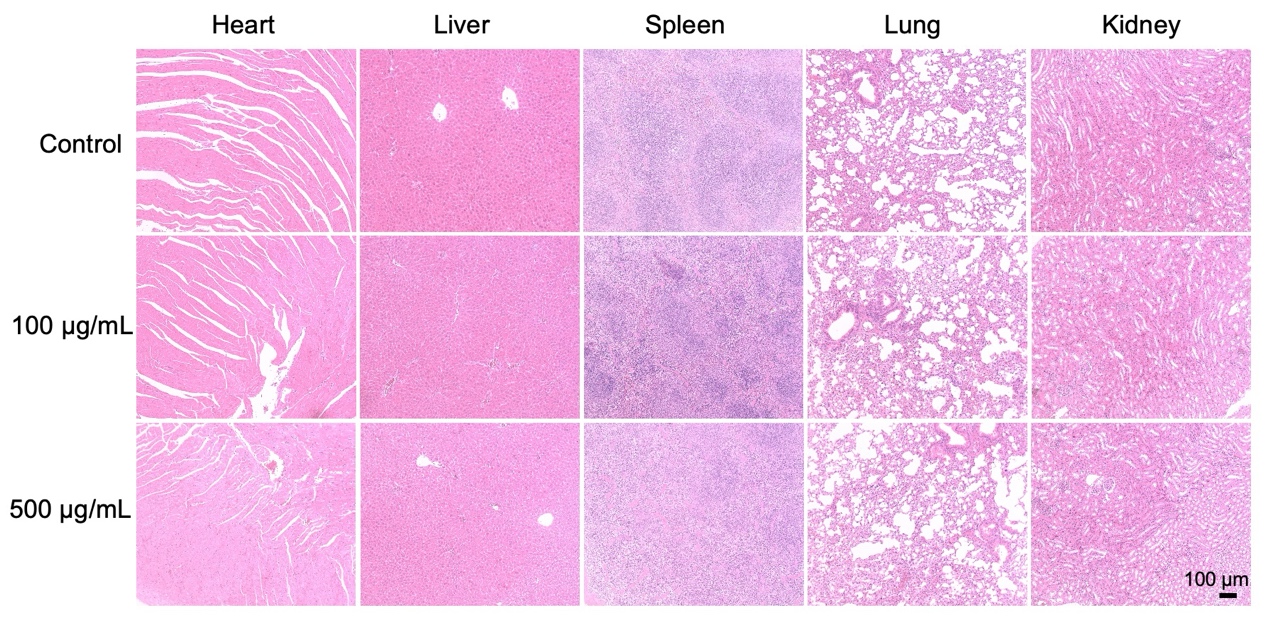
**

**Fig. S64** H&E staining images of the tissues (heart, liver, spleen, lung, and kidney) obtained from the diabetic mice post-systemic PtSNC injections


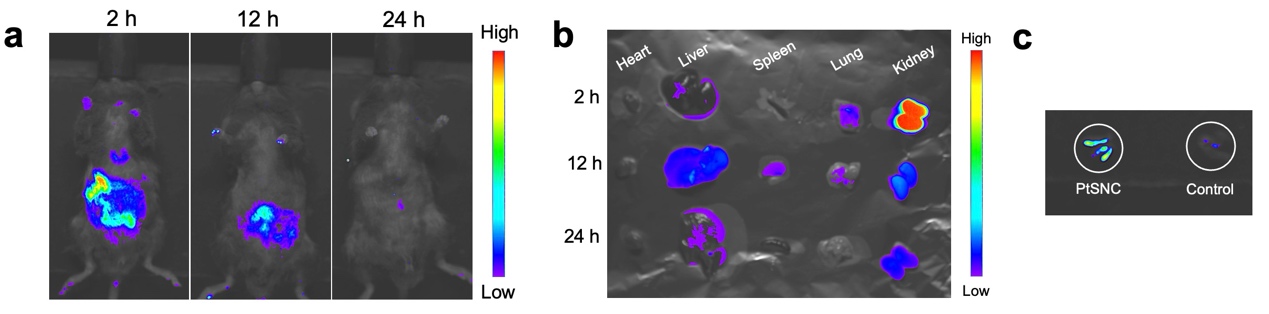


**Fig. S65** a) In vivo and b) ex vivo fluorescence imaging analyses for the diabetic mice after intravenous injection of the Cy5-labeled PtSNC for different time periods. c) The fluorescence image of the feces from the PtSNC-treated mice at 24 h or untreated mice

**S3 Supplementary Tables**

**Table S1** The Pt content in the PtSNC determined by different methods

| **Method** | **Element** | **Wt%** |
| --- | --- | --- |
| EDS | Pt | 3.69% |
| ICP-MS | Pt | 4.15% |
| XPS | Pt | 4.72% |

**Table S2** EXAFS fitting parameters at the Pt L3-edge for various samples

| **Sample** | **Path** | ***CN*** | ***R* (Å)** | ***σ^2^* (Å^2^)** | ***ΔE*_0_ (eV)** | ***R* factor** |
| --- | --- | --- | --- | --- | --- | --- |
| Pt foil | Pt-Pt | 12.00 | 2.75 | 0.0049 | 8.47 | 0.0127 |
| PtO_2_ | Pt-O | 6.00 | 1.99 | 0.0036 | 9.12 | 0.0111 |
| TCPP(Pt) | Pt-N | 4.00 | 2.00 | 0.0051 | 9.18 | 0.0140 |
| PtSNC | Pt-N | 3.91 | 2.01 | 0.0038 | 9.17 | 0.0178 |

*CN*: coordination number. *R*: distance between absorber and backscatter atoms. *σ^2^*: Debye-Waller factor to account for both thermal and structural disorders. *ΔE*_0_: inner potential correction. *R* factor indicates the goodness of the fit. An *R* factor less than 0.02 means a good fit between the data and theory. S_0_^2^ was fixed to 1.0.

**Table S3** List of the used antibodies and their characteristics

| **Protein** | **Primary antibody** | **Manufacturer** | **Item number** |
| --- | --- | --- | --- |
| GPX4 | Rabbit monoclonal | Abcam | ab125066 |
| GAPDH | Mouse monoclonal | HUABIO | EM1101 |
| Arg-1 | Rabbit polyclonal | Servicebio | GB11285 |
| TNF-α | Rabbit polyclonal | Servicebio | GB11188 |
| HIF-1α | Rabbit polyclonal | Servicebio | GB114936 |
| CD31 | Rabbit polyclonal | Servicebio | GB11063-2 |
| AMPK𝛼 | Rabbit polyclonal | Affinity | AF6423 |
| IDH1 | Rabbit polyclonal | Affinity | DF6892 |
| OGDH | Rabbit monoclonal | Abcam | ab307369 |
| PI3K | Rabbit polyclonal | CST | 4292 |
| AKT | Rabbit polyclonal | Abcam | ab38449 |
| *β*-actin | Mouse monoclonal | Proteintech | 66009-1-Ig |
| **Secondary antibody** | | **Manufacturer** | **Item number** |
| Horseradish peroxidase-conjugated goat anti-rabbit IgG | | Jackson | 111-035-003 |
| Horseradish peroxidase-conjugated goat anti-mouse IgG | | Proteintech | SA00001-1 |
| Cy3-conjugated goat anti-rabbit IgG | | Boster | BA1032 |
| Alexa Fluor 488-conjugated goat anti-rabbit IgG | | Servicebio | GB25303 |

**Supplementary References**

1. H. Funke, M. Chukalina, A.C. Scheinost, A new FEFF-based wavelet for EXAFS data analysis. J. Synchrotron Radiat. **14**(Pt 5), 426–432 (2007). <https://doi.org/10.1107/S0909049507031901>
2. B. Ravel, M. Newville, ATHENA, ARTEMIS, *HEPHAESTUS*: data analysis for X-ray absorption spectroscopy using IFEFFIT. J. Synchrotron Radiat. **12**(Pt 4), 537–541 (2005). <https://doi.org/10.1107/S0909049505012719>
3. G. Kresse, D. Joubert, From ultrasoft pseudopotentials to the projector augmented-wave method. Phys. Rev. B **59**(3), 1758–1775 (1999). <https://doi.org/10.1103/physrevb.59.1758>
4. J.P. Perdew, K. Burke, M. Ernzerhof, Generalized gradient approximation made simple. Phys. Rev. Lett. **77**(18), 3865–3868 (1996). <https://doi.org/10.1103/physrevlett.77.3865>
5. S. Grimme, J. Antony, S. Ehrlich, H. Krieg, A consistent and accurate *ab initio* parametrization of density functional dispersion correction (DFT-D) for the 94 elements H-Pu. J. Chem. Phys. **132**(15), 154104 (2010). <https://doi.org/10.1063/1.3382344>
6. P.D. Ottewell, J.K. Woodward, D.V. Lefley, C.A. Evans, R.E. Coleman et al., Anticancer mechanisms of doxorubicin and zoledronic acid in breast cancer tumor growth in bone. Mol. Cancer Ther. **8**(10), 2821–2832 (2009). <https://doi.org/10.1158/1535-7163.MCT-09-0462>
7. L. Huang, J. Chen, L. Gan, J. Wang, S. Dong, Single-atom nanozymes. Sci. Adv. **5**(5), eaav5490 (2019). <https://doi.org/10.1126/sciadv.aav5490>
